# Gene-environment interaction modifies the association between hyperinsulinemia and serum urate levels through *SLC22A12*

**DOI:** 10.1172/JCI186633

**Published:** 2025-03-18

**Authors:** Wataru Fujii, Osamu Yamazaki, Daigoro Hirohama, Ken Kaseda, Emiko Kuribayashi-Okuma, Motonori Tsuji, Makoto Hosoyamada, Yuta Kochi, Shigeru Shibata

**Affiliations:** 1 Division of Nephrology, Department of Internal Medicine, Teikyo University School of Medicine, Tokyo, Japan.; 2 Department of Genomic Function and Diversity, Medical Research Laboratory, Institute for Integrated Research, Institute of Science Tokyo, Tokyo, Japan.; 3 Institute of Molecular Function, Misato, Saitama, Japan.; 4 Laboratory of Human Physiology and Pathology, Faculty of Pharma-Science, Teikyo University, Tokyo, Japan.; 5 Advanced Comprehensive Research Organization, Teikyo University, Tokyo, Japan.

**Keywords:** Genetics, Metabolism, Nephrology, Insulin, Population genetics, Transport

## Abstract

**BACKGROUND:**

Hyperinsulinemia and insulin resistance often accompany elevated serum urate levels (hyperuricemia), a highly heritable condition that triggers gout; however, the underlying mechanisms are unclear.

**METHODS:**

We evaluated the association between the index of hyperinsulinemia and the fractional excretion of urate (FEUA) in 162 outpatients. The underlying mechanisms were investigated through single-cell data analysis and kinase screening combined with cell culture experiments. In 377,358 participants of the UK Biobank (UKBB), we analyzed serum urate, hyperinsulinemia, and salt intake. We also examined gene-environment interactions using single nucleotide variants in *SLC22A12*, which encodes urate transporter 1 (URAT1).

**RESULTS:**

The index of hyperinsulinemia was inversely associated with FEUA independently of other covariates. Mechanistically, URAT1 cell-surface abundance and urate transport activity were regulated by URAT1-Thr408 phosphorylation, which was stimulated by hyperinsulinemia via AKT. Kinase screening and single-cell data analysis revealed that serum and glucocorticoid-regulated kinase 1 (SGK1), induced by high salt, activated the same pathway, increasing URAT1. Arg405 was essential for these kinases to phosphorylate URAT1-Thr408. In UKBB participants, hyperinsulinemia and high salt intake were independently associated with increased serum urate levels. We found that *SLC22A12* expression quantitative trait locus (eQTL) rs475688 synergistically enhanced the positive association between serum urate and hyperinsulinemia.

**CONCLUSION:**

URAT1 mediates the association between hyperinsulinemia and hyperuricemia. Our data provide evidence for the role of gene-environment interactions in determining serum urate levels, paving the way for personalized management of hyperuricemia.

**FUNDING:**

ACRO Research Grants of Teikyo University; Japan Society for the Promotion of Science; the Japanese Society of Gout and Uric & Nucleic Acids; Fuji Yakuhin; Nanken-Kyoten; Medical Research Center Initiative for High Depth Omics.

## Abstract 2

The study demonstrates that the interaction between gene and environment is involved in the elevation of serum urate levels, a critical factor that triggers gout.

## Graphical abstract

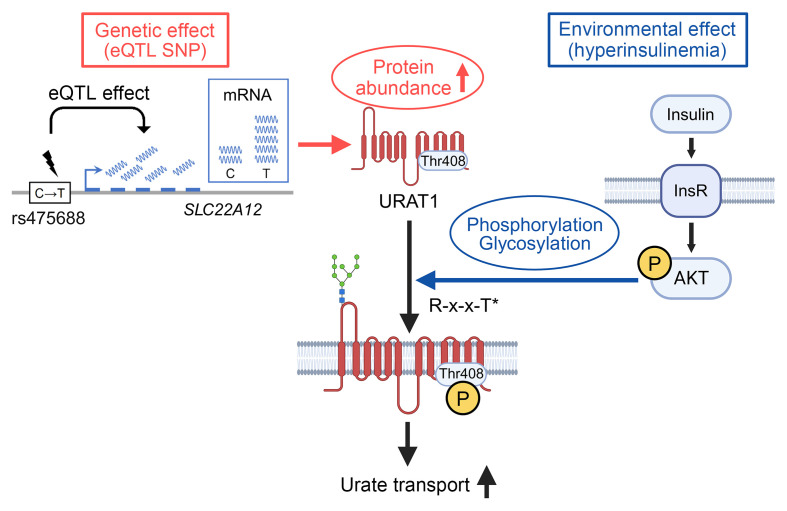

## Introduction

Hyperuricemia (elevated serum urate levels) is a globally increasing condition that triggers gout, which is a severe arthritis caused by the deposition of urate crystals (1). Mendelian randomization studies have shown that hyperuricemia increases the risk of cardiovascular diseases (2). Research to date has revealed that hyperuricemia has 40%–70% of heritable components, with both rare genetic variants having large effects and common SNPs having modest effect sizes playing important roles in this heritability (3–5). Analyses of monogenic disorders, together with population-based studies, have identified the essential genes that regulate serum urate levels, including *SLC22A12* (6, 7) and *SLC2A9* (8). *SLC22A12* encodes urate transporter 1 (URAT1), which reabsorbs urate at the apical membrane of renal tubules through exchange with intracellular lactate and other monocarboxylates (6). Glucose transporter 9 (GLUT9) is encoded by *SLC2A9* and expressed on the basal side of renal proximal tubules (9). Loss-of-function (LOF) mutations in these genes cause renal hypouricemia type 1 and type 2, familial disorders with extremely low levels of serum urate due to renal loss. In particular, renal hypouricemia type 1 due to mutations in *SLC22A12* is considered much more common than type 2 resulting from *SLC2A9* mutations (10, 11). In addition, both common and rare single nucleotide variants (SNVs) in *SLC22A12* have been shown to be the major determinants of serum urate levels and the risk of gout in the general population (12, 13).

Among the various lifestyle factors that affect urate metabolism, obesity is a central element (14). Prospective observational studies have reported that the risk of gout is as much as 16-fold higher in individuals with a BMI of more than 27.5 than in those with a BMI of less than 20 (14). Other risk factors include a purine-rich diet, high alcohol intake, and reduced physical activity (14, 15), all of which are interrelated and are associated with abdominal obesity, metabolic syndrome, and insulin resistance. In addition, there is some evidence that increased salt intake is associated with higher serum urate levels (16), although the data are still controversial (17). On the other hand, a diet rich in fruit has consistently been shown to be associated with lower serum urate levels (14, 18). Concerning the underlying mechanisms, multiple lines of evidence have demonstrated that a strong positive association exists between hyperinsulinemia and hyperuricemia (19–21), indicating that insulin signaling may play a pathogenic role. In support of this possibility, the causal role of hyperinsulinemia in increasing serum urate levels was demonstrated in a Mendelian randomization study (22).

Hyperuricemia in individuals with hyperinsulinemia is mediated by either urate overproduction (23, 24) or underexcretion (25). Several pathophysiological mechanisms have been proposed for the former, including increased xanthine oxidoreductase activity (which produces uric acid) and fructose overconsumption (26, 27). Studies have suggested that hyperinsulinemia alters URAT1 and other renal urate transporters, increasing net urate uptake in the proximal tubules (28–30). However, the details of the molecular mechanisms remain to be elucidated, partly because of the substantial difference in urate metabolism between humans and other mammals (31). In most mammals, including rodents, urate is converted to allantoin by uricase (urate oxidase). In contrast, this enzyme is inactivated by nonsense mutations in humans and several other higher mammals, such as gorillas and chimpanzees (31, 32), making serum urate levels in these animals approximately 3 to 5 times higher than in other animals, such as mice and rats.

However, compared with the progress in the understanding of genetic and environmental factors that influence serum urate levels, the role of the interactions between these factors is far less clear. By combining biochemistry with population genetics, we explored the possibility of gene-environment interactions in human urate metabolism. Using biochemical analysis, we examined the mechanisms by which insulin promotes URAT1-mediated urate transport by focusing on the role of posttranslational modifications, including phosphorylation and glycosylation, in membrane trafficking and urate transport activity. In the genetic analysis, we tested whether the expression quantitative trait locus (eQTL) that influences the expression of *SLC22A12*, as well as its LOF mutation, modifies the association between hyperinsulinemia and hyperuricemia.

## Results

### Insulin resistance is associated with reduced urate excretion from the kidney.

We first evaluated the contribution of urate under excretion in the association between insulin resistance and elevated serum urate levels in our patients. By screening the medical records from 2019 to 2023 according to the criteria described in the Methods section and the flowchart shown in Supplemental Figure 1A (supplemental material available online with this article; https://doi.org/10.1172/JCI186633DS1), we included 162 patients. The mean age was 61.9 years, 62% were male, and the mean serum urate level was 327.5 μmol/L (Supplemental Table 1). Among them, 66% had a history of diabetes mellitus, and the mean HbA1c level was 64.6 mmol/mol. In a regression analysis, we found that homeostasis model assessment as an index of insulin resistance (HOMA-IR) was significantly and inversely associated with fractional excretion of urate (FEUA), an indicator of renal urate excretion (*P* < 0.001; Supplemental Figure 1B), suggesting that insulin resistance is associated with reduced renal urate excretion. In the multiple regression analysis, the association between HOMA-IR and FEUA levels was significant after adjusting for potential confounders, including age, sex, smoking history, alcohol consumption, body mass index, comorbid conditions, and estimated glomerular filtration rate calculated by creatinine (eGFRcre) (Supplemental Table 2). Similar results were obtained when the model was further adjusted for proteinuria and glycosuria (Supplemental Table 3).

### Phosphorylation of URAT1 by AKT at Thr408 (T408) controls trafficking and cell-surface abundance, regulating urate transport.

Although insulin has been suggested to increase URAT1-mediated urate transport (28, 30), the detailed mechanism remains unclear. In renal proximal tubules expressing URAT1, insulin has been shown to act through insulin receptor substrates 2 (IRS2), activating the PI3K/AKT pathway (33). Previously, Endou and colleagues have predicted possible phosphorylation sites in URAT1 by the AGC kinase family (34), although biochemical analysis was not performed. Thus, we focused on the role of URAT1 phosphorylation by AKT, an AGC kinase downstream of IRS2 signaling.

**Figure 1:**
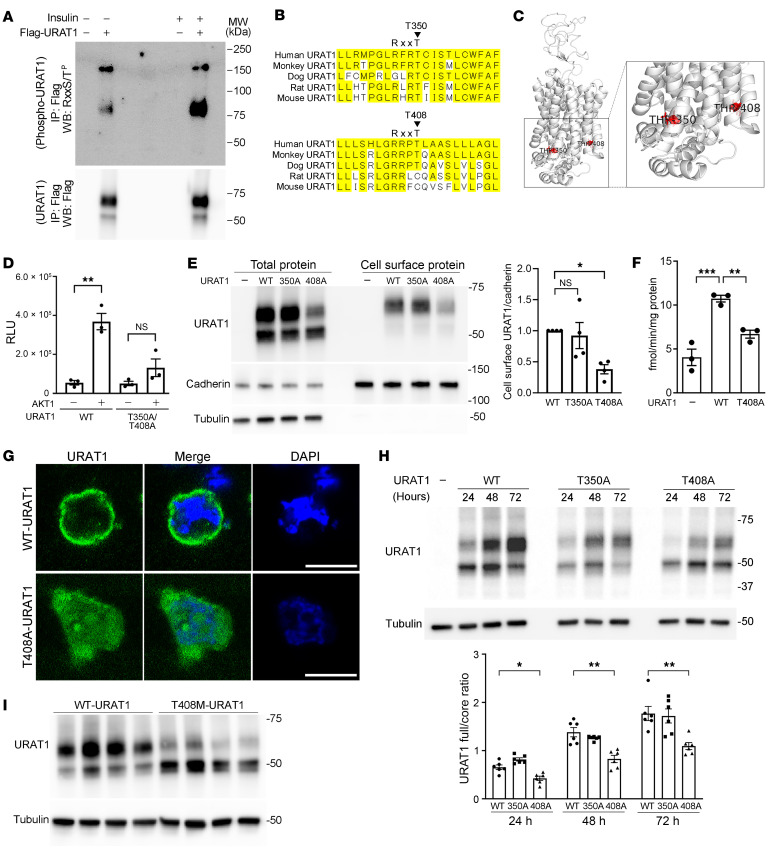
T408 phosphorylation regulates URAT1 trafficking and urate transport activity. (**A**) Flag-tagged, hURAT1 was expressed in HEK293 cells, incubated in the presence or absence of insulin (100 nM for 3 hours), and purified by Flag-IP. Samples were subjected to Western blotting with anti–R-x-x-S^P^/T^P^ and anti-Flag antibodies. (**B**) Candidate phosphorylation sites and alignment among orthologs. (**C**) Location of T350 and T408 in the 3D modeled structure of URAT1. (**D**) WT or nonphosphorylatable T305A/T408A Flag-URAT1 was expressed in HEK cells, purified by IP, and incubated with AKT1 in the presence of ATP. Phosphorylation signal was detected by ADP-glo assay (*n* = 3). (**E**) WT hURAT1 (hURAT1^WT^) and hURAT1 carrying nonphosphorylatable T350A (hURAT1^T350A^) and T408A substitution (hURAT1^T408A^) were expressed in HEK cells. Cell-surface levels were analyzed by cell-surface biotinylation assay. Bar graphs show the results of quantitation from 4 independent experiments. (**F**) Uptake of 10 μM [^14^C]urate was measured in HEK cells expressing no URAT1 (control), HEK cells expressing hURAT1^WT^, and those expressing hURAT1^T408A^ (*n* = 3). (**G**) HEK cells expressing Flag-hURAT1^WT^ and Flag-hURAT1^T408A^ were stained with anti-Flag antibody (green) and DAPI (blue). hURAT1^WT^ is predominantly expressed at or near plasma membrane, whereas hURAT1^T408A^ is cytoplasmic. Scale bars: 10 μm. (**H**) Time course analysis of hURAT1 glycosylation. HEK cells expressing WT or indicated nonphosphorylatable forms of hURAT1 were lysed and analyzed by Western blotting at 24, 48, and 72 hours. Bars graphs show the results of quantitation of fully glycosylated form versus core glycosylated form (*n* = 6). (**I**) Comparison of hURAT1 glycosylation between WT hURAT1 and nonsynonymous single-nucleotide variant (rs146048999; hURAT1 with T408M substitution). Data are represented as mean ± SEM. (**D**) Unpaired *t* test; (**E**, **F**, **H**, and **I**) 1-way ANOVA with Dunnett’s test. *P < 0.05; **P < 0.01; ***P < 0.001.

To evaluate whether human URAT1 (hURAT1) is phosphorylated at sites with a minimal AKT recognition motif (R-x-x-S^P^/T^P^, where x corresponds to any amino acids and S^P^/T^P^ represents the target phosphorylation site) (35), we expressed full-length hURAT1 with an N-terminal Flag in HEK cells. hURAT1 was then purified by Flag-IP and analyzed using a monoclonal anti–R-x-x-S^P^/T^P^ antibody (phospho-AKT substrate antibody; Cell Signaling Technology) that specifically recognizes the phosphorylated Ser and Thr contained in the recognition motif. We detected a robust signal with the antibody at the expected molecular weight of the fully glycosylated form of hURAT1 (12) (Figure 1A). The signal recognized by the anti–R-x-x-S^P^/T^P^ antibody in hURAT1 increased in the presence of insulin, whereas that of total hURAT1 remained unchanged. We then searched for Ser and Thr residues in the R-x-x-S/T motif of hURAT1 and identified 3 candidate sites: T259, T350, and T408. Among these, T259 is predicted to be extracellular and is unlikely to be phosphorylated by intracellular kinases. A comparison of the corresponding sites among different orthologs revealed that T350 is highly conserved across species. In contrast, T408 is present only in higher mammals and not in rats or mice (Figure 1B). Structural modeling indicated that T350 and T408 are located in the intracellular region at or near the membrane (Figure 1C), supporting that these sites are targets of intracellular kinases.

To confirm that AKT directly phosphorylates these sites, full-length, WT hURAT1 (hURAT1^WT^) and hURAT1 carrying nonphosphorylatable Ala substitutions at these sites (hURAT1^T350A/T408A^) were purified from HEK293 cells and incubated with recombinant AKT1. Phosphorylation of hURAT1 was detected using the ADP-Glo assay (36). We found that AKT1 phosphorylated hURAT1^WT^ and that this signal was abolished in hURAT1^T350A/T408A^, confirming that AKT phosphorylates hURAT1 at these sites (Figure 1D).

We analyzed the functional consequences of hURAT1 phosphorylation. Nonphosphorylatable forms of hURAT1 carrying an Ala substitution for each of these phosphorylation sites (hURAT1^T350A^ and hURAT1^T408A^) were expressed in HEK cells, and the cell-surface levels of hURAT1 were analyzed by a cell-surface biotinylation assay. While the cell-surface levels of hURAT1^T350A^ were comparable to those of hURAT1^WT^, hURAT1^T408A^ showed a significant decrease in cell-surface levels compared with hURAT1^WT^ (Figure 1E). To determine whether the reduced cell-surface levels of hURAT1^T408A^ were accompanied by a change in urate transport, we measured [^14^C]urate uptake in HEK cells expressing hURAT1^WT^ and those expressing hURAT1^T408A^. We found that the [^14^C]urate transport observed in cells expressing hURAT1^WT^ was significantly attenuated in cells expressing hURAT1^T408A^ (Figure 1F). Using confocal microscopy, we confirmed that hURAT1^T408A^ was distributed in the cytoplasm (Figure 1G).

We then analyzed whether nonphosphorylatable Ala substitutions at T350 and T408 affected hURAT1 glycosylation and maturation. In total cell lysates, hURAT1 was detected as a fully glycosylated form at approximately 75 kDa and a core glycosylated form at 50 kDa. Incubation of hURAT1 with peptide *N*-glycosidase F (PNGase F), which cleaves N-linked oligosaccharides, confirmed that these signals represent glycosylated forms (Supplemental Figure 2). We then expressed hURAT1^WT^, hURAT1^T350A^, and hURAT1^T408A^ in HEK cells and quantified glycosylated hURAT1 levels by Western blotting at 24, 48, and 72 hours after transfection. Both hURAT1^WT^ and hURAT1^T350A^ showed a time-dependent increase in the fully glycosylated form, which was the dominant form at 72 hours. However, the ratio of fully glycosylated to core glycosylated forms was significantly decreased in hURAT1^T408A^ (Figure 1H). Consistent with these data, biochemical analysis of a rare SNV (rs146048999) causing a Thr-to-Met mutation at position 408 (T408M) revealed that this variant also exhibited severely impaired glycosylation (Figure 1I), confirming that T408 is the key amino acid that determines hURAT1 function.

### T408 in URAT1 is phosphorylated in human kidney.

**Figure 2:**
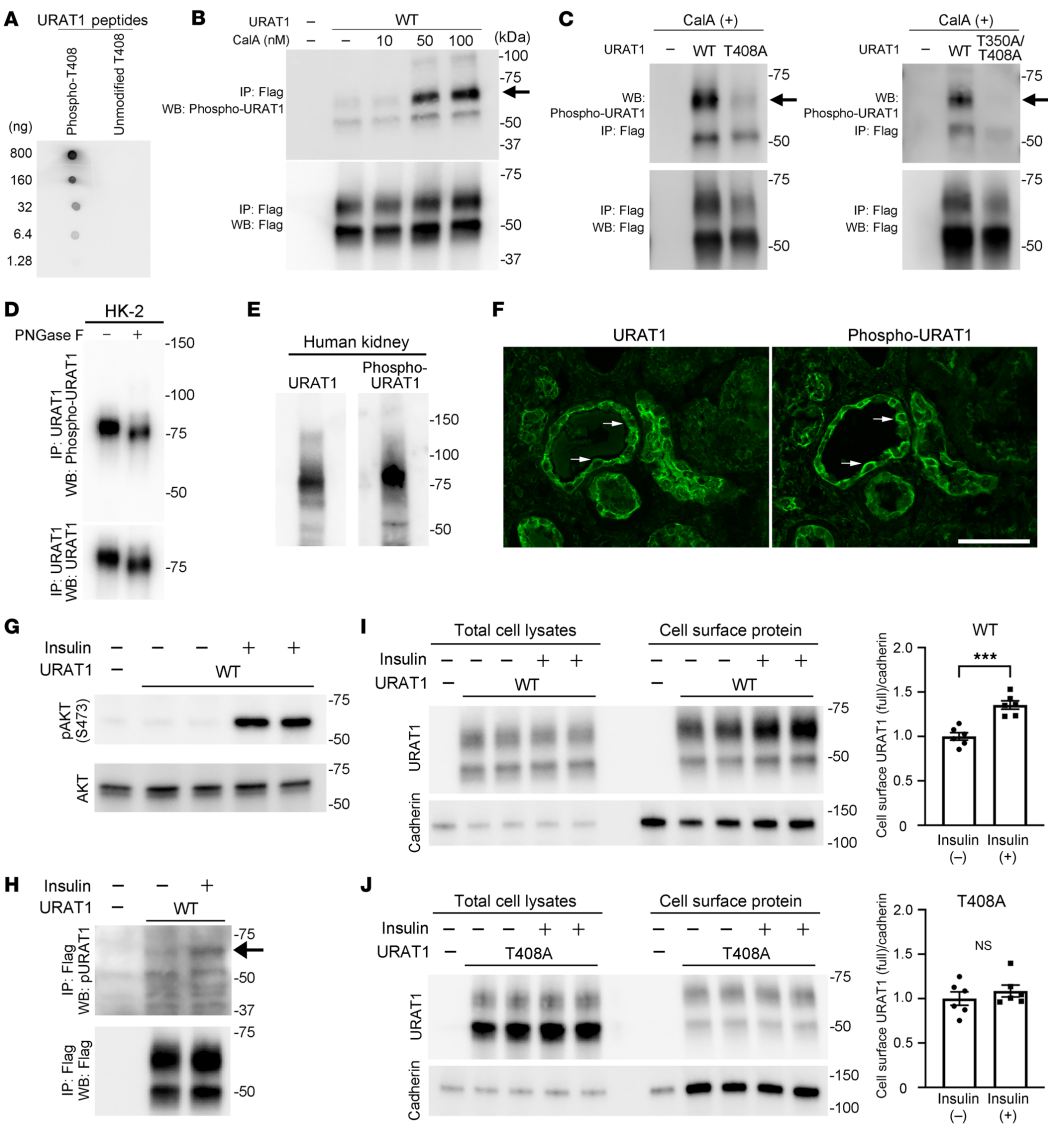
URAT1-T408 is phosphorylated in human kidney and mediates insulin-induced URAT1 trafficking to the cell surface. (**A**) Antibody against hURAT1 phosphorylated at T408 (hURAT1^T408-P^) was incubated with hURAT1 peptide phosphorylated at T408 and nonphosphorylated hURAT1 peptide spotted on a membrane, followed by incubation with peroxidase-conjugated anti-rabbit antibody. Signals were visualized by ECL. (**B**) HEK cells expressing Flag-hURAT1^WT^ were incubated with calyculin A. Cells were lysed and purified by Flag-IP. Phospho-hURAT1 (arrow) and total hURAT1 levels were detected by Western blotting. (**C**) HEK cells expressing hURAT1^WT^, hURAT1^T408A^, and hURAT1^T350A/T408A^ were incubated with 50 nM of calyculin A, lysed, and purified by Flag-IP. Phosphorylated and total hURAT1 levels were detected by Western blotting. Arrows indicate hURAT1^T408-P^. (**D**) Endogenous hURAT1 was purified from lysates of human proximal tubule cell line HK-2 cells by IP using URAT1 antibody, incubated with and without PNGase F, followed by Western blotting using phospho-hURAT1 and hURAT1 antibodies. (**E**) Detection of total and phosphorylated forms of hURAT1 in the human kidney by Western blotting. (**F**) Immunofluorescence photomicrographs of total hURAT1 and phospho-hURAT1 in adjacent sections of the human kidney. Scale bar: 50 μm. (**G**) HEK cells expressing hURAT1^WT^ were incubated in the absence or presence of insulin at 100 nM. Lysates were analyzed by the indicated antibodies. (**H**) HEK cells expressing hURAT1^WT^ were incubated with insulin and lysates were purified by IP, followed by Western blot analysis using phospho-hURAT1 antibody. Arrow indicates hURAT1^T408-P^. The bottom panel shows the total hURAT1. (**I** and **J**) WT (**I**) and nonphosphorylatable T408A (**J**) hURAT1 were expressed in HEK cells and were incubated with insulin. Cell-surface levels of hURAT1 were determined by cell-surface biotinylation assay. Bar graphs show the results of quantitation (*n* = 6). Data are represented as mean ± SEM. (**I** and **J**) Unpaired *t* test. ***P < 0.001.

To further characterize the role of hURAT1 phosphorylation at residue T408 (hURAT1^T408-P^), we produced a polyclonal antibody recognizing this site. The ELISA results indicated that the purified phospho-specific antibody was highly specific for the hURAT1 peptide phosphorylated at T408 (Supplemental Figure 3). In addition, the dot blot assay showed that this antibody robustly recognized the hURAT1 peptide phosphorylated at T408; in contrast, the signal was virtually absent when the antibody was incubated with the hURAT1 peptide with unmodified, nonphosphorylated T408 (Figure 2A). Western blot analysis using this antibody with immunopurified, full-length hURAT1^WT^ from HEK cells revealed a signal at the expected molecular weight of fully glycosylated hURAT1, which was sharply increased by treatment with calyculin A, a phosphatase inhibitor, without altering total hURAT1 abundance (Figure 2B). Moreover, this signal was abolished in samples not expressing hURAT1 or expressing hURAT1 in which T408 was mutated to Ala (arrows in Figure 2C), confirming that the antibody selectively recognizes hURAT1^T408-P^. An additional signal at the expected molecular weight of the core glycosylated form of hURAT1 (approximately 50 kDa) likely indicated hURAT1 phosphorylation at T350 because the signal was attenuated by the T350A substitution (Figure 2C). Given these results, we infer that the phosphorylation of hURAT1^T408^ is associated with full glycosylation and maturation, whereas T350 is phosphorylated at an earlier step in protein processing. These results are also in line with our earlier results demonstrating that the nonphosphorylatable Ala substitution at T408 impairs the glycosylation and maturation of hURAT1 (Figure 1H).

To determine whether endogenous hURAT1 was phosphorylated, we performed 3 distinct experiments. First, we purified endogenous hURAT1 from the lysates of HK-2 cells, a human proximal tubule cell line that expresses endogenous hURAT1 (37, 38). We then performed Western blotting using the phospho-URAT1 antibody developed in our laboratory. The results demonstrated that the antibody recognized the fully glycosylated form of endogenous hURAT1 (Figure 2D). In addition, PNGase F treatment caused a mass shift in both the phosphorylated and total forms of hURAT1 (Figure 2D), which was consistent with the observation that hURAT1^T408-P^ was present in the fully glycosylated form of hURAT1. Second, we evaluated hURAT1^T408-P^ abundance in the human kidney using Western blot analysis. We found that URAT1^T408-P^, as well as total URAT1, was robustly present in human kidney lysates (Figure 2E). Third, the localization of hURAT1^T408-P^ was determined in human kidney sections by immunofluorescence using the phospho-URAT1 antibody. The results demonstrated that hURAT1^T408-P^ was present in the apical membrane of renal proximal tubules that were positive for URAT1 (Figure 2F). The different staining patterns of URAT1 and phospho-URAT1 indicated the possibility of cell-selective modifications. These data confirm that URAT1^T408-P^ is present in the human kidney.

### Insulin regulates cell-surface levels of hURAT1 through T408 phosphorylation.

Next, we tested to determine whether insulin could increase the cell-surface abundance of hURAT1 by promoting T408 phosphorylation. HEK cells expressing Flag-hURAT1^WT^ were incubated with insulin (100 nM) for 3 hours. Insulin robustly increased AKT phosphorylation at S473 (pAKT), the phosphorylation site in the kinase domain essential for catalytic activity (39) (Figure 2G). We purified hURAT1 from HEK cells by IP and analyzed hURAT1^T408-P^ abundance. Western blot analysis showed that hURAT1^T408-P^ levels were increased by insulin (Figure 2H). To determine the causal effect of hURAT1^T408-P^ induction, we compared the effects of insulin on hURAT1^WT^ and nonphosphorylatable hURAT1^T408A^. HEK cells expressing hURAT1^WT^ and hURAT1^T408A^ were incubated with 100 nM insulin for 3 hours, and the cell-surface levels of URAT1 were evaluated by cell-surface biotinylation assay. We found that insulin significantly increased the cell-surface abundance of hURAT1 in HEK cells expressing hURAT1^WT^ (Figure 2I); however, this effect was abolished in HEK cells expressing hURAT1^T408A^ (Figure 2J), demonstrating the causal role of T408 phosphorylation in insulin-mediated hURAT1 regulation. We also confirmed that insulin at a lower concentration (1 nM) modestly but significantly increased the cell-surface levels of hURAT1^WT^, but not those of hURAT1^T408A^, indicating a dose-dependent effect (Supplemental Figure 4).

### Kinase screen and single-cell analysis identify SGK1 as an alternative regulator of URAT1.

**Figure 3:**
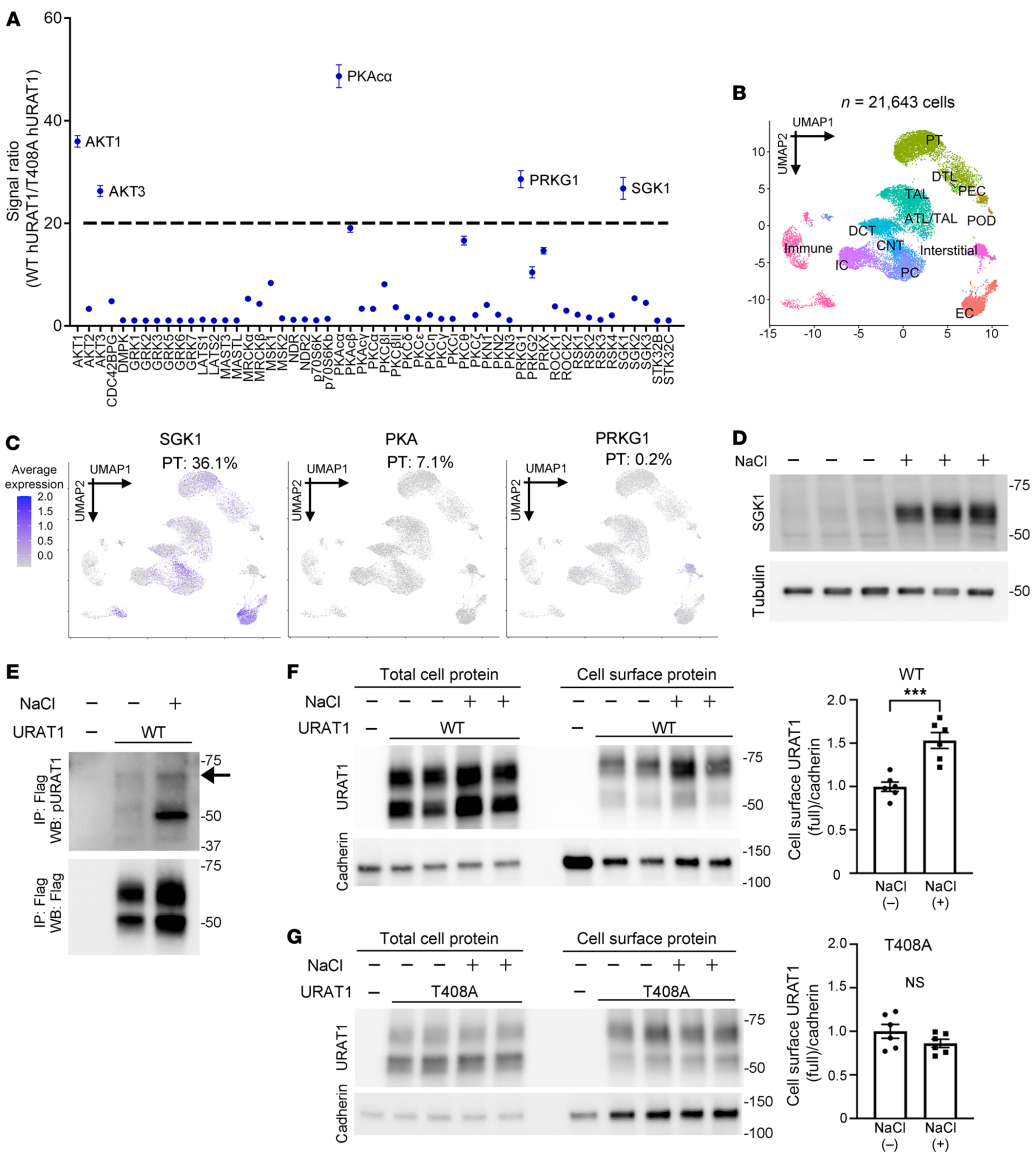
Kinase screen and single-cell analysis identify SGK1 as an alternative regulator of URAT1. (**A**) Kinase screen assay. Human URAT1 peptide containing Thr408 and nonphosphorylatable Ala408 hURAT1 peptide were synthesized and incubated with 53 recombinant AGC kinases in triplicate. Phosphorylation signal was detected by ADP-glo assay. The mean phosphorylation signal of hURAT1 T408:URAT1 A408 after incubation with each kinase is indicated as a dot. (**B**) UMAP showing 13 kidney cell clusters from single-cell RNA-Seq data analysis (see Methods). (**C**) Feature plots showing the expression of SGK1 (left), PKA (middle), and PRKG1 (right) in the kidney cells from living donor subjects. Numbers indicate percentage of expression in proximal tubule (PT) cells. (**D**) HEK cells were incubated in the absence or presence of NaCl at 75 mM for 3 hours. Lysates were analyzed by the indicated antibodies. SGK1 abundance is sharply increased by NaCl. (**E**) HEK cells expressing WT hURAT1 were incubated in the presence of NaCl as described in **D** and the lysates were purified by IP, followed by Western blot analysis using phospho-hURAT1 antibody. Arrow indicates hURAT1^T408-P^. The lower band detected at around 50 kDa represents hURAT1 phosphorylated at T350 (see text). The bottom panel shows the total hURAT1 levels. (**F** and **G**) HEK cells expressing WT (**F**) and nonphosphorylatable T408A (**G**) forms of hURAT1 were incubated with NaCl at 75 mM for 3 hours. Cell-surface levels of hURAT1 were determined by cell-surface biotinylation assay followed by Western blotting. Bar graphs show the results of quantitation (*n* = 6). Data are represented as mean ± SEM. (**F** and **G**) Unpaired *t* test. ***P < 0.001.

Given that many phosphorylation sites overlap among the AGC family kinases, we performed a kinase-screening assay involving diverse AGC family kinases. We synthesized a hURAT1 peptide containing T408 and a nonphosphorylatable T408A hURAT1 peptide. Each of these peptides was separately incubated with 53 cloned human AGC kinase domains (Kinexus) in triplicate; the ratio of phosphorylation between hURAT1^WT^ and hURAT1^T408A^ for each kinase was calculated (Figure 3A; a full list of kinases and raw data are shown in Supplemental Table 4). The kinase-screening assay revealed 13 kinases with a ratio of greater than 5, 9 kinases with a ratio of greater than 10, and 5 with ratios greater than 20. AKT1 and its isozyme AKT3 were among the 5 kinases with ratios greater than 20. Other kinases included protein kinase A (PKA), protein kinase G (PRKG), and serum and glucocorticoid-regulated kinase 1 (SGK1). Among the identified 3 kinases, the transcriptome analysis of 21,643 individual cells from human kidneys in the National Institute of Diabetes and Digestive and Kidney Diseases (NIDDK) Kidney Precision Medicine Project (KPMP) repository revealed that SGK1 was expressed in 36.1% of proximal tubules, whereas PKA and PRKG expression were low at 7.1% and 0.2%, respectively (Figure 3, B and C).

Proximal tubules are anatomically subdivided into 3 segments (S1 to S3), and a previous transcriptome analysis of microdissected renal tubules (40) revealed that *Slc22a12* expression in the S3 segment is 75-fold higher than that in the initial S1 segment (Supplemental Table 5). The S3 segment extends into the outer medulla of the kidney, where the tubules are exposed to higher levels of NaCl in the interstitium, which is accumulated by countercurrent multiplication (41). NaCl and other osmolytes induce SGK1 expression via mechanisms involving p38/MAPK and NFAT5 (also known as TONEBP) (42–44). Based on these data, we tested to determine whether NaCl increases hURAT1 levels through SGK1 and T408 phosphorylation. As expected, the addition of 75 mM NaCl to the medium robustly increased SGK1 protein levels in HEK cells (Figure 3D). In hURAT1 immunopurified from HEK cells exposed to high NaCl concentrations, we found that hURAT1^T408-P^ levels (along with hURAT1 phosphorylation at T350) were increased compared with those in the control (Figure 3E). Moreover, NaCl loading increased the cell-surface levels of hURAT1^WT^ but not of hURAT1^T408A^ (Figure 3, F and G). These results indicate that in addition to AKT, SGK1 can phosphorylate and regulate hURAT1.

Next, we evaluated the biochemical mechanisms by which AKT1 and SGK1 recognize hURAT1 at T408 as a substrate. Given that T408 has an Arg at –3 position, a recognition motif for AGC kinases such as AKT (45), we created a synthetic WT hURAT1 peptide containing T408, a nonphosphorylatable hURAT1-T408A peptide, and a hURAT1-R405A peptide (which lacks Arg at –3 position). We then separately incubated these peptides with AKT1 in triplicate and determined the phosphorylation signal using the ADP-Glo assay. The results demonstrated that the robust signal observed in the WT hURAT1 peptide was almost completely eliminated by the R405A substitution (Supplemental Figure 5A). Similar results were observed for SGK1 (Supplemental Figure 5B). To obtain further support for the functional importance of the R-x-x-T motif, we conducted a biochemical analysis of rare SNVs (rs563239942), causing R405C and R405G substitutions, and found that these hURAT1 variants consistently showed impaired maturation (Supplemental Figure 5C). These data confirm that both AKT1 and SGK1 recognize the R-x-x-T motif in hURAT1, phosphorylating T408 and promoting hURAT1 maturation.

### Triglyceride-glucose index and habitual salt intake independently associate with increased serum urate levels in the UK Biobank cohort.

The above experimental data unexpectedly demonstrated that SGK1, a salt-sensing kinase (46, 47), increases hURAT1 at the cell surface, promoting urate reabsorption. Previous studies have shown that a high-salt diet increases renal outer medullary osmolality (48, 49). There is also evidence that dietary salt regulates SGK1 expression in the kidney (50). To extend our observations from the cell-culture experiments to human data, we next investigated whether high salt intake is independently associated with the elevated serum urate levels in UK Biobank (UKBB) data comprising 377,358 participants. A flowchart of participant selection and the baseline characteristics included in the analysis are presented in Supplemental Figure 6A and Supplemental Table 6, respectively. In univariable analysis, serum urate levels and the triglyceride-glucose (TyG) index, a marker of insulin resistance (51, 52), showed a significant positive correlation (*r* = 0.342, *P* < 0.001). In addition, serum urate levels were significantly different among the groups according to their salt-intake habits (Supplemental Table 6). We then performed multivariable analyses to investigate whether the TyG index and habitual salt intake were independently associated with serum urate levels, adjusting for other clinical factors. After controlling for age, sex, and ethnicity, both the TyG index and salt intake habits were significantly associated with higher serum urate levels (multivariable regression model, Model 1). The association was significant after further adjustment with smoking status, drinking habits, and waist-to-hip ratio (Model 2) and additional adjustment with mean blood pressure, HbA1c, total cholesterol, and estimated glomerular filtration rate based on creatinine (eGFRcre) (Model 3) (Table 1).

Furthermore, we evaluated whether these associations were affected by several other lifestyle factors. When accounting for physical activity (metabolic equivalent of task [MET] minutes per week) as an additional covariate, the associations remained significant (Supplemental Table 7). In addition, in another model adjusted for fruit intake, which is a dietary factor associated with lower serum urate levels (14, 18), the associations were consistently significant (Supplemental Table 7). Although the effect size was modest in habitual salt intake compared with that in the TyG index, these data are consistent with our cell-culture data showing that NaCl positively regulates URAT1.

Given the evidence that high salt intake can aggravate insulin resistance (53), we conducted a mediation analysis using Model 3 (54). The results indicated that the TyG index explained 9.2% of the association between habitual salt intake and serum urate levels, whereas the remaining components were considered direct (Supplemental Table 8 and Supplemental Figure 6B).

### Gene-environment interaction between a variant of SLC22A12 and TyG index synergistically increases serum urate levels.

Given the experimental data showing that hyperinsulinemia promotes membrane trafficking of URAT1 and the robust association between the TyG index and serum urate levels in the UKBB data (Table 1), we postulated that gene-environment interactions could modify the association between hyperinsulinemia and hyperuricemia. To test this, we searched the Global Urate Genetics Consortium (GUGC) cohort (4), which included individuals of European ancestry who were independent of the UKBB cohort, and identified 7 SNPs within the *SLC22A12* gene that were significantly associated with serum urate levels. Among those SNPs, the genotype information was available for 1 SNP, rs475688 (C>T), in the UKBB dataset; the T-allele frequency of rs475688 was 25.2%, with T-allele possession significantly associated with increased serum urate levels (effect size: 3.58 μmol/L, *P* = 4.54 × 10^–80^) (55). In a meta-analysis of human kidney eQTLs (56), the T-allele number was significantly associated with increased *SLC22A12* mRNA expression (effect size: 0.197, *P* = 1.44 × 10^–9^). These results suggest that this SNP has a positive eQTL effect on *SLC22A12*, resulting in elevated serum urate levels by upregulating renal urate reabsorption via URAT1.

**Figure 4:**
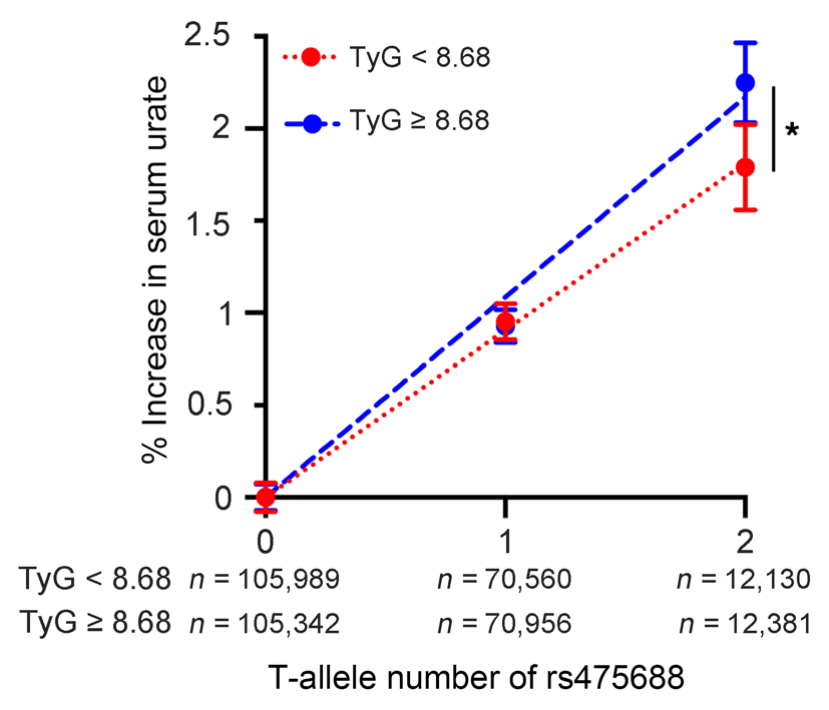
Relationship between rs475688 T-allele number and serum urate levels in 377,358 participants of UKBB study according to TyG index, an indicator of hyperinsulinemia. The study participants were divided into 2 groups based on the median value of TyG index (8.68). The *x* axis represents rs475688 T-allele number and *y* axis represents percentage increase in serum urate levels (compared with individuals with 0 T-allele in each group; mean serum urate levels in individuals with 0 T-allele were 283.7 μmol/L and 329.9 μmol/L for lower and higher TyG groups, respectively). Error bars indicate the SEM and dashed lines represent linear regression lines (with the *y* intercept set to 0) for the percentage change in serum urate levels according to the number of T-alleles of rs475688. Statistical difference in the regression coefficients between the higher and lower TyG index groups was analyzed using Welch’s *t* test. *P < 0.05.

Consistently, in 377,358 participants in the UKBB cohort, the rs475688 T-allele number was significantly associated with elevated serum urate levels. As shown in Table 2, serum urate levels in individuals without the rs475688 T-allele were 306.7 ± 79.0 μmol/L, whereas they increased to 309.7 ± 79.3 μmol/L in those with 1 T-allele, and further increased to 313.3 ± 80.2 μmol/L in those with 2 T-alleles (*P* < 0.001). To address whether the quantitative effect of the rs475688 T-allele number on serum urate levels is influenced by hyperinsulinemia, we divided the study participants into 2 groups based on the median TyG index value (8.68), with each group comprising 188,679 individuals. We then plotted the increase in serum urate levels per T-allele number and performed linear regression analysis. The results indicated a significant difference in the regression coefficients between the high and low TyG index groups (Figure 4; *P* = 0.038). To validate the analysis, we introduced rs475688 T-allele numbers and an interaction term between the rs475688 T-allele number and TyG index into the above-mentioned, multivariable regression model (Model 3). As the effect of this interaction term was positive and statistically significant (β = 0.59, *P* = 0.033), this gene-environment interaction synergistically increased serum urate levels.

Based on these results, we tested to determine whether LOF mutations in *SLC22A12* attenuate the influence of the TyG index on serum urate elevation. For this purpose, we searched previous studies that functionally characterized rare variants of *SLC22A12* and identified 3 variants that are also included in the UKBB database (Supplemental Table 9). Among the 3 SNVs, imputed information (INFO) scores or rs141570522 and rs150255373 were less than 0.8, suggesting that the imputation was not accurate for these variants. Thus, we focused on rs147647315 (G>A) with an INFO score of 1; the A-allele frequency is approximately 0.068% in the entire UKBB cohort. This variant causes an Arg-to-His mutation at position 434 (R434H), resulting in reduced cell-surface levels and impaired urate uptake capacity (57).

As shown in Supplemental Table 10, serum levels were significantly lower in individuals with the rs147647315 mutant allele(s), with stronger effects than the common rs475688 SNP. In the multivariable analysis adjusted for other covariates (as described above), there was a trend toward significance for the interaction between the TyG index and rs147647315 risk allele possession (β = –7.50, *P* = 0.096). Thus, in contrast to rs475688, rs147647315 within *SLC22A12* tended to synergistically diminish the effects of hyperinsulinemia on serum urate levels.

In addition to LOF in *SLC22A12*, mutations in *SLC2A9*, which encodes GLUT9, cause renal hypouricemia (renal hypouricemia type 2), indicating its importance in human urate metabolism. Therefore, we investigated whether a similar interaction is observed in *SLC2A9*. In the GUGC cohort, we identified 2 SNPs, rs4529048 and rs10939650, within *SLC2A9* that were significantly associated with serum urate levels and that had a significant positive eQTL effect in the kidney (56). Because these 2 SNPs were in linkage disequilibrium (*r^2^* = 0.99), we focused on the former. As expected, the number of rs4529048 risk allele (A-allele) was associated with elevated serum urate levels in 377,358 individuals in the UKBB cohort (Supplemental Table 11). We then conducted a similar interaction analysis and found that the interaction term between the rs4529048 A-allele number and the TyG index was significantly negative (β = –1.16, *P* < 0.001). These data suggest that positive interactions were unique to *SLC22A12* and underscore the multifactorial nature of the association between hyperinsulinemia and hyperuricemia.

## Discussion

**Figure 5:**
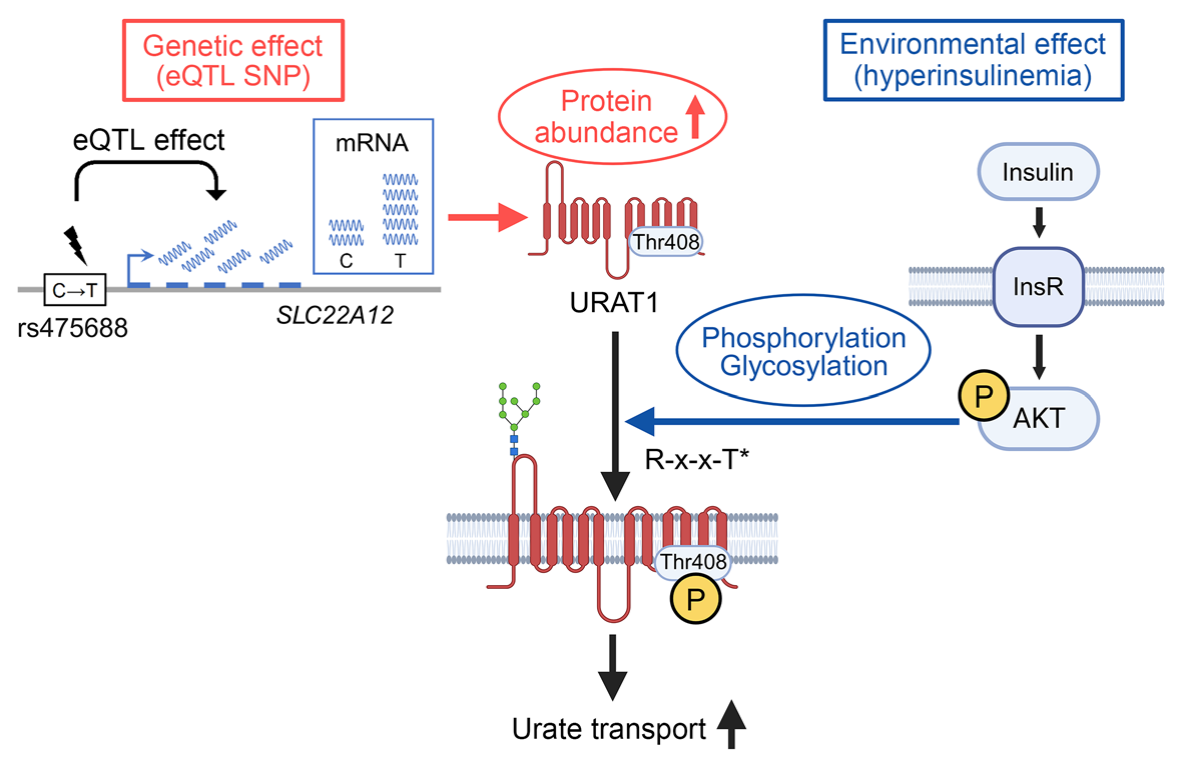
Molecular basis of the gene-environment interaction regulating hURAT1. SNPs with eQTL effects (e.g., rs475688) upregulate *SLC22A12* mRNA expression, leading to increased hURAT1 abundance. Environmental factors include posttranslational modifications, in which AKT kinase, activated by insulin signaling, induces the phosphorylation of URAT1 at Thr408 contained in the R-x-x-T motif. This phosphorylation event promotes glycosylation and enhances the cell-surface abundance of hURAT1. These factors synergistically augment hURAT1 activity, modifying the association between hyperinsulinemia and serum urate levels.

In this study, we showed that URAT1 mediates the well-documented association between hyperinsulinemia and hyperuricemia and that genetic predisposition to altered URAT1 function influences this association. Biochemical analysis revealed 2 phosphorylation sites in URAT1: T350 and T408. Phosphorylation of URAT1 at the latter site promotes glycosylation, cell-surface expression, and urate transport, which is abolished by a nonphosphorylatable Ala substitution. We confirmed that URAT1 phosphorylated at T408 is present in the proximal tubules of the human kidney and that insulin, as well as salt loading, increases URAT1 phosphorylation and cell-surface levels. The clinical significance of these findings was confirmed in a large population study comprising 377,358 individuals with UKBB. Moreover, we found a positive association between the TyG index, an indicator of hyperinsulinemia, and serum urate levels, which was synergistically augmented by a SNP with an eQTL effect on *SLC22A12*, whereas a rare LOF SNV in *SLC22A12* tended to synergistically diminish the association, demonstrating the gene-environment interaction (Figure 5).

Interactions between genetic and environmental factors have been demonstrated in several noncommunicable diseases. For example, in overweight and obesity, Young et al. have shown in the UKBB cohort that there is an interaction between *FTO* rs1421085 and lifestyle factors, such as physical activity, sleep duration, and alcohol consumption (58). The C-allele in the rs1421085 SNP disrupts the conserved motif of AT-rich interaction domain 5B, a transcription repressor in preadipocytes, activating *IRX3* and *IRX5*, which reduces mitochondrial thermogenesis (59). Regarding the interaction between urate transporters and lifestyle factors, Batt et al. reported an interaction between a SNP in *SLC2A9* and high-fructose corn syrup consumption in the risk of gout (60). The study included participants from New Zealand (925 gout cases and 709 controls) and the Atherosclerosis Risk in Communities (ARIC) study (148 cases and 6927 controls) and indicated that individuals carrying the gout-protective allele in *SLC2A9* showed a tendency toward increased effect of fructose consumption upon gout and serum urate elevation (60). However, genetic influence on the association between hyperuricemia and hyperinsulinemia has not been previously studied, to our knowledge. Thus, our study demonstrates a previously unrecognized *SLC22A12* genotype-specific effect on serum urate levels in association with insulin resistance and hyperinsulinemia using a large-scale biomedical database from the UKBB. The critical role of *SLC22A12* encoding URAT1 in the association between the 2 states was further confirmed by our detailed functional analysis.

Although we showed the causal role of URAT1 in hyperinsulinemia resulting in hyperuricemia, our data also suggest that other mechanisms coexist. Interestingly, the TyG index was lower in individuals with URAT1-R434H, a mutation associated with lower serum urate levels. A previous randomized controlled study showed that urate-lowering therapy improves insulin resistance in patients with asymptomatic hyperuricemia (61). These data support a modest but significant effect of serum urate on insulin resistance. In addition, given that URAT1 is functionally linked to Na^+^-dependent monocarboxylate transporters (SMCTs) (62), alterations in other Na^+^-coupled transporters in this segment, such as Na^+^-glucose cotransporters (SGLTs) (63, 64), can indirectly influence urate transport through URAT1. Additionally, the urate transporter GLUT9 in the kidney is stimulated by insulin (29) and can modulate the association between hyperinsulinemia and hyperuricemia. Indeed, our analysis focusing on rs4529048 within *SLC2A9* showed a negative interaction between the TyG index and number of rs4529048 risk alleles. Although the mechanisms remain unclear, previous studies have shown that, in addition to the kidney, GLUT9 is highly expressed in the liver, contributing to the sinusoidal efflux of urate (65, 66). Given these data, liver dysfunction associated with overweight and hyperinsulinemia may negatively affect GLUT9-mediated urate efflux in the liver. Although our study showed that insulin increases the cell-surface levels of URAT1 through AKT-mediated phosphorylation, stimulating urate transport, the data also highlight the multifactorial nature of the association between hyperinsulinemia and hyperuricemia.

In this study, we identified 2 phosphorylation sites in hURAT1, T350 and T408, which can be targeted by AGC kinases. Phosphorylation at these sites is consistent with the prediction of Endou et al. (34), although there is little experimental evidence. Consistent with previous data showing that URAT1 undergoes extensive glycosylation (12), we detected 2 distinct forms of URAT1 by Western blot analysis and confirmed that these signals represent the fully and core glycosylated forms, respectively. Detection of URAT1^T408-P^ at the molecular weight of the fully glycosylated form, together with our observation that the nonphosphorylatable Ala substitution impairs glycosylation and translocation to the cell surface, demonstrated that phosphorylation at T408 is necessary for the maturation and full function of URAT1. In contrast, Ala substitution experiments suggested that phosphorylation at T350 may be dispensable for the regulation of URAT1 trafficking and activity. The fact that URAT1^T408^ is conserved in humans, monkeys, and dogs but not in rats or mice underscores the substantial species-specific differences in urate metabolism. In addition, we demonstrated that Arg at the –3 position is essential for the recognition of T408 as a substrate of AKT1 and SGK1.

Although the reason why insulin promotes urate transport through URAT1 is unclear, we inferred that this may be related to the antioxidant properties of urate (67). Glucose loading can promote oxidative stress through several mechanisms, including the formation of advanced glycation end products, superoxide generation in the mitochondria, reduction in NADPH, and reduced glutathione levels (68). Insulin, stimulated by high plasma glucose levels, may promote renal urate reabsorption to counteract the increased oxidative stress during glucose loading. Similarly, the induction of URAT1 by high salt intake may be related to the fact that oxidative stress can be increased by high salt intake, a condition associated with insulin resistance (69).

The strength of this study is that we demonstrated a gene-environment interaction in human urate metabolism through large-scale genetic analysis, coupled with detailed biochemical characterization of the molecular basis linking hyperinsulinemia with hyperuricemia. To our knowledge, few studies have successfully demonstrated gene-environment interactions in complex traits, encompassing both epidemiological and molecular aspects. Therefore, the elucidation of such interactions can enable the prediction of the occurrence of metabolic disorders associated with lifestyle changes, based on disease-risk genotypes. Genetic information can also be used to maximize the efficiency of lifestyle modifications and pharmacological interventions. Additionally, the elucidation of the regulatory mechanism of URAT1 could lead to the development of new therapeutic agents that target URAT1 phosphorylation. However, our study also has some limitations. Because the UKBB data predominantly included individuals of European ancestry, further evaluation is needed to determine whether our findings apply to other populations with different backgrounds. Owing to the substantial differences in renal urate handling, biochemical analysis has been limited to several human cell lines and human kidneys. The establishment of a humanized knockin *SLC22A12* mouse model, along with uricase inactivation, may enable the in vivo demonstration of the proposed mechanism. However, we confirmed the causal role of *SLC22A12* in our population genetic studies, which highlights the importance of human resources to better understand urate metabolism in light of substantial species-specific differences.

In summary, we demonstrated the mechanism by which hyperinsulinemia acts through URAT1 to increase serum urate levels and that the gene-environment interaction involving *SLC22A12* influences the positive association between hyperinsulinemia and hyperuricemia. Further understanding of how genetic makeup alters the influence of dietary habits can pave the way for personalized medicine to prevent, diagnose, and treat hyperuricemia and other noncommunicable disorders.

## Methods

### Sex as a biological variable.

Our human study involved both male and female subjects and considered sex as a biological variable.

### Cell culture.

Human embryonic kidney (HEK) cells (ATCC) were incubated in DMEM supplemented with 10% FBS and antibiotics as described previously (70). HK-2 cells (ATCC) were propagated in DMEM supplemented with 10% FBS. The expression plasmid encoding full-length hURAT1 (6, 71) with an N-terminal Flag tag in pcDNA3.1 (provided by Naohiko Anzai) was introduced into HEK cells using a nonliposomal polymer (TransIT-X2; Mirus Bio). After overnight serum starvation, insulin (Thermo Fisher Scientific) was added at 1 nM and 100 nM, NaCl was added at 75 mM, and the cells were incubated for 3 hours. The concentrations of insulin and NaCl were determined in accordance with previous studies (28, 43).

### IP.

Flag-tagged hURAT1 expressed in HEK cells was lysed with TNE buffer (10 mM Tris, pH 7.8, 150 mM NaCl, and 1% Triton X) containing cOmplete Protease Inhibitor Cocktail (Roche) and Phosphatase Inhibitor Cocktail 3 (Sigma-Aldrich). Flag-hURAT1 was purified by IP using anti-FLAG M2 magnetic beads (Millipore, catalog M8823) and SureBeads Magnetic Rack (Bio-Rad). Endogenous hURAT1 was purified from HK-2 cells by immunoprecipitation using a specific URAT1 antibody developed in our laboratory (28), followed by incubation with protein A agarose beads.

### Western blotting.

Western blotting was performed as previously described (72). After measuring the protein concentration using the Pierce 660 nm Protein Assay, equal amounts of protein were mixed with Laemmli sample buffer, incubated at room temperature for 20 minutes for membrane proteins, separated on a polyacrylamide gel, and transferred to a nitrocellulose membrane. The membrane was incubated with primary and peroxidase-conjugated secondary antibodies, followed by imaging using the ECL Plus Reagent (PerkinElmer). Tubulin (for total proteins) and cadherin (for cell-surface proteins) were used as endogenous controls. Human kidney lysates were obtained from GeneTex. The antibodies used in this study include anti-FLAG (anti-DDDDK-tag mAb-HRP-DirecT, MBL, catalog M185-7), anti–R-x-x-S^P^/T^P^ antibody (Cell Signaling Technology, catalog 9614), anti-tubulin (Sigma-Aldrich, catalog T6074), anti-cadherin (Sigma-Aldrich, catalog C1821), anti-URAT1 (developed in our lab) (28), anti-phospho-AKT (S473) (Cell Signaling Technology, catalog 9271), AKT (Cell Signaling Technology, catalog 4691), and SGK1 (Cell Signaling Technology, catalog 12103).

### Urate transport.

HEK cells expressing no hURAT1, hURAT1^WT^, or hURAT1^T408A^ were seeded in 24-well tissue culture plates coated with Easy iMatrix-511 (Nippi) at a density of 1.6 × 10^5^ cells/well. After 48 hours, the cells were washed thrice with serum-free HBSS (Thermo Fisher Scientific). The cells were then incubated in a solution containing 10 μmol/L [^14^C]urate (Moravek; MC-1394) at 37°C for 30 minutes. Uptake was stopped by adding ice-cold HBSS. The cells were washed thrice with HBSS and lysed with 0.2 mL of 0.1 N sodium hydroxide and 2.0 mL of Ultima Gold (PerkinElmer). The radioactivity was determined using a liquid scintillation counter (Tri-Carb 3110 TR, PerkinElmer).

### Creation of phospho-specific URAT1 antibody.

To produce antibodies that selectively recognize URAT1 phosphorylated at T408, the peptide C-SHLGRRPT*LAA (*phospho-Thr) was synthesized (Covance). This peptide comprises 401-411 hURAT1 molecules, with cysteine added to the amino-terminus for conjugation. The phosphopeptide was coupled to keyhole limpet hemocyanin (KLH), and rabbits were immunized using the phosphopeptide (Covance). Serum from the immunized rabbits was depleted of nonspecific antibodies with the cognate nonphosphopeptide and specific antibodies purified with the immunizing phosphopeptide. ELISA was used to characterize the purified antibody (Covance). In brief, 96-well microplates were coated with the antigen, phosphorylated peptide, and separately nonphosphorylated peptide (1 μg/mL, 100 μL per well, respectively) overnight. After washing 3 times with PBST (1× PBS + 0.05% Tween 20), plates were blocked with 200 μL of 3% BSA in PBS at room temperature for 1 hour, followed by another 3 washes with PBST. Subsequently, 75 μL of serially diluted antibodies (starting dilution 1:100, diluted by a factor of 10) were added and incubated for 1 hour, washed 6 times with PBST, and incubated with 100 μL of donkey anti-rabbit antibody (diluted 1:5,000 in 3% BSA) for 1 hour. After washing 6 times with PBST, 100 μL/well of 2,2′-azino-bis(3ethylbenzothiazoline-6-sulfonic acid) (ABTS) peroxidase substrate was added and incubated for 30 minutes. The absorbance was measured at 415 and 570 nm using a microplate reader. The specificity of the antibody was further confirmed by dot blot assay and mutagenesis as described in the manuscript.

### Homology modeling.

All procedures were performed using the Homology Modeling Professional for the HyperChem (HMHC) program (73). The cryo-EM structure of OAT1 (Protein Data Bank ID: 8SDU) (74, 75) was used as the template. Calculations were performed using the AMBER99 force field (https://ambermd.org/). Arg, His, and Lys residues were treated as cations and Asp and Glu residues were treated as anions. The side-chain rotamers of amino acid residues that were different from the template (227 residues, excluding glycine, alanine, and proline, which do not have side-chain rotamers) were modeled based on energy calculations using the rotamer database equipped with HMHC. The model was subjected to simulated annealing using molecular dynamics calculations under distance-restraint conditions. After removing the restraint conditions, the overall structure was optimized to obtain the final structure. The precision of the final structure was confirmed using the Ramachandran plot program for HMHC.

### Mutagenesis.

The QuikChange Site-Directed Mutagenesis Lightning Kit (Stratagene) was used to introduce mutations. The sense primers used to introduce mutagenesis were 5′-GGACTGCGCTTCCGGGCATGTATCTCCACGTTGTG-3′ (for T350A), 5′-CTGAGCCACCTGGGCTGCCGCCCCACGCTGGCCGC-3′ (for R405C), 5′-CTGAGCCACCTGGGCGGCCGCCCCACGCTGGCCGC-3′ (for R405G), 5′-CCTGGGCCGCCGCCCCGCACTGGCCGCATCCCTG-3′ (for T408A), and 5′-CCTGGGCCGCCGCCCCATGCTGGCCGCATCCCTG-3′ (for T408M).

### ADP-Glo assay.

The purified URAT1 (WT and T350A/T408A) was incubated with human recombinant AKT1 (SignalChem) in Kinase Dilution Buffer V (SignalChem) and Ultra Pure ATP (Promega) at 37°C for 1 hour. After incubation with ADP-Glo Reagent (Promega) for 40 minutes, Kinase Detection Reagent (Promega) was added and incubated for 1 hour. Luminescence was measured using GloMax 20/20 Luminometer (Promega).

### Cell -surface biotinylation.

Cell-surface biotinylation was performed using the Cell Surface Protein Biotinylation and Isolation Kit (Thermo Fisher Scientific) as described previously (76). Cells grown on 10 cm culture dishes were washed with PBS and incubated with EZ-LINK Sulfo-NHS-SS-biotin (Thermo Fisher Scientific) for 30 minutes at 4°C, followed by the addition of a quenching solution. Cells were lysed with lysis buffer containing the Halt Protease Inhibitor Cocktail Kit (Thermo Fisher Scientific). An aliquot of the lysate was used to detect total protein abundance using Western blot analysis. Biotinylated URAT1 was isolated with a NeutrAvidin agarose gel (Thermo Fisher Scientific), eluted with sample buffer containing dithiothreitol, and used for Western blot analysis.

### Deglycosylation experiments.

For protein deglycosylation, immunopurified URAT1 was incubated with PNGase F (New England Biolabs) in 50 mM sodium phosphate buffer overnight at 37°C (76). The samples were then analyzed by Western blotting, as described above.

### Immunofluorescence study.

HEK cells grown on culture slides (Falcon) were transfected with FLAG-URAT1, fixed with 4% paraformaldehyde in PBS, and permeabilized with 0.1% Triton X-100. The cells were then stained with a rabbit polyclonal antibody against FLAG, followed by an Alexa Fluor 488–conjugated donkey anti-rabbit secondary antibody (Thermo Fisher Scientific). Nuclei were stained with DAPI (Vector). Images were captured using confocal microscopy (LSM880; Zeiss). Human kidney sections were prepared from kidney biopsy samples. Adjacent kidney tissue sections were stained with phospho-specific URAT1 antibody (1:100) and total URAT1 antibody, respectively, followed by anti-rabbit antibody conjugated to the Alexa Fluor 488 fluorophore (Thermo, catalog A-21206).

### Single-cell RNA-Seq analysis.

Information on the individual transcriptome data of the 20 living donor kidneys was obtained from the NIDDK KPMP repository (https://atlas.kpmp.org/repository/). The single-cell RNA-Seq dataset (file name: 521c5b34-3dd0-4871-8064-61d3e3f1775a_PREMIERE_Alldatasets_08132021.h5Seurat) is publicly available as an h5 Seurat object, with quality control and clustering performed by the KPMP team. We reanalyzed this dataset using Seurat version 5.0.1 in RStudio version 4.3.0 (R Development Core Team, Vienna, Austria). The cells were clustered by cell type and visualized using Uniform Manifold Approximation and Projection (UMAP) with the DimPlot function. The expression of SGK1, PRKACA, and PRKG1 were visualized using the DotPlot function and mapped onto the UMAP plot using the FeaturePlot function. The percentage of cells expressing each gene in proximal tubule clusters was also calculated.

### Kinase screen assay.

We synthesized a hURAT1 peptide containing T408 (CSHLGRRPTLAA; the underline indicates T408) and a hURAT1 peptide with a T408A substitution (CSHLGRRPALAA). Profiling of 2 peptide substrates against 53 recombinant serine/threonine kinases was performed at Kinexus. These peptides were incubated with each of the recombinant kinases in triplicate and profiled using the ADP-Glo assay as described above. For AKT1 and SGK1, a hURAT1 peptide with an R405A substitution (CSHLGARPTLAA), which lacks the R-x-x-T^P^ motif, was synthesized at Kinexus. WT hURAT1, T408A-hURAT1, and R405A-hURAT1 peptides were separately incubated with AKT1 and SGK1 in triplicate, and the phosphorylation signal was determined using the ADP-Glo assay.

### Patients and data collection.

We retrospectively extracted a total of 162 outpatients who were 18 years of age or older who visited the Teikyo University Hospital between January 2019 and June 2023 and met the following criteria: (a) serum and urinary urate concentrations, plasma glucose, and insulin levels were measured simultaneously, and (b) urate-lowering drugs and insulin preparations were not used. We excluded patients with eGFRcre levels of less than 45 mL/min/1.73 m^2^, calculated based on the Japanese Society of Nephrology equation (77), as the FEUA is highly influenced by reduced kidney function (78). Demographic (age, sex, height, weight, and medical history) and clinical (serum urate, insulin, plasma glucose, and urinary urate) data were collected from medical records. Glycosuria and proteinuria were determined using a urine dipstick test. HOMA-IR was calculated as plasma glucose (mg/dL) × serum insulin (μU/mL)/405. FEUA, an index of urate excretion, was calculated as FEUA = 100 × [urinary urate (mg/dL) × serum creatinine (mg/dL)]/[serum urate (mg/dL) × urinary creatinine (mg/dL)] and log-transformed for analysis, because the value showed skewed distribution.

### UKBB data analysis.

The UKBB is a large-scale prospective cohort study conducted between 2006 and 2010 in 22 assessment centers across England, Scotland, and Wales, recruiting approximately 500,000 individuals aged 40–69 years from the general population (79). During the baseline assessment, the participants completed a touch-screen questionnaire on lifestyle factors such as diet, smoking, and alcohol consumption. Physical examinations, blood tests, and genotyping assays were performed.

For this study, we included participants from the total UKBB cohort (*n* = 502,416) who had an eGFRcre of 45 mL/min/1.73 m^2^ or more, calculated using the Chronic Kidney Disease Epidemiology Collaboration (CKD-EPI) equation (80), and were not receiving allopurinol or probenecid as urate-lowering medications or any insulin analog agents. We extracted data on serum urate levels (Data-Field 30880), habitual salt intake (assessed by the question “salt added to food” [data-field 1478]), TyG index, a marker of insulin resistance and hyperinsulinemia calculated as Ln (TG [mg/dL] × fasting plasma glucose [mg/dL] /2), where LN = natural logarithm and TG = triglyceride (51, 52, 81), central obesity (assessed by waist-to-hip ratio), blood pressure, HbA1c, total cholesterol, eGFRcre, smoking history, and habitual drinking (days of drinking in a week or month). Individuals for whom this information was unavailable were excluded from analysis. The final study population comprised 377,358 participants. We collected data on physical activity (data field 22040) and fruit intake (calculated by summing the intake of each product obtained from the 24-hour dietary recall questionnaire) (82) from participants with available data. Univariable and multivariable analyses were performed to investigate the associations among salt intake, insulin resistance, and serum urate levels. In the gene-environment interaction analysis, we identified 7 SNPs in the *SLC22A12* gene region that were significantly associated with serum urate levels in the GUGC cohort, which is an independent cohort of approximately 140,000 individuals of European ancestry (4). Among these, genotype information was available for one SNP, rs475688 (C>T), in UKBB, which was used for further analysis. A total of 377,358 participants were divided into 2 groups according to their median TyG index (8.68). Percentage differences in mean serum urate levels, compared with participants with the 0 rs475688 T-allele, were then calculated and plotted for those with 1 and 2 T-alleles in each group. In addition, we extracted 32 nonsynonymous LOF variants in the *SLC22A12* coding region that have been functionally characterized in previous studies (5, 6, 12, 57, 83). Among them, rs14764731511 (11:64367854:G:A[hg19]) was identified as a variant for which the mutation was registered in the UKBB and the imputation accuracy was confirmed (i.e., INFO score ≥ 0.8). Similarly, we screened for SNPs within *SLC2A9* that are associated with serum urate levels and that have an eQTL effect on *SLC2A9* in the human kidney (56).

### Statistics.

In the experimental studies, an unpaired, 2-tailed *t* test was used for comparisons between 2 groups. For multiple comparisons, statistical analysis was performed using ANOVA followed by Dunnett’s post hoc tests. A *P* value of less than 0.05 was considered statistically significant.

Clinical characteristics of participants in the study are summarized as mean ± SD for continuous variables and absolute numbers and percentages for categorical data. Logarithmic transformation was applied to the HOMA-IR and FEUA owing to their skewed distributions. The correlation between these parameters was analyzed using Pearson’s correlation test. Multiple regression analysis was performed to determine the independent association between the HOMA-IR and FEUA levels after adjustment for potential confounders. In Model 1, demographics (age and sex) and HOMA-IR were considered. In addition to these factors, we added smoking and drinking habits as well as BMI in Model 2. In Model 3, we included history of hypertension, HbA1c, total cholesterol, and eGFRcre. As a further test, glycosuria and proteinuria were included as additional covariates in the multiple regression analysis described above.

In the analysis of the UKBB data, insulin resistance (assessed by the TyG index because HOMA-IR was not available) and salt intake habits (assessed by the question “Salt added to food”, treated as a categorical variable with 4 levels: never/rarely, sometimes, usually, and always) were used as explanatory variables, and serum urate level was the dependent variable. We constructed 3 multivariable linear regression models to assess the associations between the explanatory variables and serum urate levels. Model 1 included demographic factors (age, sex, and ethnicity), the TyG index, and habitual salt intake. Model 2 was additionally adjusted for lifestyle factors, including smoking and drinking habits and waist-to-hip ratio. Model 3 was further adjusted for mean blood pressure, HbA1c, total cholesterol, and eGFRcre. Further, we included physical activity and fruit intake as covariates and conducted a multiple regression analysis. For genetic analysis, the difference in the regression coefficients between the lower and higher TyG groups was assessed using 2-tailed Welch’s *t* test in simple linear regression models. For multivariable analysis, the rs475688 T-allele number as well as the interaction term between the rs475688 T-allele number and TyG index (given by their product) were introduced into Model 3 (except for habitual salt intake). The same multivariable analysis was conducted to test the interaction between the TyG index and the possession of the rs147647315 risk allele using the interaction term.

The mediation analysis was conducted using the lavaan R package (54). Similar to the multivariable analysis described above, age, sex, ethnicity, smoking history, habitual drinking, waist-to-hip ratio, mean blood pressure, HbA1c, total cholesterol, and eGFRcre were considered as covariates.

We conducted additional interaction analysis to evaluate the possible involvement of *SLC2A9*. The number of rs4529048 risk allele (A allele) and the interaction term between the rs4529048 A-allele number and the TyG index (given by their product) were introduced into Model 3, similar to the analysis of SNP rs475688.

### Study approval.

Human kidney sections were prepared from kidney biopsy samples with the approval of the Institutional Review Board at Teikyo University School of Medicine (19-119-2). Written, informed consent was waived because of the retrospective nature of the analysis. Data analysis of patients of Teikyo University Hospital and the use of kidney biopsy samples were approved by the Ethics Committee of Teikyo University School of Medicine (21-231). The UKBB study was approved by the North West Multi-Centre Research Ethics Committee as a research tissue bank (11/NW/0382; 16/NW/0274), and all participants provided written informed consent. This study was conducted using the UKBB Resource under application number 78657.

### Data availability.

Genotype and phenotype data from the UKBB are available for use upon application to its website (https://www.ukbiobank.ac.uk/enable-your-research/apply-for-access). The GWAS summary statistics of serum urate levels from the GUGC cohort are available online (https://kp4cd.org/node/179). GWAS summary statistics of serum urate levels from the UKBB are available online (http://www.nealelab.is/uk-biobank/). The healthy donor kidney single-cell RNA-Seq data from the NIDDK KPMP repository are publicly available from its website (https://atlas.kpmp.org/repository/?size=n_1000_n&filters%5B0%5D%5Bfield%5D=data_format&filters%5B0%5D%5Bvalues%5D%5B0%5D=h5Seurat&filters%5B0%5D%5Btype%5D=any).

All the original codes have been deposited on GitHub and are publicly available as of the date of publication (https://github.com/wfujiimed; commit ID wfujiimed). Additional information required to reanalyze the data reported in this paper is available from the corresponding authors upon request. Values for all data points in graphs are reported in the Supporting Data Values file.

## Author contributions

YK and SS designed and supervised the project. WF, OY, DH, KK, EKO, MH, and SS performed the experiments. WF, MT, YK, and SS performed the data analysis. WF and SS wrote the original draft. WF, MT, YK, and SS reviewed and edited the manuscript. All the authors critically revised the draft and approved the final manuscript.

## Supplementary Material

Supplemental data

ICMJE disclosure forms

Unedited blot and gel images

Supporting data values

## Figures and Tables

**Table 2:**
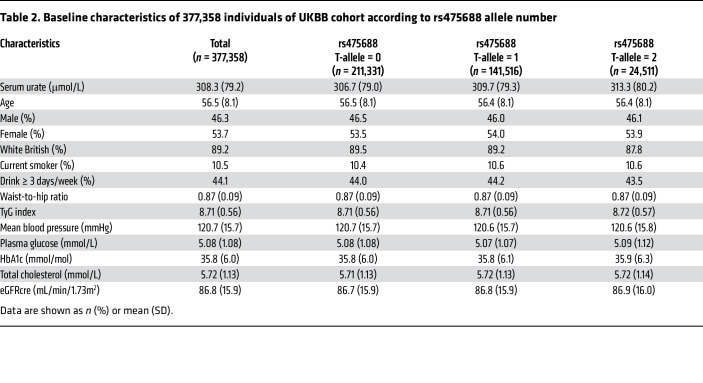
Baseline characteristics of 377,358 individuals of UKBB cohort according to rs475688 allele number

**Table 1:**
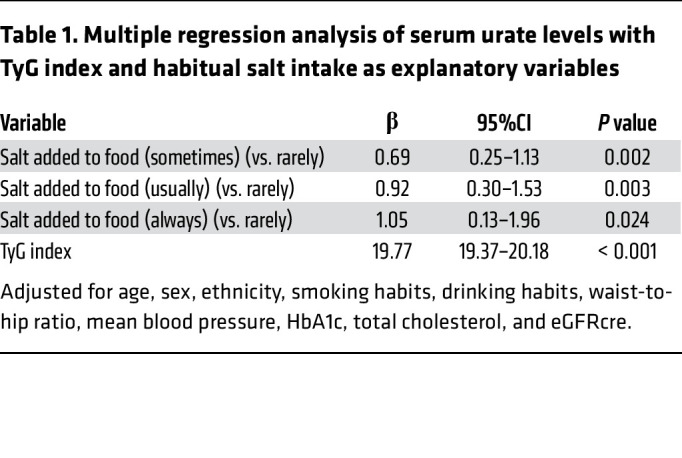
Multiple regression analysis of serum urate levels with TyG index and habitual salt intake as explanatory variables

## Version 1 03/18/2025

In-Press Preview

## Version 2 05/15/2025

Electronic publication
