# Supplementary material for: Gene-environment interaction modifies the association between hyperinsulinemia and serum urate levels through SLC22A12
Source: J Clin Invest. 2025 Mar 18;135(10):e186633. doi: 10.1172/JCI186633 (PMC12077893; doi:10.1172/JCI186633)
Supplement: Supplemental data [file jci-135-186633-s020.pdf]

# Fujii et al. Supplemental Figures

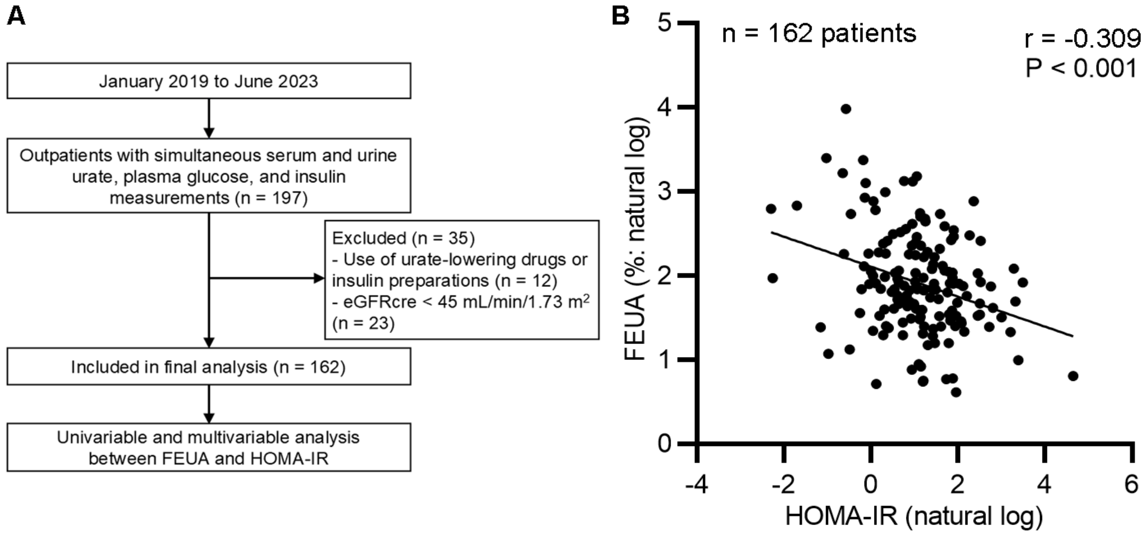


**Supplemental Figure S1.**

Association between hyperinsulinemia and decreased urate excretion. (**A**) Flowchart of patient selection in Teikyo University Hospital. (**B**) Correlation analysis between Homeostatic Model Assessment for Insulin Resistance (HOMA-IR, a maker of insulin resistance and hyperinsulinemia) and fractional excretion of urate (FEUA) in 162 patients.

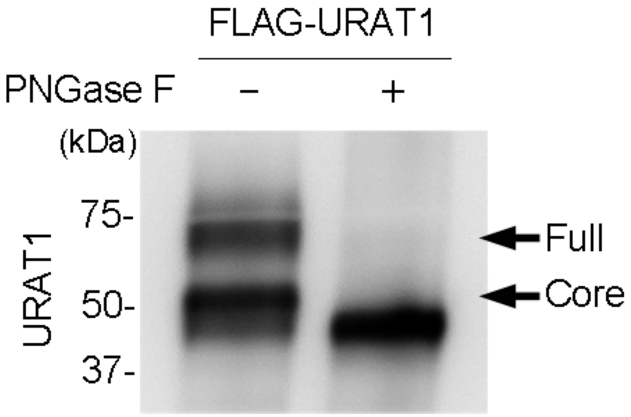


**Supplemental Figure S2.**

Deglycosylation assay of hURAT1. Flag-tagged, human URAT1 (hURAT1) was expressed in HEK-293 cells and purified by Flag-immunoprecipitation (IP). After incubation in the presence and absence of PNGase F, samples were subjected to Western blotting with anti-Flag antibody. The molecular shift of 65-kDa and 50-kDa bands indicates that both signals represent glycosylated form of hURAT1.

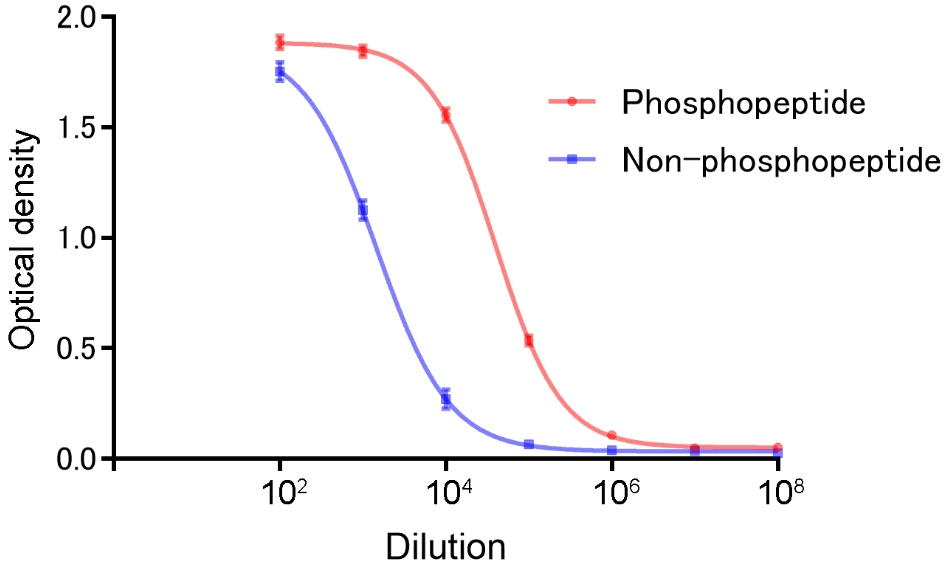


**Supplemental Figure S3.**

Enzyme-linked immunosorbent assay (ELISA) to determine the specificity of antibody against hURAT1 phosphorylated at T408.

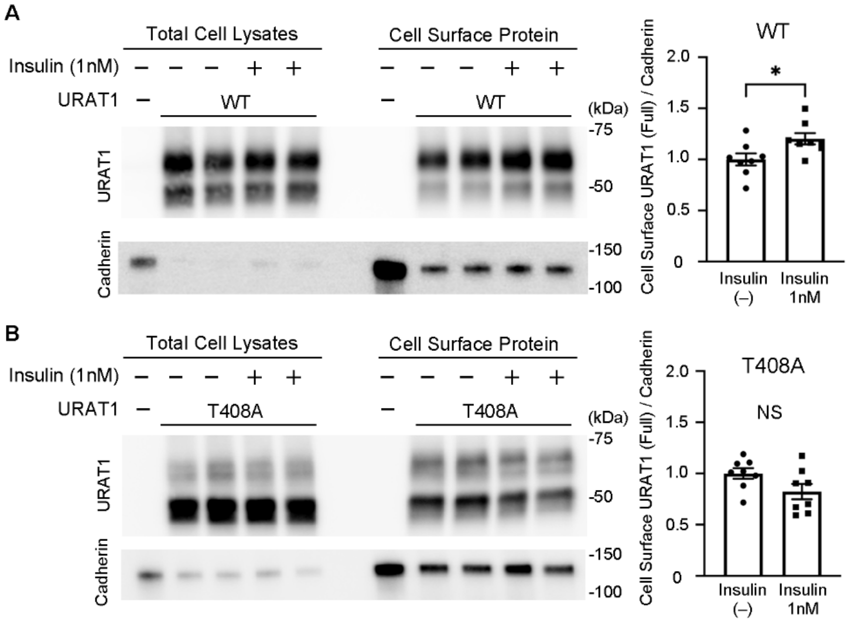


**Supplemental Figure S4.**

Effects of insulin at 1 nM on hURAT1 cell surface abundance in HEK cells expressing $hURAT1^{WT}$ and $hURAT1^{T408A}$. (**A** and **B**) Wild-type (**A**) and non-phosphorylatable T408A (**B**) hURAT1 were expressed in HEK cells and were incubated with insulin at 1 nM for 3 h. Cell-surface levels of hURAT1 were determined by cell-surface biotinylation assay followed by Western blotting. Bar graphs show the results of quantitation. Data are mean ± SEM (n = 8). Statistical analysis: (**A** and **B**) Unpaired t-test. *p < 0.05. NS, not significant.

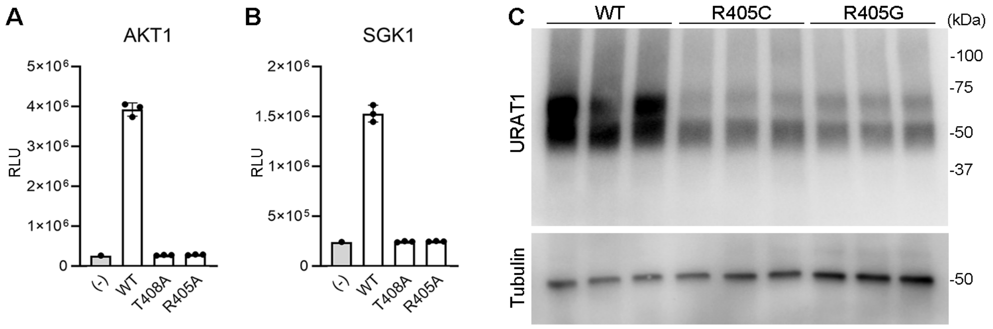


**Supplemental Figure S5.**

Arg at −3 position is necessary for Thr408 phosphorylation and maturation of hURAT1. (**A**) Human URAT1 peptide containing Thr408, hURAT1-T408A peptide, and hURAT1-R405A peptide (which lacks an Arg at −3 position) were synthesized and separately incubated with AKT1 in triplicate. Phosphorylation signal was detected by ADP-glo assay. Assays with hURAT1-T408A peptide and without substrate peptide served as negative controls. Phosphorylation of hURAT1 by AKT1 is abolished by R405A substitution. (**B**) Synthesized peptides described in (**A**) were incubated with SGK1 in triplicate, and phosphorylation signal was analyzed by ADP-Glo assay in triplicate. Phosphorylation of hURAT1 by SGK1 is abolished by R405A substitution. (**C**) Comparison of hURAT1 maturation among wild-type hURAT1 and nonsynonymous single-nucleotide variants (rs563239942; hURAT1-R405C and hURAT1-R405G).

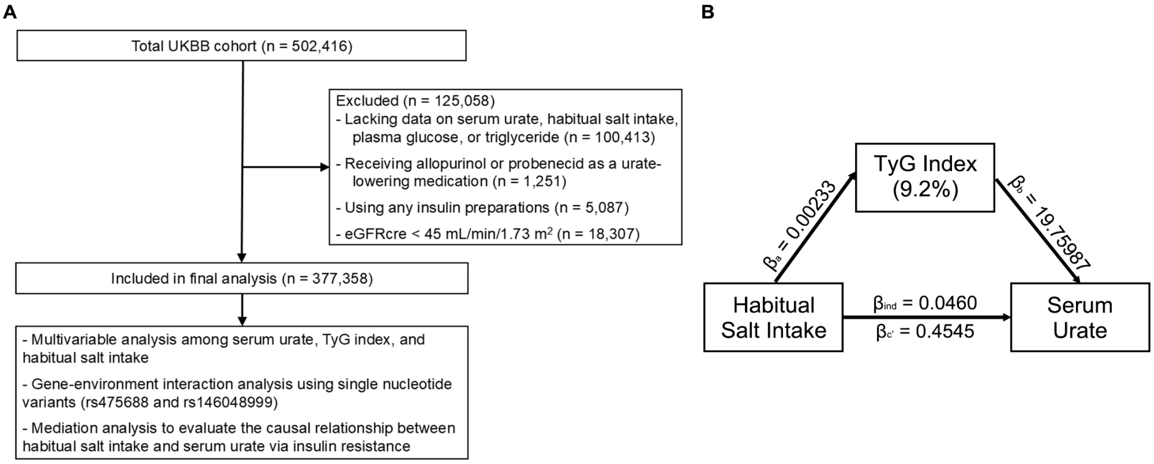


**Supplemental Figure S6.**

Flowchart showing the participant selection and mediation analysis among serum urate, TyG index, and habitual salt intake in the UK biobank (UKBB) study.

(**A**) A flowchart of participant selection. (**B**) Mediation analysis on the relationship among habitual salt intake, TyG index, and serum urate levels. Whether insulin resistance, as measured by the TyG index, mediates the association between habitual salt intake and serum urate levels described in Table 2 was analyzed by mediation analysis (see Methods). $\beta_a$ represents the effect of habitual salt intake on the mediator (TyG index); $\beta_b$ indicates the effect of the mediator on serum urate levels; $\beta_{c'}$ denotes the direct effect of habitual salt intake on serum urate levels. The indirect effect *via* the TyG index ($\beta_{ind}$) was calculated as $\beta_a \times \beta_b$, and its proportion relative to the total effect ($\beta_{c'} + \beta_a \times \beta_b$) was quantified. See also Supplemental Table S8.

Table S1. Baseline characteristics of 162 patients according to HOMA-IR.

| **Characteristics** | **Total (n = 162)** | **HOMA - IR < 2.5 (n = 64)** | **HOMA - IR ≥ 2.5 (n = 98)** | ***P* value** |
|---|---|---|---|---|
| Serum Urate (μmol/L) | 327.5 (107.7) | 305.4 (101.3) | 342.0 (109.8) | 0.034 |
| FEUA (%) | 8.24 (6.38) | 9.99 (8.45) | 7.10 (4.24) | 0.005 |
| Age | 61.9 (16.2) | 63.8 (16.5) | 60.7 (16.0) | 0.229 |
| Male | 100 (62%) | 36 (56%) | 64 (65%) | 0.320 |
| Current Smoker | 36 (22%) | 12 (19%) | 24 (25%) | 0.506 |
| Drink ≥ 3 Days/week | 44 (27%) | 16 (25%) | 28 (29%) | 0.750 |
| Hypertension | 91 (56%) | 35 (55%) | 56 (57%) | 0.884 |
| Diabetes Mellitus | 107 (66%) | 32 (50%) | 75 (77%) | 0.001 |
| Hyperlipidemia | 72 (44%) | 25 (39%) | 47 (48%) | 0.341 |
| BMI (kg/m$^2$) | 24.5 (5.4) | 22.1 (4.7) | 26.0 (5.3) | < 0.001 |
| Plasma Glucose (mmol/L) | 8.81 (5.02) | 6.40 (2.37) | 10.38 (5.64) | < 0.001 |
| HbA1c (mmol/mol) | 64.6 (29.9) | 57.2 (25.3) | 69.4 (31.7) | 0.011 |
| Total Cholesterol (mmol/L) | 5.11 (1.69) | 4.85 (1.11) | 5.26 (1.95) | 0.147 |
| eGFRcre (mL/min/1.73m$^2$) | 74.5 (24.7) | 76.3 (27.0) | 73.4 (23.1) | 0.479 |
| Glycosuria | 57 (35%) | 13 (20%) | 44 (45%) | 0.002 |
| Proteinuria | 36 (22%) | 15 (23%) | 21 (21%) | 0.914 |

FEUA, fractional excretion of urate; BMI, body mass index; HbA1c, hemoglobin A1c;

eGFRcre, estimated glomerular filtration rate calculated by creatinine;

HOMA-IR, homeostatic model assessment for insulin resistance.

Data are shown as n (%) or mean (SD).

Table S2. Multiple regression analysis of FEUA (natural log) with HOMA-IR as explanatory variable.

| Variable | β | 95%CI | *P* value |
|---|---|---|---|
| BMI | -0.021 | -0.041 − -0.002 | 0.028 |
| HOMA-IR (natural log) | -0.106 | -0.203 − -0.010 | 0.031 |
| Age, yrs | 0.008 | 0.001 − 0.014 | 0.016 |
| Male (vs. female) | -0.080 | -0.260 to 0.100 | 0.382 |
| Current Smoker | 0.211 | -0.003 to 0.426 | 0.054 |
| Drink ≥ 3 Days/week | -0.162 | -0.356 to 0.032 | 0.100 |
| Hypertension | 0.062 | -0.149 to 0.273 | 0.564 |
| Diabetes Mellitus | -0.005 | -0.208 to 0.198 | 0.962 |
| Hyperlipidemia | 0.008 | -0.192 to 0.209 | 0.934 |
| eGFRcre (mL/min/1.73$m^2$) | 0.003 | -0.001 to 0.007 | 0.142 |

Table S3. Multiple regression analysis of FEUA (natural log) with HOMA-IR as explanatory variable (glycosuria and proteinuria were included as additional covariates in model described in Table S2).

| Variable | β | 95%CI | *P* value |
|---|---|---|---|
| BMI | -0.020 | -0.040 − -0.001 | 0.038 |
| HOMA-IR (natural log) | -0.110 | -0.210 − -0.010 | 0.031 |
| Age, yrs | 0.007 | 0.001 − 0.014 | 0.035 |
| Male (vs. female) | -0.079 | -0.261 to 0.100 | 0.389 |
| Current Smoker | 0.224 | 0.007 to 0.440 | 0.043 |
| Drink ≥ 3 Days/week | -0.182 | -0.382 to 0.017 | 0.073 |
| Hypertension | 0.073 | -0.143 to 0.289 | 0.503 |
| Diabetes Mellitus | 0.011 | -0.216 to 0.238 | 0.925 |
| Hyperlipidemia | -0.004 | -0.207 to 0.200 | 0.972 |
| eGFRcre (mL/min/1.73$m^2$) | 0.002 | -0.002 to 0.007 | 0.288 |
| Glycosuria | -0.013 | -0.238 to 0.212 | 0.911 |
| Proteinuria | -0.121 | -0.348 to 0.106 | 0.294 |

Table S4. Kinase screening assay of 53 AGC kinases.

| Kinase | Signal Ratio (WT vs T408A) |
|---|---|
| AKT1 | 35.9740079 |
| AKT2 | 3.32342976 |
| AKT3 | 26.28964623 |
| CDC42BPG | 4.821177914 |
| DMPK | 1.08980649 |
| GRK1 | 1.055577285 |
| GRK2 | 1.033572491 |
| GRK3 | 1.031960293 |
| GRK5 | 1.072406516 |
| GRK6 | 1.026160795 |
| GRK7 | 1.034224972 |
| LATS1 | 1.228488103 |
| LATS2 | 1.047736067 |
| MAST3 | 1.084295917 |
| MASTL | 1.064729878 |
| MRCKalpha | 5.290781272 |
| MRCKbeta | 4.305585278 |
| MSK1 | 8.38225172 |
| MSK2 | 1.481949101 |
| NDR | 1.191687683 |
| NDR2(STK38L) | 1.250226176 |
| p70S6K | 1.103142685 |
| p70S6Kb | 1.383486065 |
| PKAc-alpha | 48.67296296 |
| PKAc-beta | 19.066839 |
| PKAc-gamma | 3.354892549 |
| PKCalpha | 3.318294075 |
| PKCbetaI | 8.116876246 |
| PKCbetaII | 3.647305855 |
| PKCdelta | 1.70033364 |
| PKCepsilon | 1.334921737 |
| PKCeta | 2.14918051 |
| PKCgamma | 1.368075862 |
| PKCiota | 1.369963881 |
| PKCtheta | 16.62783688 |
| PKCzeta | 2.119428894 |
| PKN1/PRK1 | 4.065815335 |
| PKN2/PRK2 | 2.181856663 |
| PKN3/PRK3 | 1.137933847 |
| PRKG1 | 28.61257712 |
| PRKG2 | 10.4298703 |
| PRKX | 14.64817893 |
| ROCK1 | 3.807741649 |
| ROCK2 | 2.975303438 |
| RSK1 | 2.183700935 |
| RSK2 | 1.636480672 |
| RSK3 | 1.197147071 |
| RSK4 | 2.046223864 |
| SGK1 | 26.80118706 |
| SGK2 | 5.369436074 |
| SGK3 | 4.500632892 |
| STK32B(YANK2) | 1.030386529 |
| STK32C(YANK3) | 1.053317144 |

Table S5. Gene expression of *Slc22a12* in disected rat nephron as analyzed by RNA-seq.

Data are desribed by Lee JW et al (J Am Soc Nephrol 26:2669-2677, 2015; https://esbl.nhlbi.nih.gov/helixweb/Database/NephronRNAseq/All_transcripts.html).

Values indicate median RPKM.

| Gene Symbol | S1 | S2 | S3 | SDL | LDLOM | LDLIM | tAL | mTAL | cTAL | DCT | CNT | CCD | OMCD | IMCD |
|---|---|---|---|---|---|---|---|---|---|---|---|---|---|---|
| *Slc22a12* | 1.6 | 21.6 | 120.5 | 0.0 | 0.0 | 0.0 | 0.0 | 0.0 | 0.1 | 0.0 | 0.2 | 0.0 | 0.0 | 0.0 |

S1, first segment of the proximal tubule; S2, second segment of the proximal tubule; S3, third segment of the proximal tubule; SDL, short descending limb of the loop of Henle; LDLOM, long descending limb of the loop of Henle in the outer medulla; LDLIM, long descending limb of the loop of Henle in the inner medulla; tAL, thin ascending limb of the loop of Henle; mTAL, medullary thick ascending limb of the loop of Henle; cTAL, cortical thick ascending limb of the loop of Henle; DCT, distal convoluted tubule; CNT, connecting tubule; CCD, cortical collecting duct; OMCD, outer medullary collecting duct; IMCD, inner medullary collecting duct.

Table S6. Baseline characteristics of 377,358 individuals of UKBB cohort according to habitual salt intake.

| **Characteristics** | **Overall (n = 377,358)** | **Never/Rarely (n = 209,630)** | **Sometimes (n = 105,915)** | **Usually (n = 43,830)** | **Always (n = 17,983)** |
|---|---|---|---|---|---|
| Serum Urate (μmol/L) | 308.3 (79.2) | 305.1 (78.3) | 310.2 (79.6) | 316.3 (80.4) | 314.9 (82.3) |
| Age | 56.5 (8.1) | 56.5 (8.1) | 56.4 (8.1) | 57.0 (8.0) | 55.9 (8.3) |
| Male (%) | 46.3 | 44.6 | 46.7 | 51.9 | 49.3 |
| White British (%) | 89.2 | 90.9 | 88.3 | 86.9 | 81.6 |
| Current Smoker (%) | 10.5 | 7.9 | 11.3 | 15.3 | 23.9 |
| Drink ≥ 3 Days/week (%) | 44.1 | 42.5 | 45.2 | 49.0 | 43.6 |
| Waist to Hip Ratio | 0.87 (0.09) | 0.87 (0.09) | 0.87 (0.09) | 0.88 (0.09) | 0.89 (0.09) |
| TyG Index | 8.71 (0.56) | 8.68 (0.55) | 8.72 (0.56) | 8.75 (0.57) | 8.76 (0.58) |
| Mean Blood Pressure (mmHg) | 120.7 (15.7) | 120.9 (15.8) | 120.4 (15.6) | 120.5 (15.5) | 119.7 (15.7) |
| Plasma Glucose (mmol/L) | 5.08 (1.08) | 5.07 (1.06) | 5.09 (1.12) | 5.08 (1.07) | 5.09 (1.13) |
| HbA1c (mmol/mol) | 35.8 (6.0) | 35.7 (5.8) | 36.0 (6.3) | 36.0 (6.2) | 36.5 (7.32) |
| Total Cholesterol (mmol/L) | 5.72 (1.13) | 5.69 (1.13) | 5.74 (1.13) | 5.75 (1.14) | 5.72 (1.15) |
| eGFRcre (mL/min/1.73m$^2$) | 86.8 (15.9) | 86.9 (15.8) | 86.7 (16.0) | 86.4 (15.8) | 85.9 (16.2) |
| Physical Activity (MET minutes per week)* | 2696.6 (2613.9) | 2703.3 (2620.5) | 2679.8 (2599.4) | 2711.2 (2640.9) | 2681.8 (2556.3) |
| Fruit Intake (serving per day)** | 3.27 (2.26) | 3.42 (2.27) | 3.14 (2.20) | 2.97 (2.22) | 2.68 (2.38) |

Data are shown as %, or mean (SD).

*Physical activity observations, n = 300,277. **Fruit intake observations, n = 160,981.

Table S7. Additional multiple regression analysis of serum urate levels with TyG index and habitual salt intake.

| **Variable** | **β** | **95%CI** | ***P* value** |
|---|---|---|---|
| Salt Added to Food (Sometimes) (vs. Rarely) | 0.87 | 0.37 − 1.36 | < 0.001 |
| Salt Added to Food (Usually) (vs. Rarely) | 0.78 | 0.09 − 1.47 | 0.026 |
| Salt Added to Food (Always) (vs. Rarely) | 1.37 | 0.35 − 2.39 | 0.008 |
| TyG Index | 19.86 | 19.40 − 20.32 | < 0.001 |

Adjusted for age, sex, ethnicity, smoking habits, drinking habits, waist-to-hip ratio, mean blood pressure, HbA1c, total cholesterol, eGFRcre, and physical acticity.

| **Variable** | **β** | **95%CI** | ***P* value** |
|---|---|---|---|
| Salt Added to Food (Sometimes) (vs. Rarely) | 0.67 | 0.01 − 1.33 | 0.046 |
| Salt Added to Food (Usually) (vs. Rarely) | 0.96 | 0.01 − 1.90 | 0.047 |
| Salt Added to Food (Always) (vs. Rarely) | 2.07 | 0.49 − 3.66 | 0.010 |
| TyG Index | 19.25 | 18.63 − 19.88 | < 0.001 |

Adjusted for age, sex, ethnicity, smoking habits, drinking habits, waist-to-hip ratio, mean blood pressure, HbA1c, total cholesterol, eGFRcre, and fruit intake.

Table S8. Mediation analysis of the effect of habitual salt intake on serum urate levels via insulin resistance.

| Exposure | Mediator | Outcome | Pathway | Estimate | P value | Mediation Effect (%) |
|---|---|---|---|---|---|---|
| Salt Added to Food | TyG Index | Serum Urate | Salt → TyG ($\beta_a$) | 0.00233 | 0.008 | 9.2 |
| | | | TyG → UA ($\beta_b$) | 19.75987 | < 0.001 | |
| | | | Salt → UA ($\beta_{c'}$) | 0.45450 | < 0.001 | |
| | | | Indirect Effect | 0.0460 | 0.008 | |
| | | | Total Effect | 0.5005 | < 0.001 | |

Table S9. Information on candidate URAT1 loss-of-function mutations from previous studies.

| rs number | Site (GRCh37) | Ref | Alt | Mutation Type | AA Change | MAF (%) | INFO Score |
|---|---|---|---|---|---|---|---|
| rs141570522 | 64359252 | T | C | Missense | I75T | 0.057 | 0.503 |
| rs150255373 | 64366298 | C | T | Missense | R325W | 0.084 | 0.757 |
| rs147647315 | 64367854 | G | A | Missense | R434H | 0.063 | 1 |

Ref, reference allele; Alt, alternative allele; AA, amino acid; MAF, minor allele frequency.

Table S10. Baseline characteristics of 377,358 individuals of UKBB cohort according to rs147647315 risk allele.

| **Characteristics** | **Total** (n = 377,358) | **URAT1-R434H mutation (-)** (n = 376,887) | **URAT1-R434H mutation (+)** (n = 471) |
|---|---|---|---|
| Serum Urate (μmol/L) | 308.3 (79.2) | 308.3 (79.2) | 262.6 (70.7) |
| Age | 56.5 (8.1) | 56.5 (8.1) | 54.6 (8.4) |
| Male (%) | 46.3 | 46.3 | 46.5 |
| White British (%) | 89.2 | 89.3 | 49.3 |
| Current Smoker (%) | 10.5 | 10.5 | 10.6 |
| Drink ≥ 3 Days/week (%) | 44.1 | 44.1 | 33.1 |
| Waist to Hip Ratio | 0.87 (0.09) | 0.87 (0.09) | 0.87 (0.08) |
| TyG Index | 8.71 (0.56) | 8.71 (0.56) | 8.59 (0.61) |
| Mean Blood Pressure (mmHg) | 120.7 (15.7) | 120.7 (15.7) | 120.3 (17.1) |
| Plasma Glucose (mmol/L) | 5.08 (1.08) | 5.08 (1.08) | 5.13 (1.56) |
| HbA1c (mmol/mol) | 35.8 (6.0) | 35.8 (6.0) | 37.3 (8.5) |
| Total Cholesterol (mmol/L) | 5.72 (1.13) | 5.72 (1.13) | 5.55 (1.26) |
| eGFRcre (mL/min/1.73m$^2$) | 86.8 (15.9) | 86.8 (15.9) | 89.4 (16.4) |

Table S11. Baseline characteristics of 377,358 individuals of UKBB cohort according to rs4529048 allele number.

| **Characteristics** | **Total (n = 377,358)** | **rs4529048 A allele = 0 (n = 23,922)** | **rs4529048 A allele = 1 (n = 140,551)** | **rs4529048 A allele = 2 (n = 212,885)** |
|---|---|---|---|---|
| Serum Urate (μmol/L) | 308.3 (79.2) | 268.3 (80.4) | 299.4 (78.4) | 318.6 (77.4) |
| Age | 56.5 (8.1) | 56.2 (8.1) | 56.4 (8.1) | 56.5 (8.1) |
| Male (%) | 46.3 | 46.4 | 46.3 | 46.3 |
| White British (%) | 89.2 | 85.4 | 88.4 | 90.2 |
| Current Smoker (%) | 10.5 | 10.6 | 10.4 | 10.5 |
| Drink ≥ 3 Days/week (%) | 44.1 | 42.7 | 43.9 | 44.3 |
| Waist to Hip Ratio | 0.87 (0.09) | 0.87 (0.09) | 0.87 (0.09) | 0.87 (0.09) |
| TyG Index | 8.71 (0.56) | 8.70 (0.56) | 8.71 (0.56) | 8.71 (0.56) |
| Mean Blood Pressure (mmHg) | 120.7 (15.7) | 120.3 (15.7) | 120.6 (15.7) | 120.7 (15.7) |
| Plasma Glucose (mmol/L) | 5.08 (1.08) | 5.07 (1.06) | 5.08 (1.07) | 5.08 (1.09) |
| HbA1c (mmol/mol) | 35.8 (6.0) | 35.8 (5.9) | 35.8 (6.1) | 35.8 (6.0) |
| Total Cholesterol (mmol/L) | 5.72 (1.13) | 5.70 (1.13) | 5.71 (1.13) | 5.72 (1.14) |
| eGFRcre (mL/min/1.73m$^2$) | 86.8 (15.9) | 87.0 (15.9) | 86.9 (15.9) | 86.7 (15.9) |
