# Supplementary material for: Gene-environment interaction modifies the association between hyperinsulinemia and serum urate levels through SLC22A12
Source: J Clin Invest. 2025 Mar 18;135(10):e186633. doi: 10.1172/JCI186633 (PMC12077893; doi:10.1172/JCI186633)

Full unedited blots for Figure 1A

IP: Flag
WB: RxxS/T^P

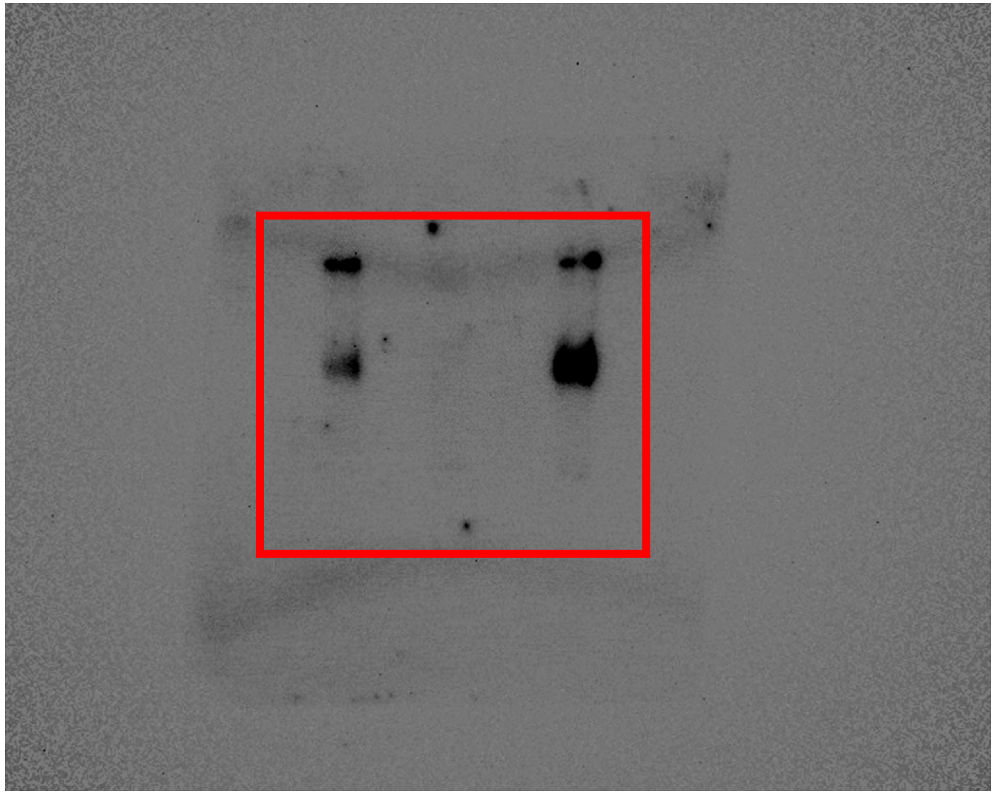

IP: Flag
WB: Flag

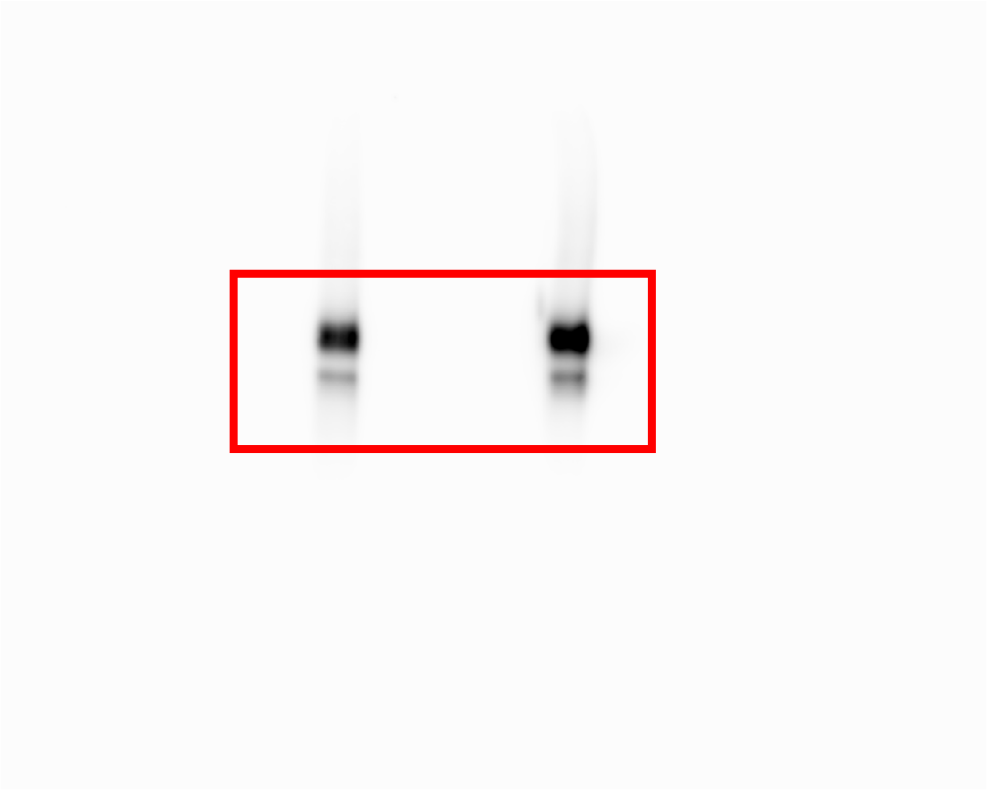

Figure 1E

URAT1

Cadherin

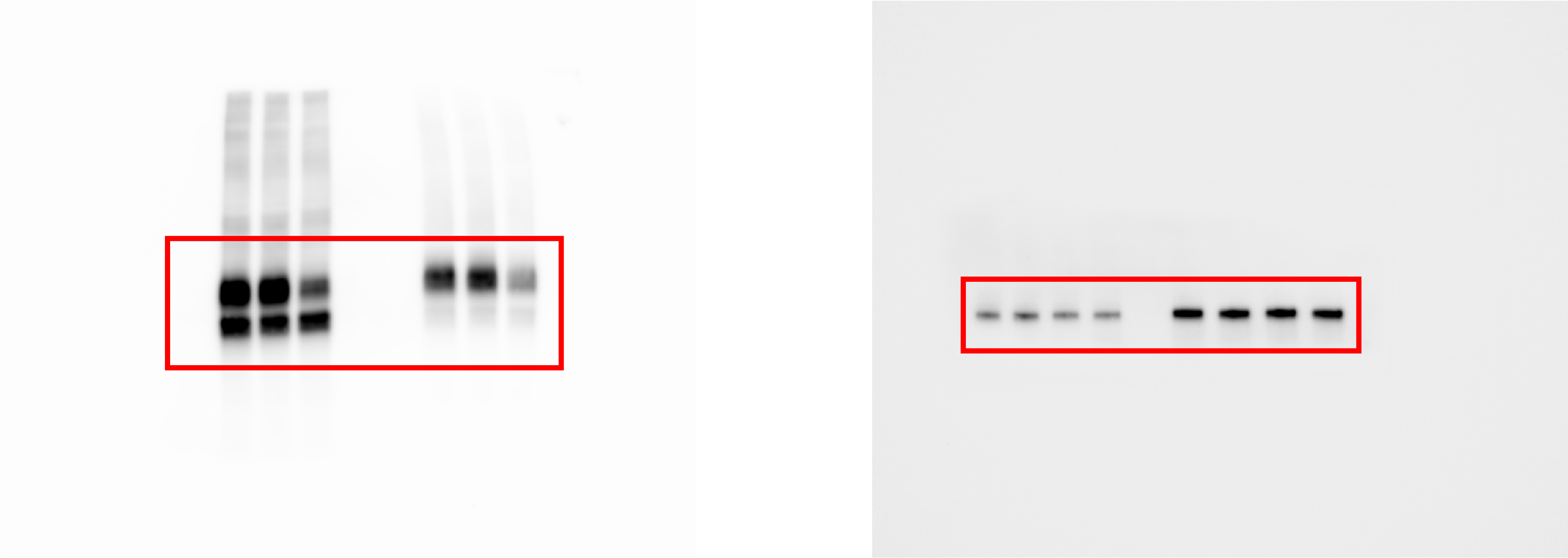

Figure 1E

Tubulin

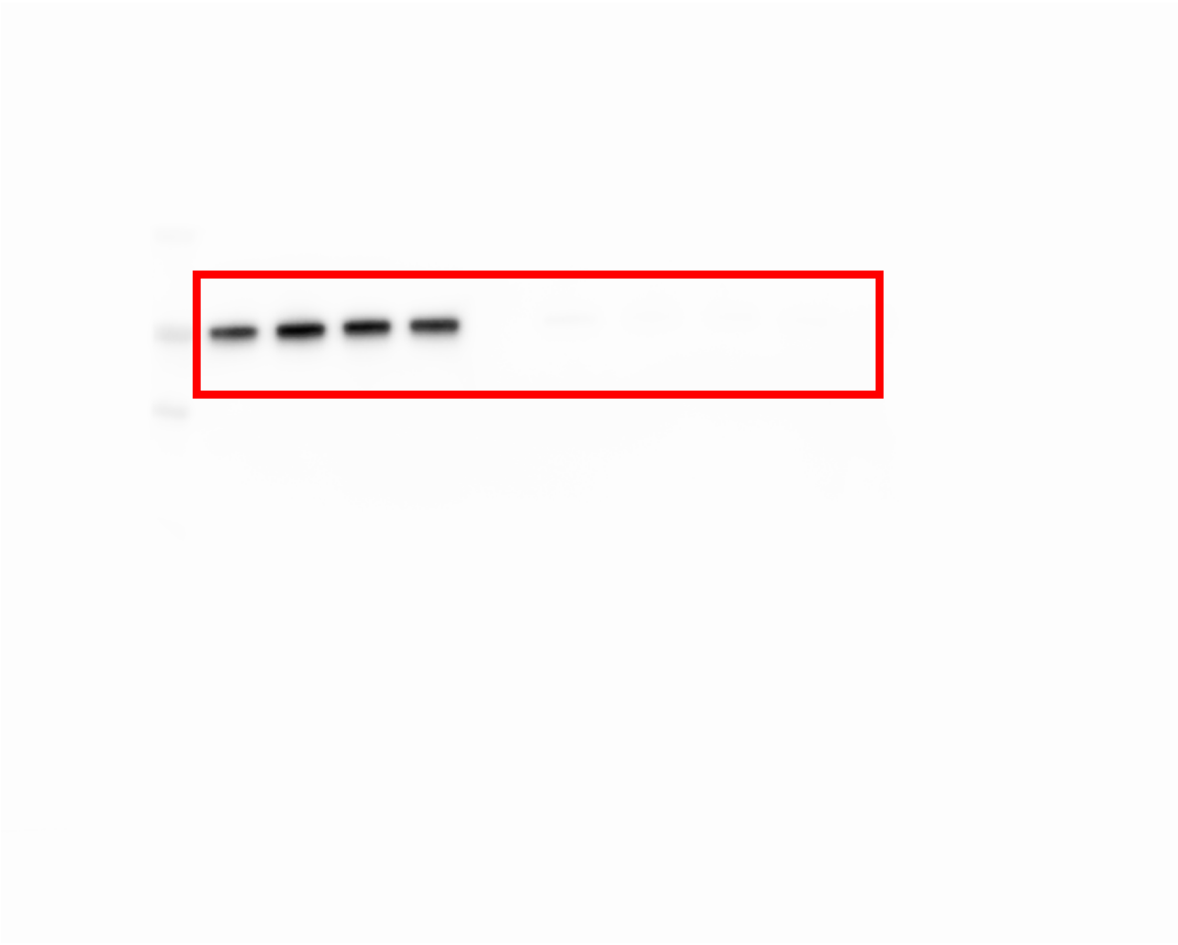

Figure 1H

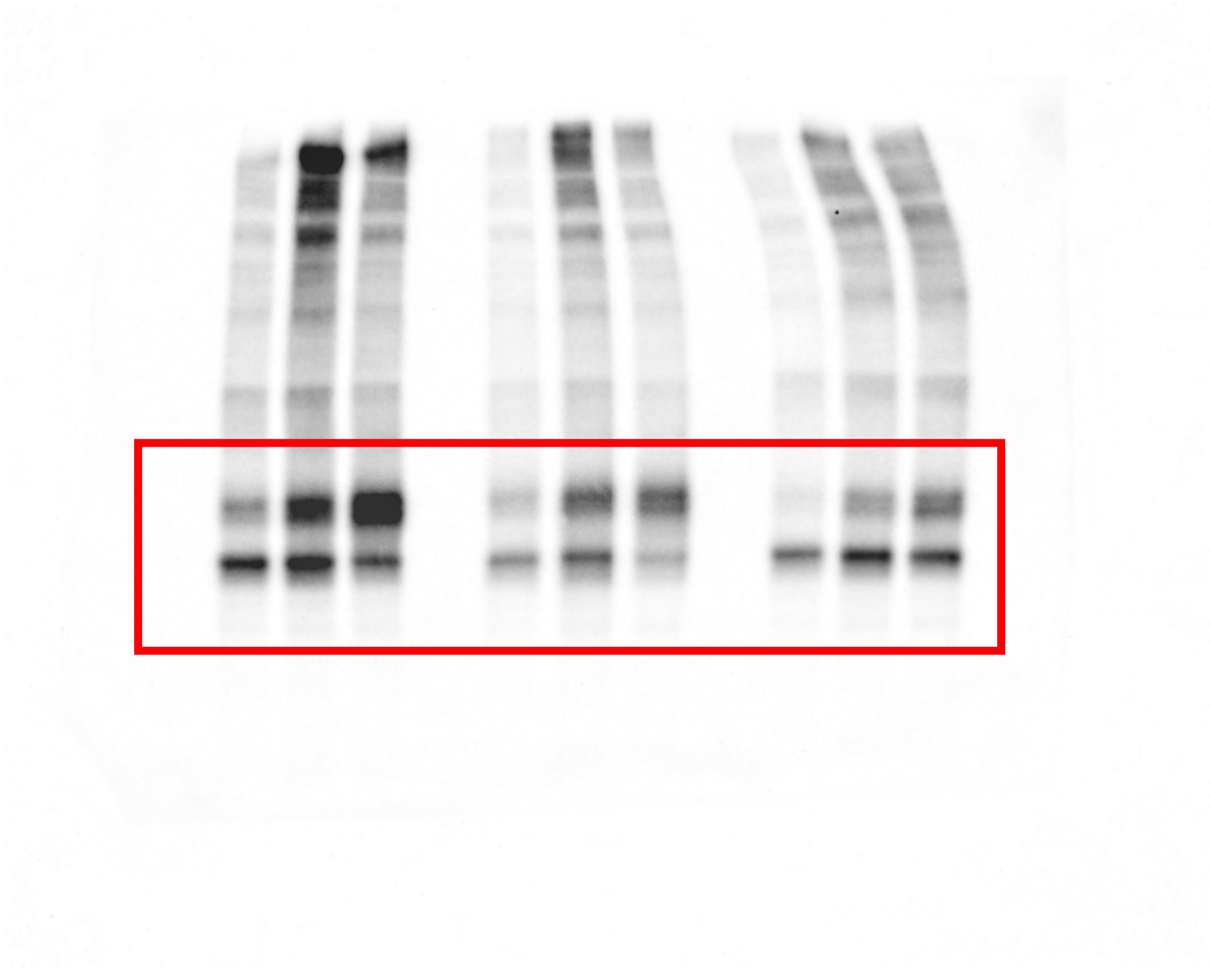

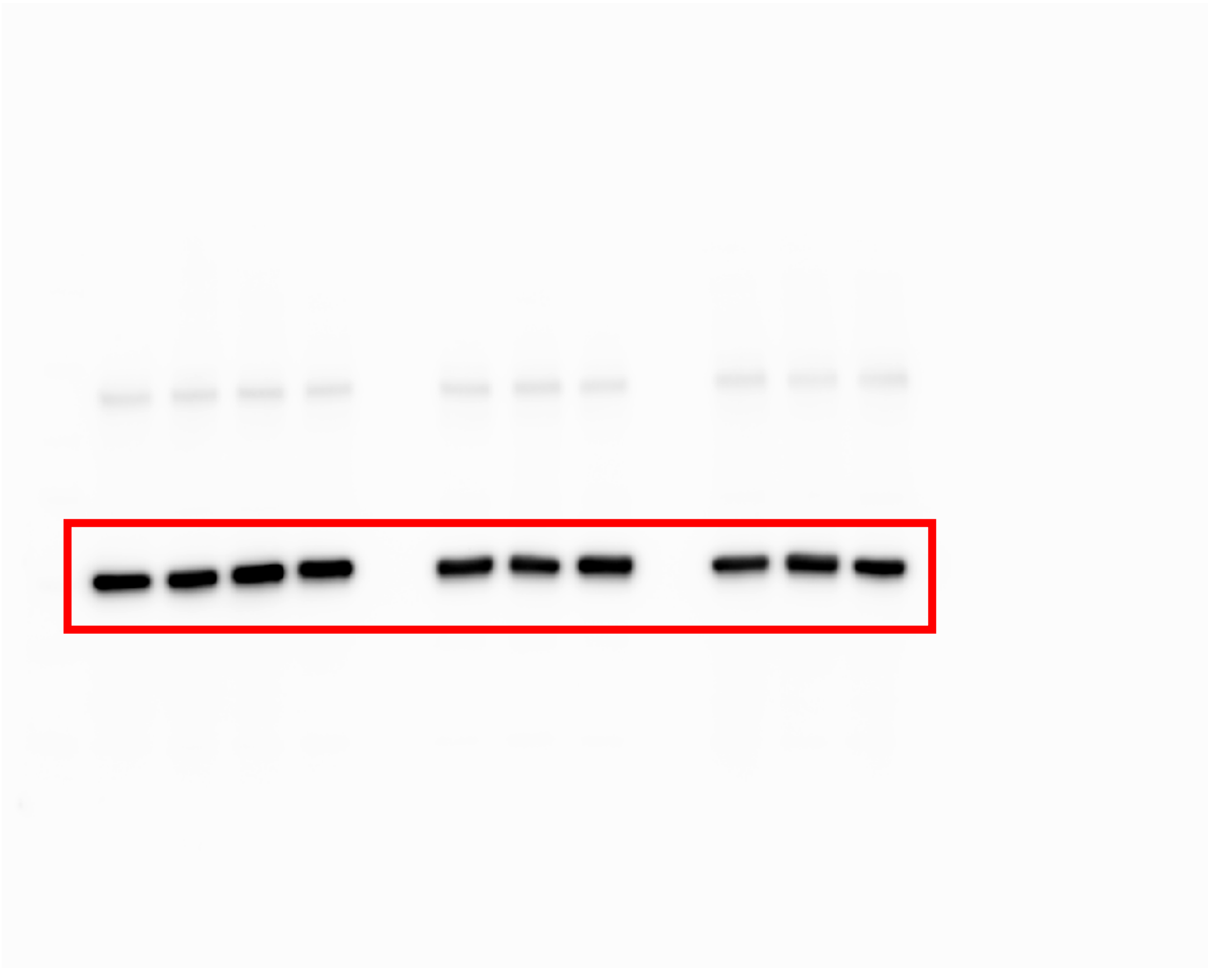

Figure 1I

URAT1

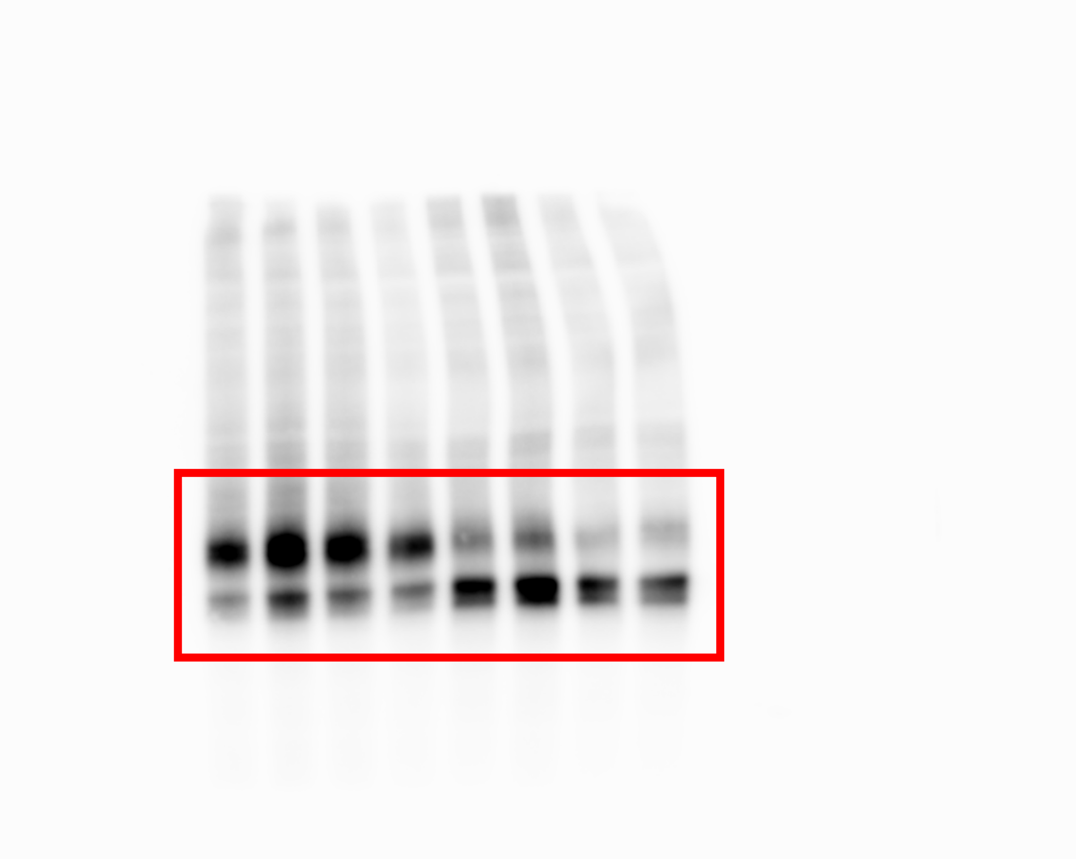

Tubulin

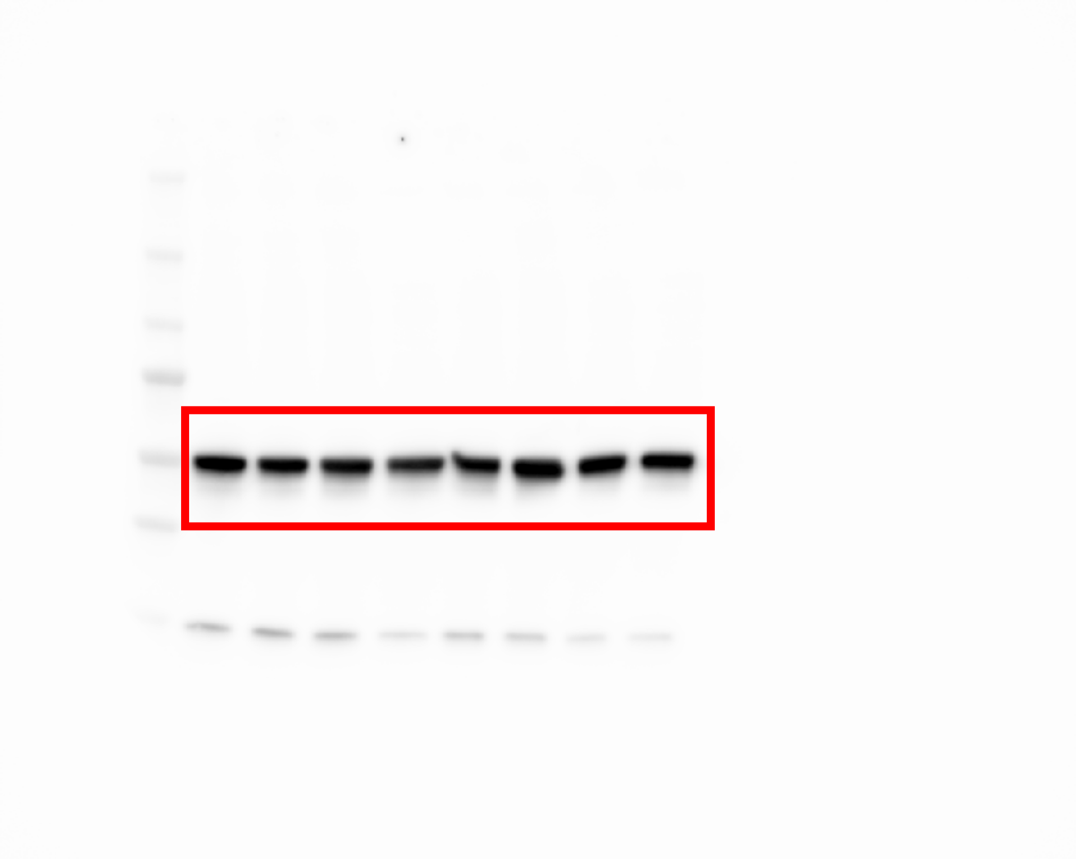

Figure 2A

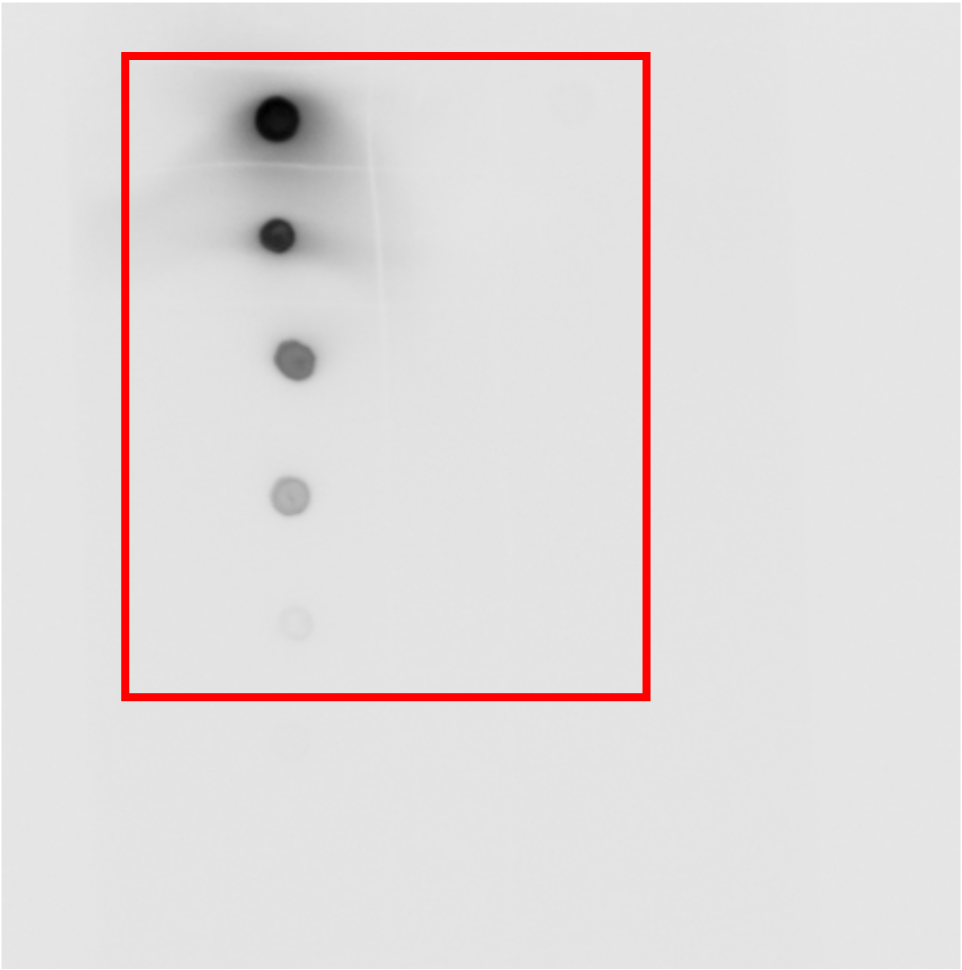

Full unedited blots for Figure 2B

IP: Flag
WB: Phospho-URAT1

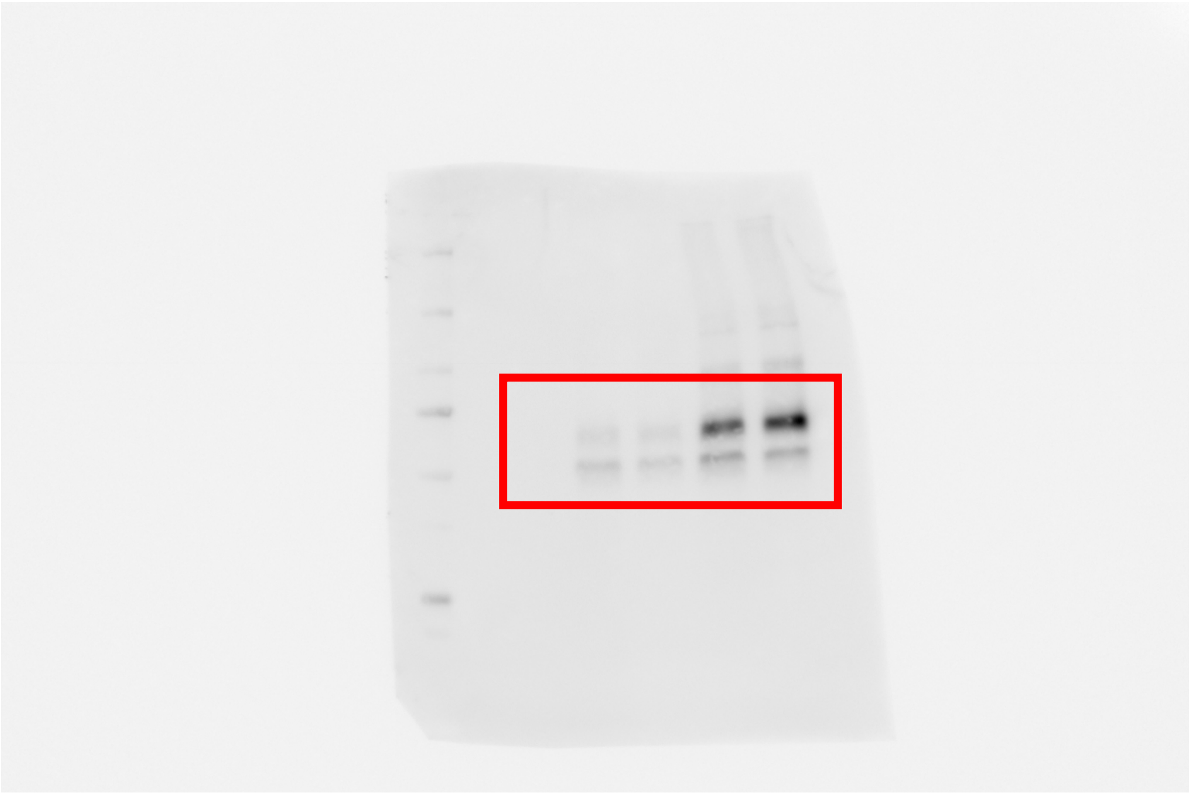

IP: Flag
WB:Flag

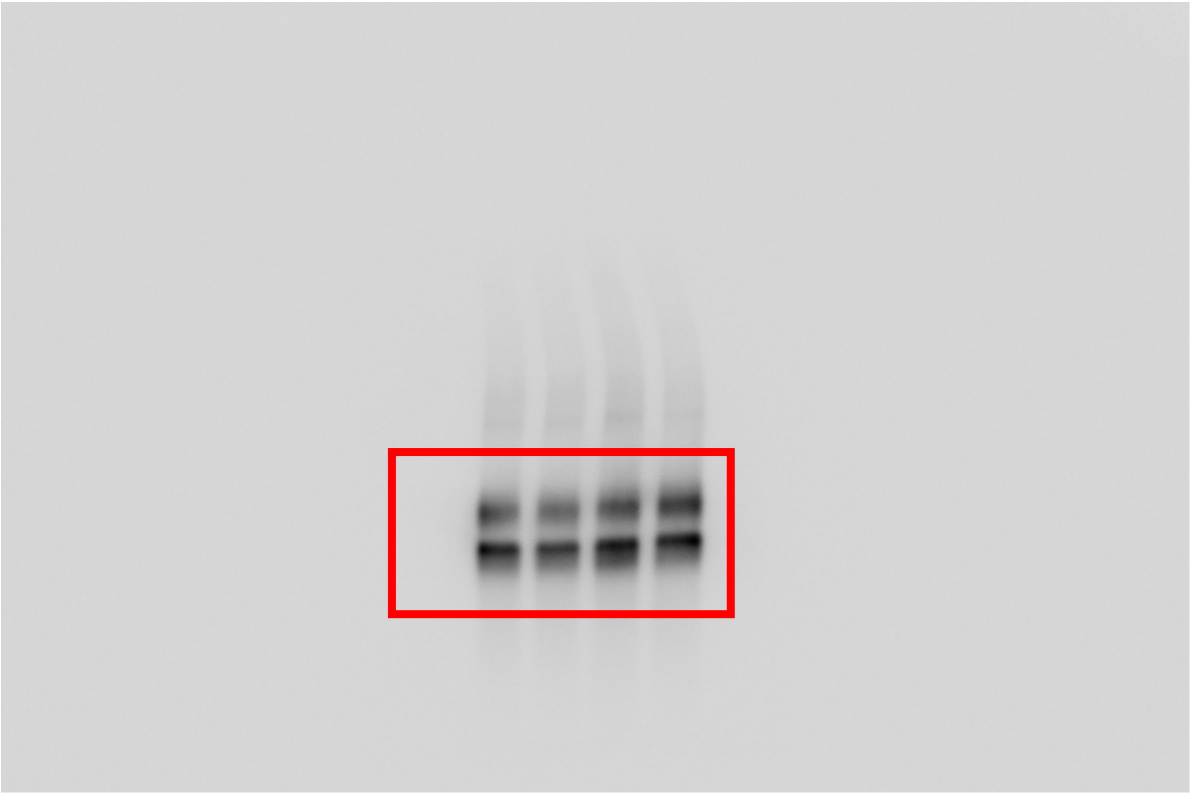

Figure 2C

IP: Flag
WB: Phospho-αURAT1

IP: Flag
WB:Flag

IP: Flag
WB: Phospho-αURAT1

IP: Flag
WB:Flag

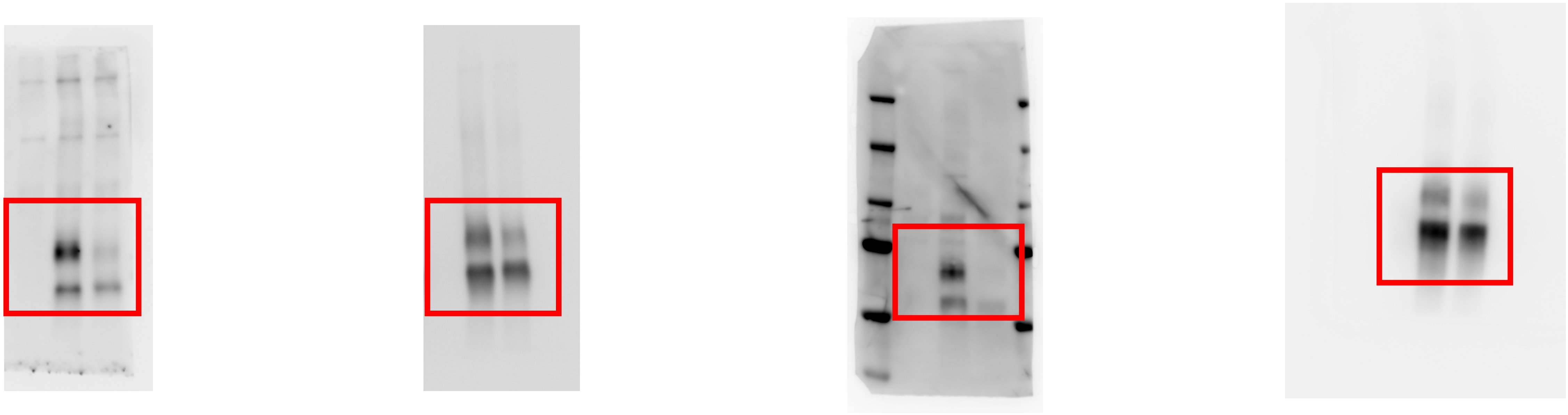

Figure 2D

IP: URAT1
WB: Phospho-αURAT1

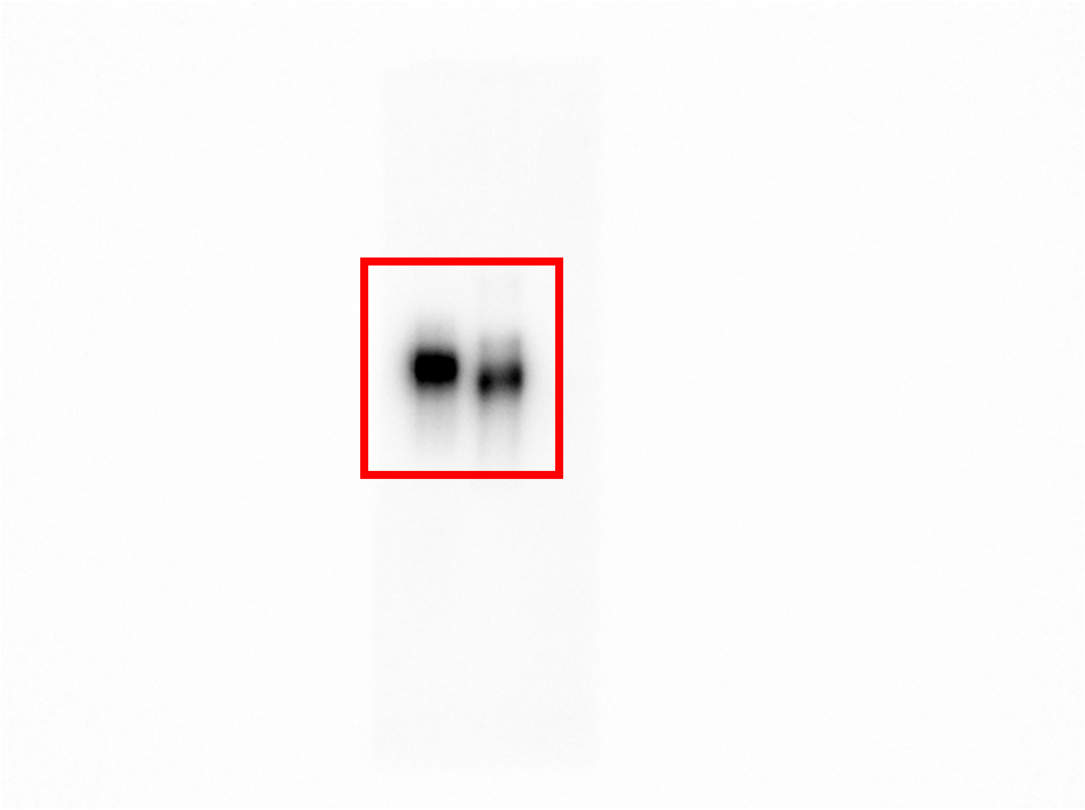

IP: URAT1
WB: URAT1

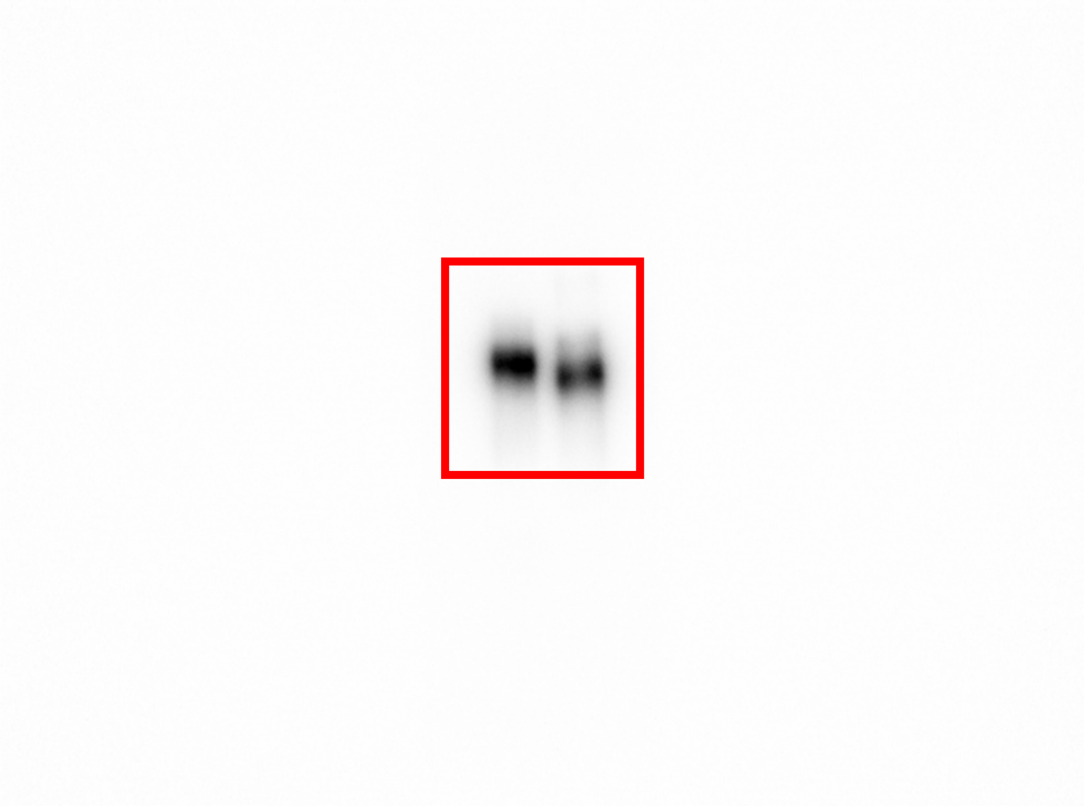

Figure 2E

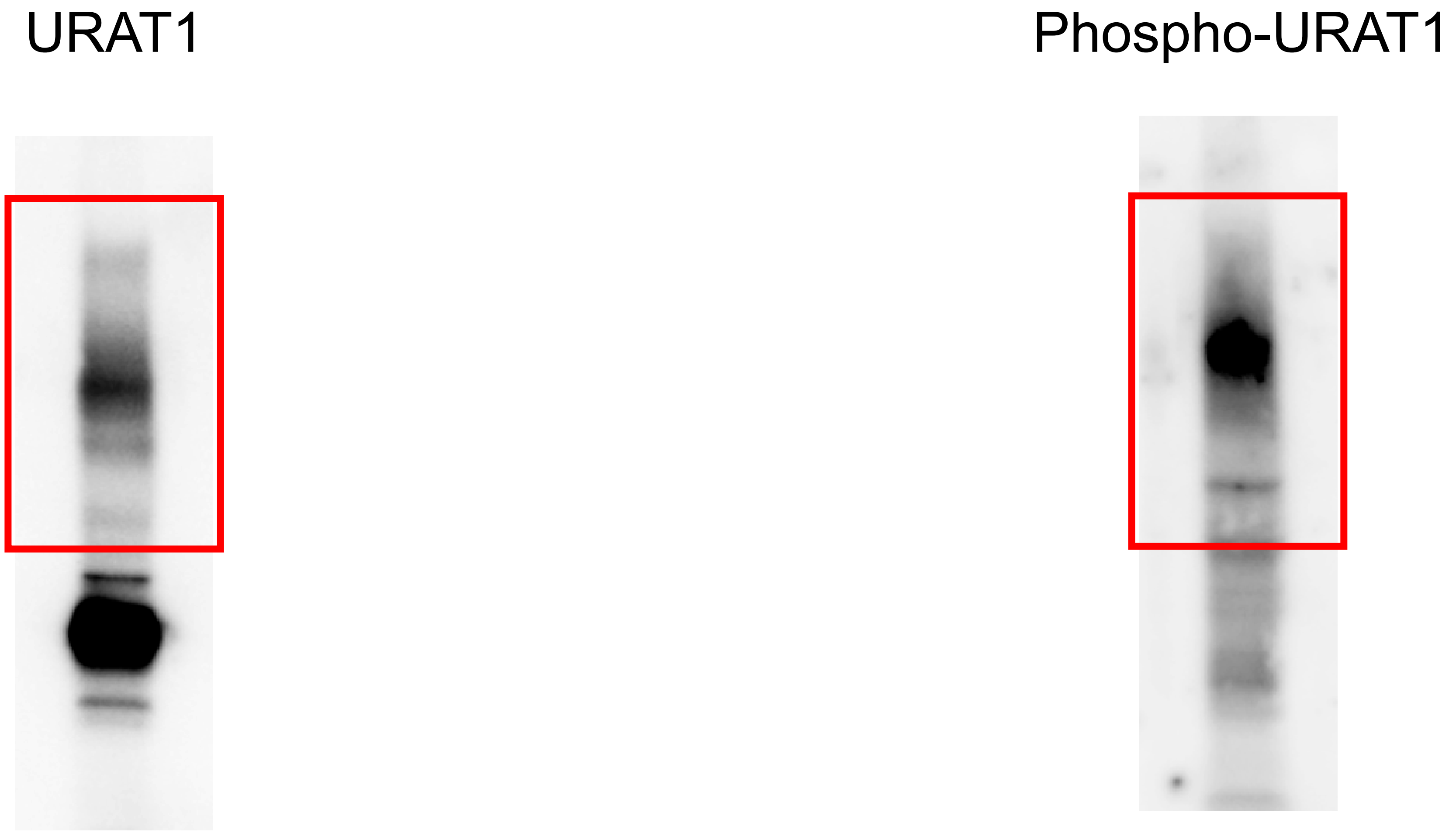

Figure 2G

pAKT (S473)

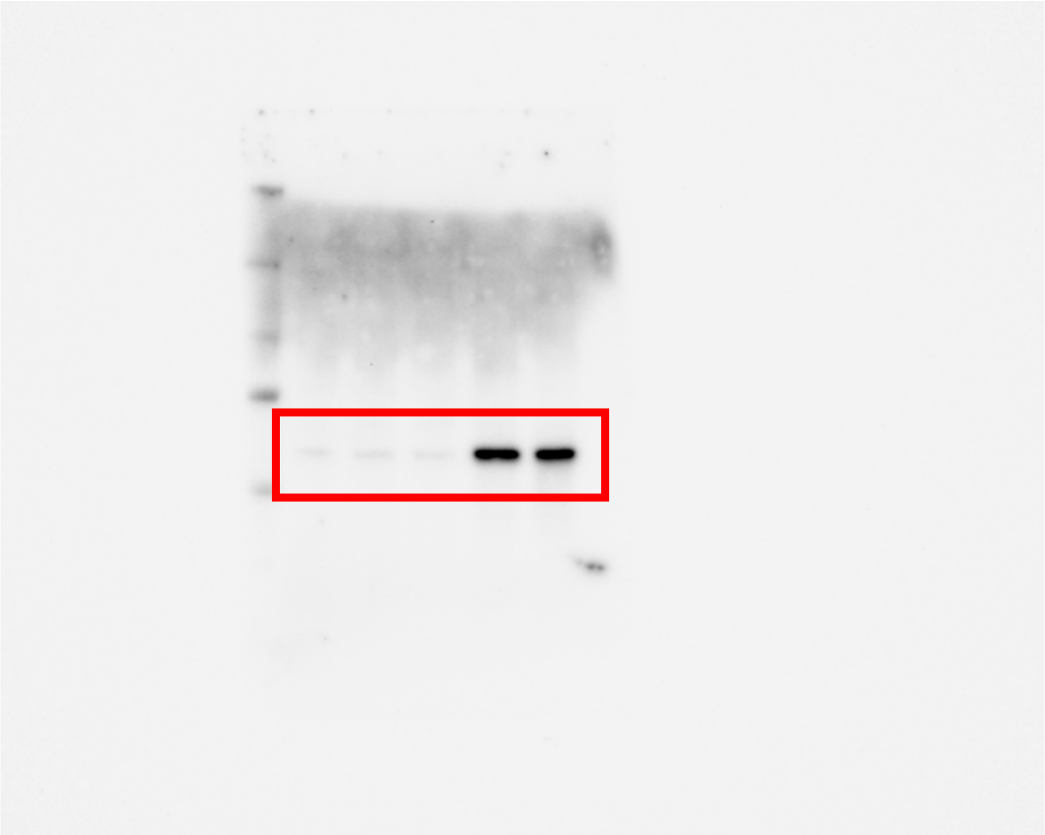

AKT

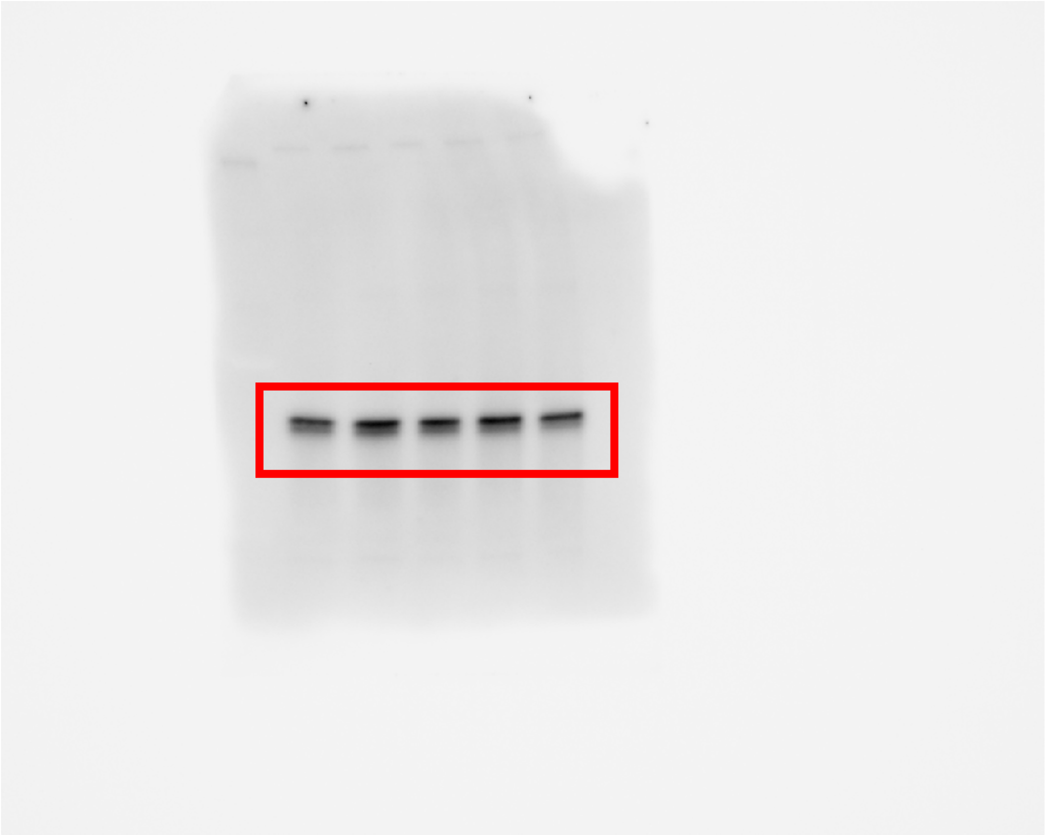

Figure 2H

IP: Flag
WB: Phospho-αURAT1

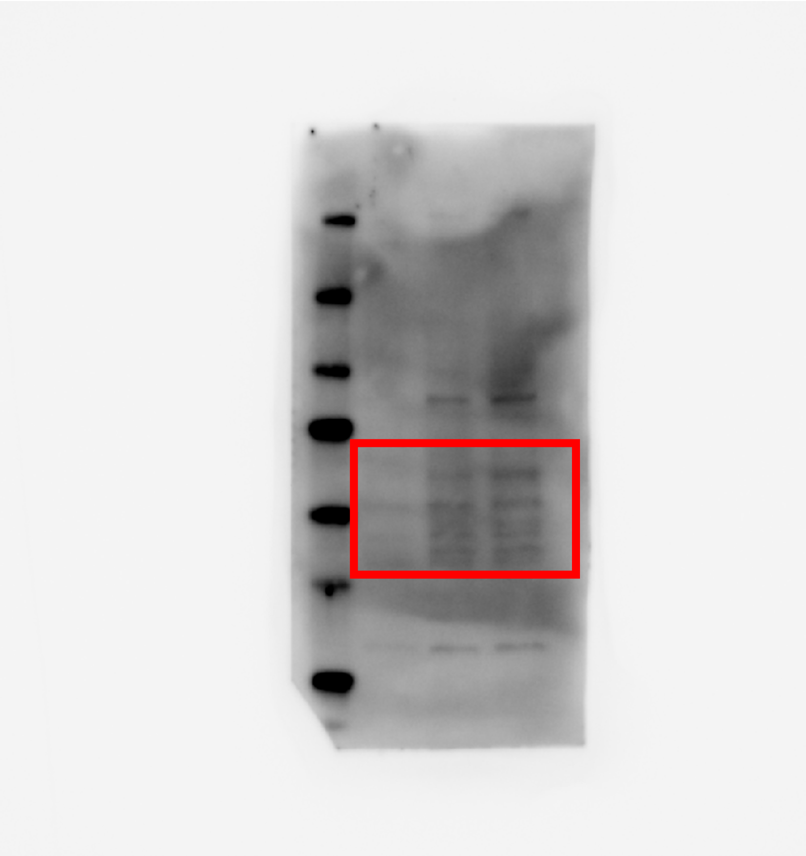

IP: Flag
WB:Flag

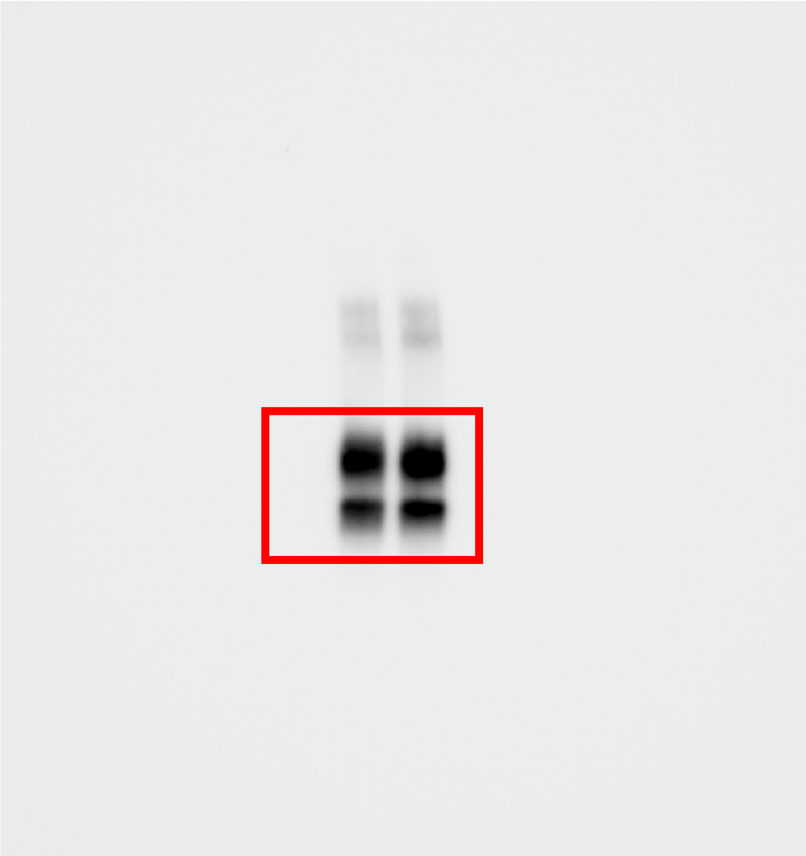

Figure 2I

URAT1

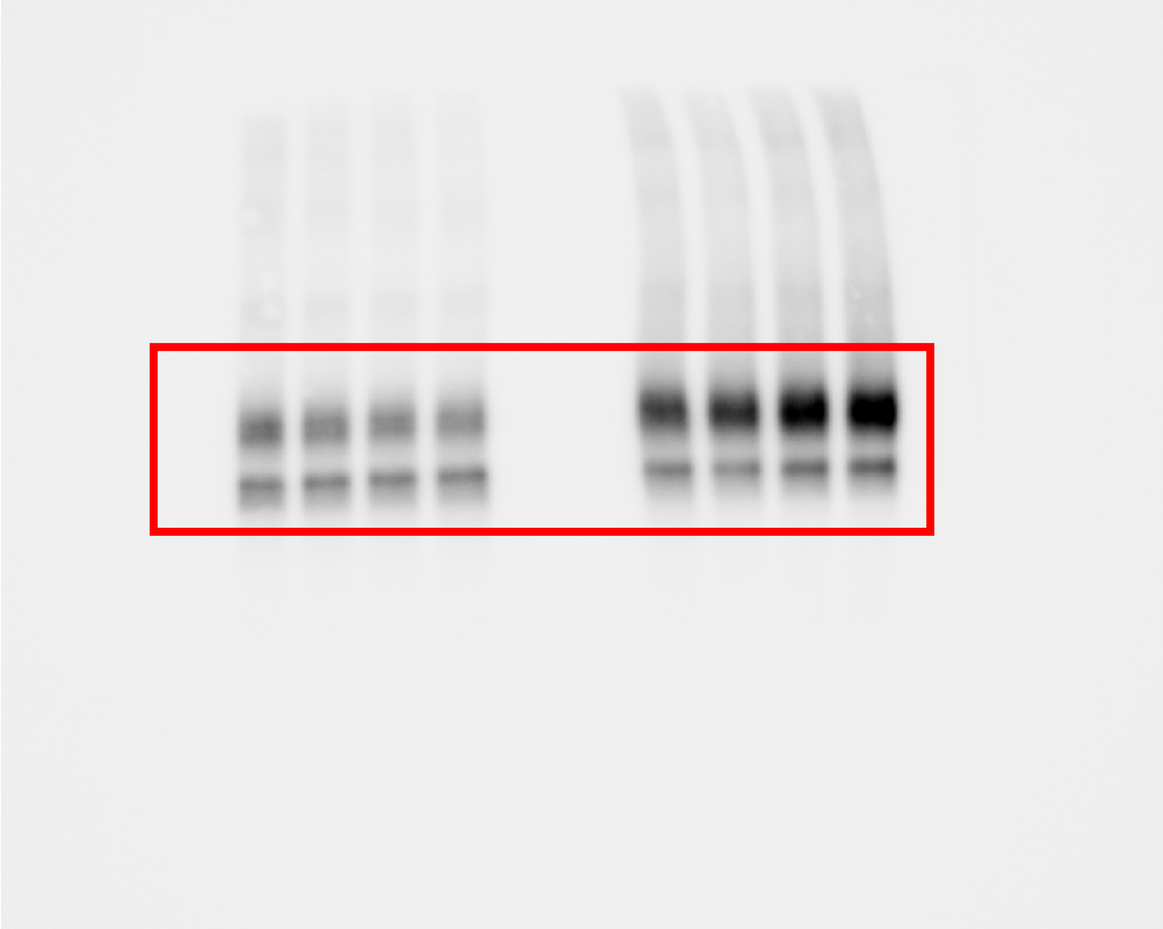

Cadherin

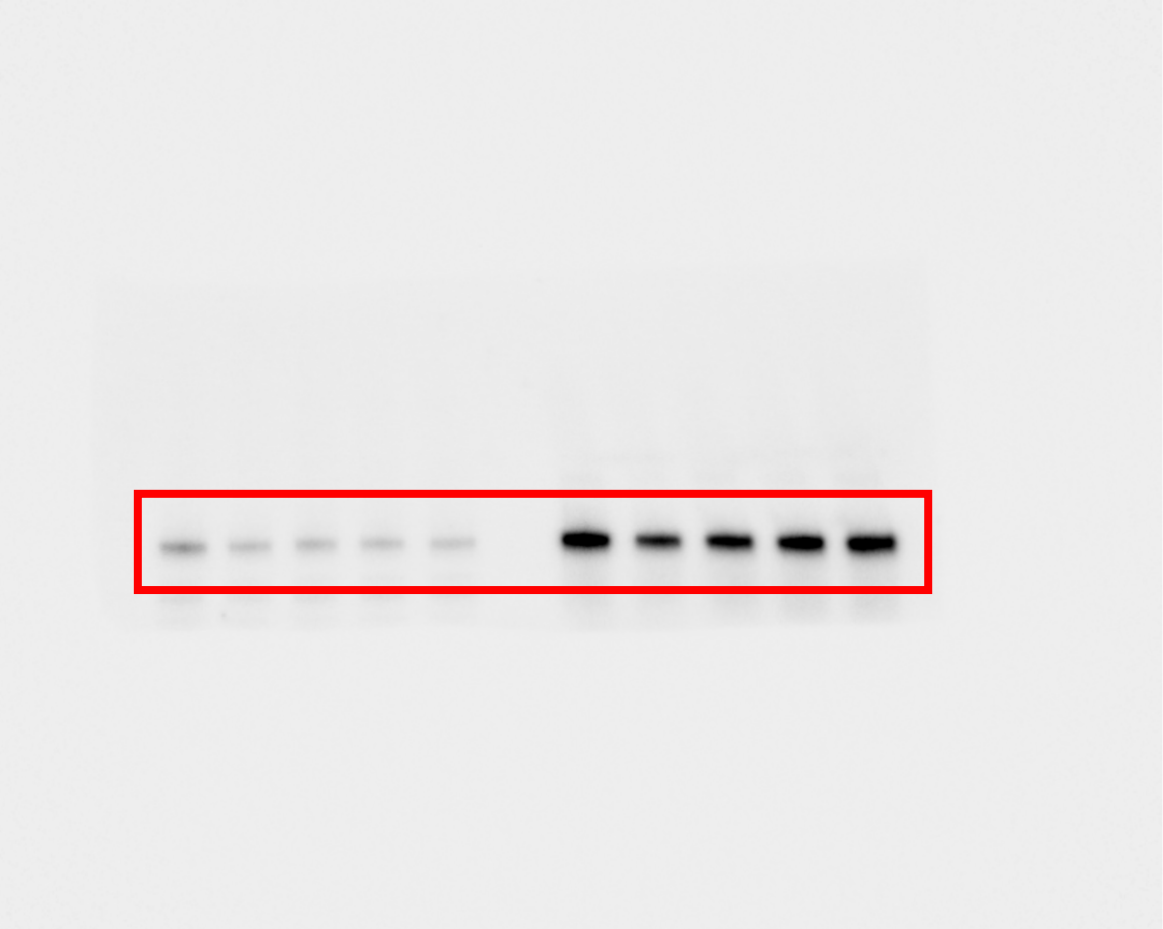

Figure 2J

URAT1

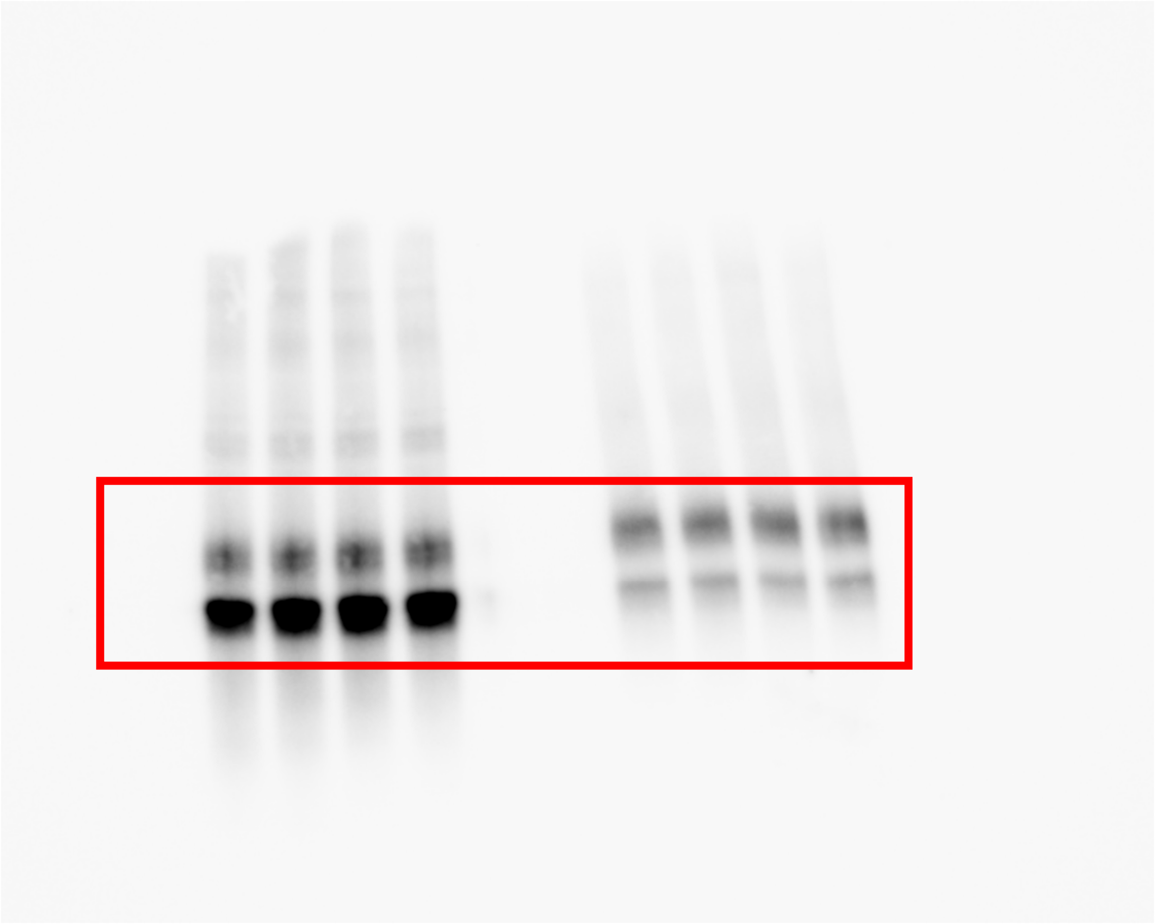

Cadherin

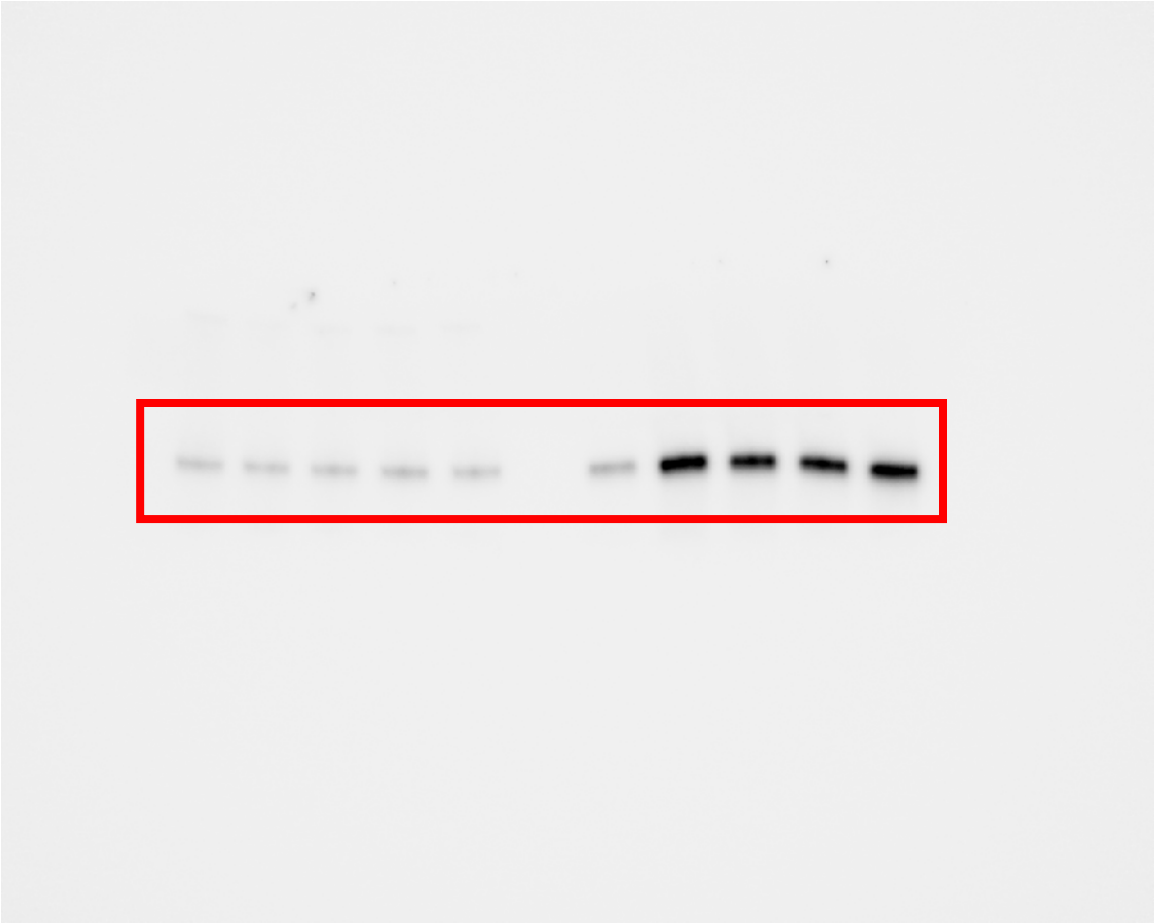

Figure 3D

SGK1

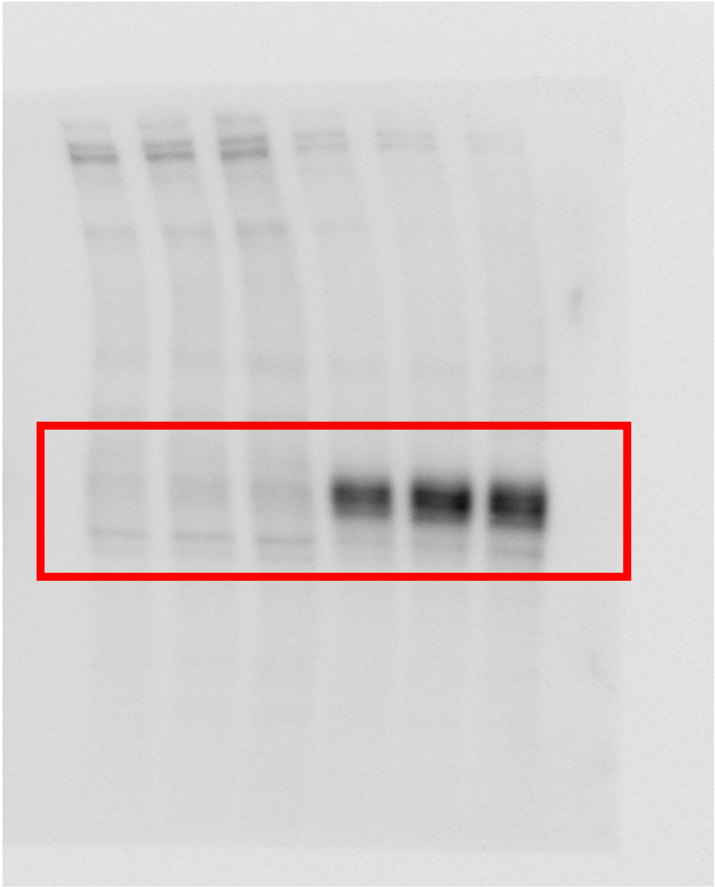

Tubulin

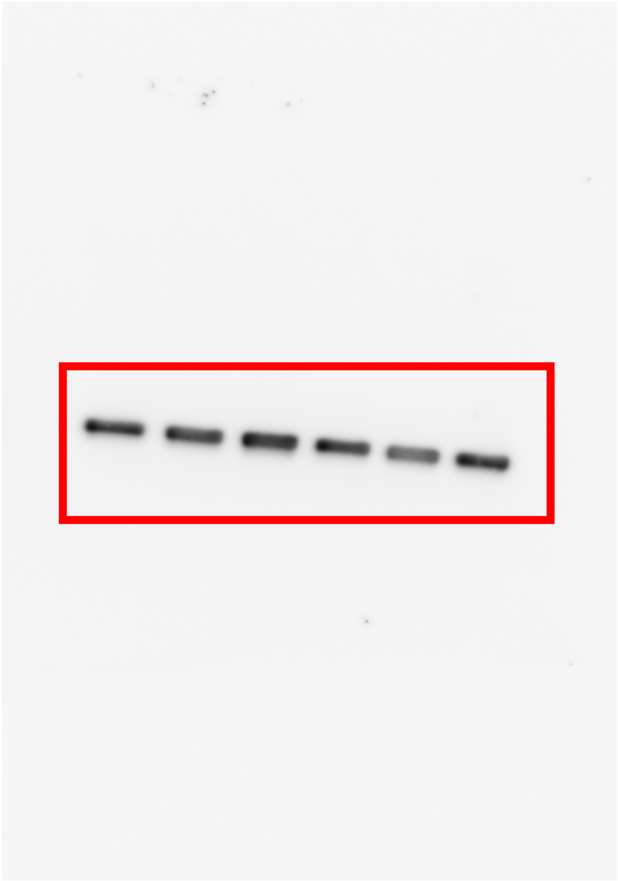

Figure 3E

IP: Flag
WB: Phospho-αURAT1

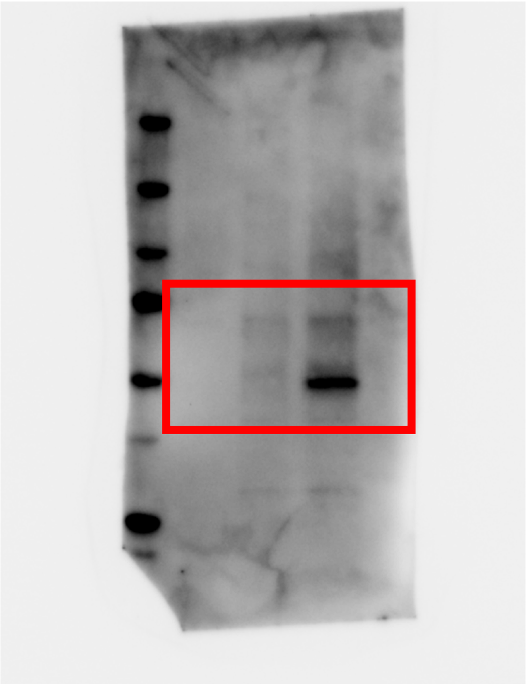

IP: Flag
WB:Flag

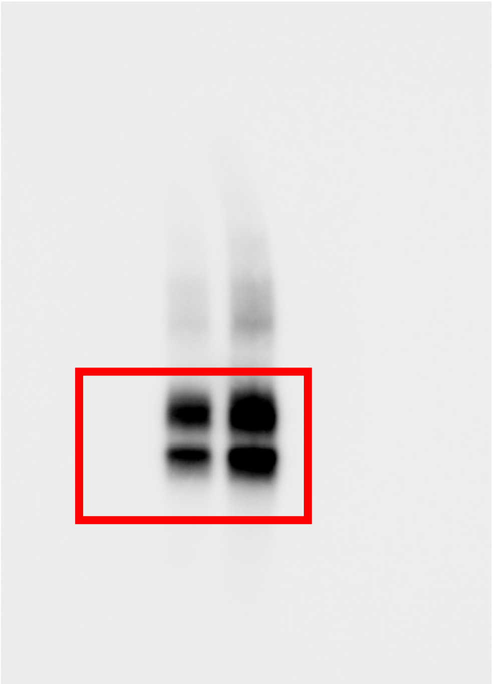

Figure 3F

URAT1

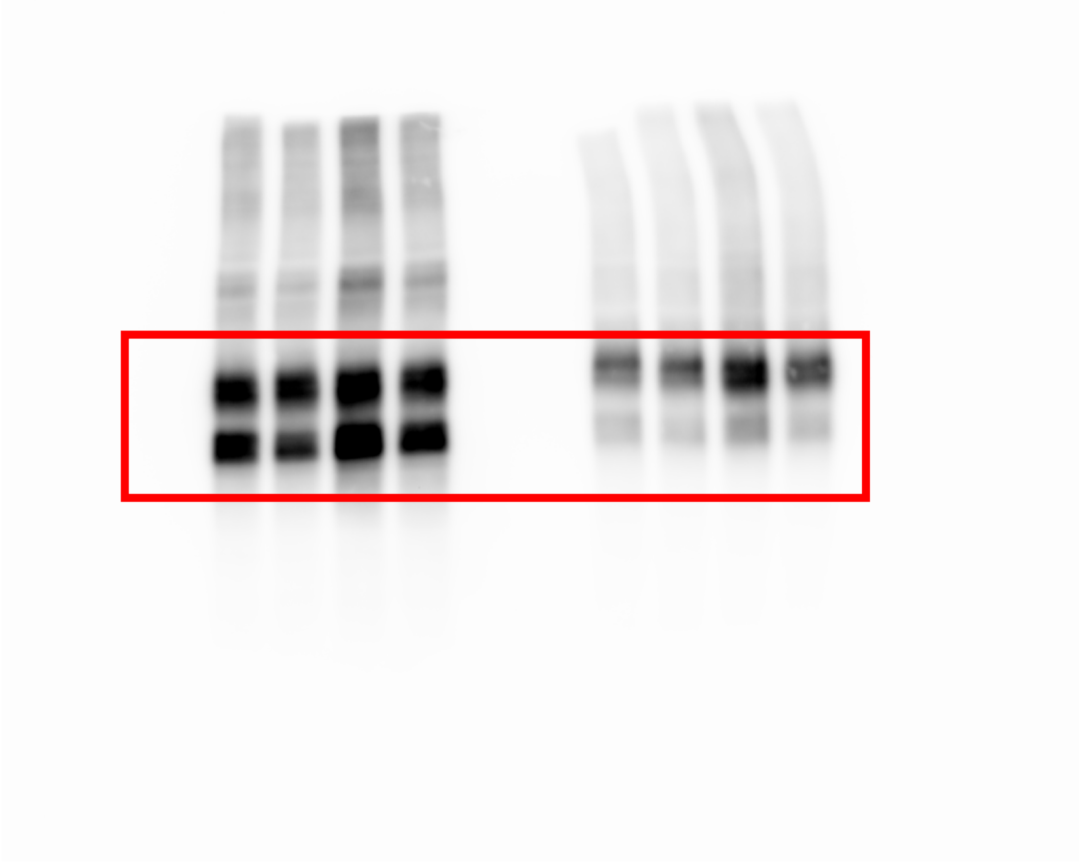

Cadherin

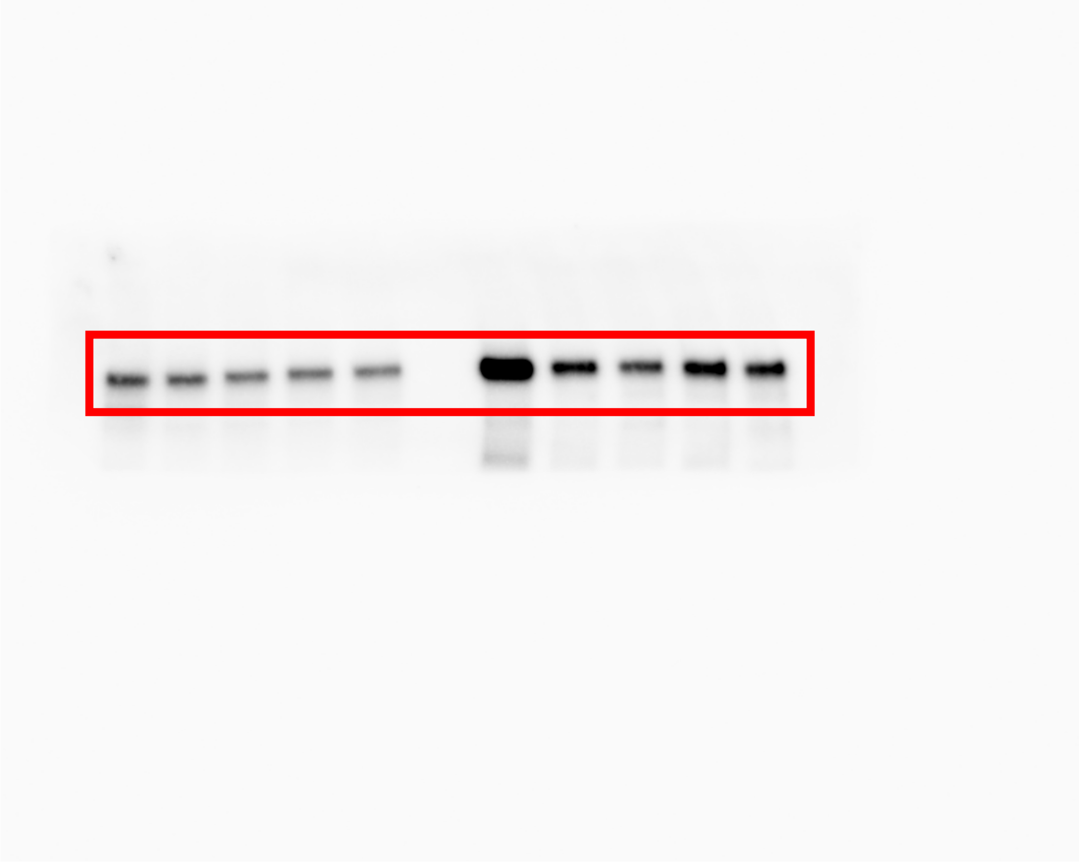

Figure 3G

URAT1

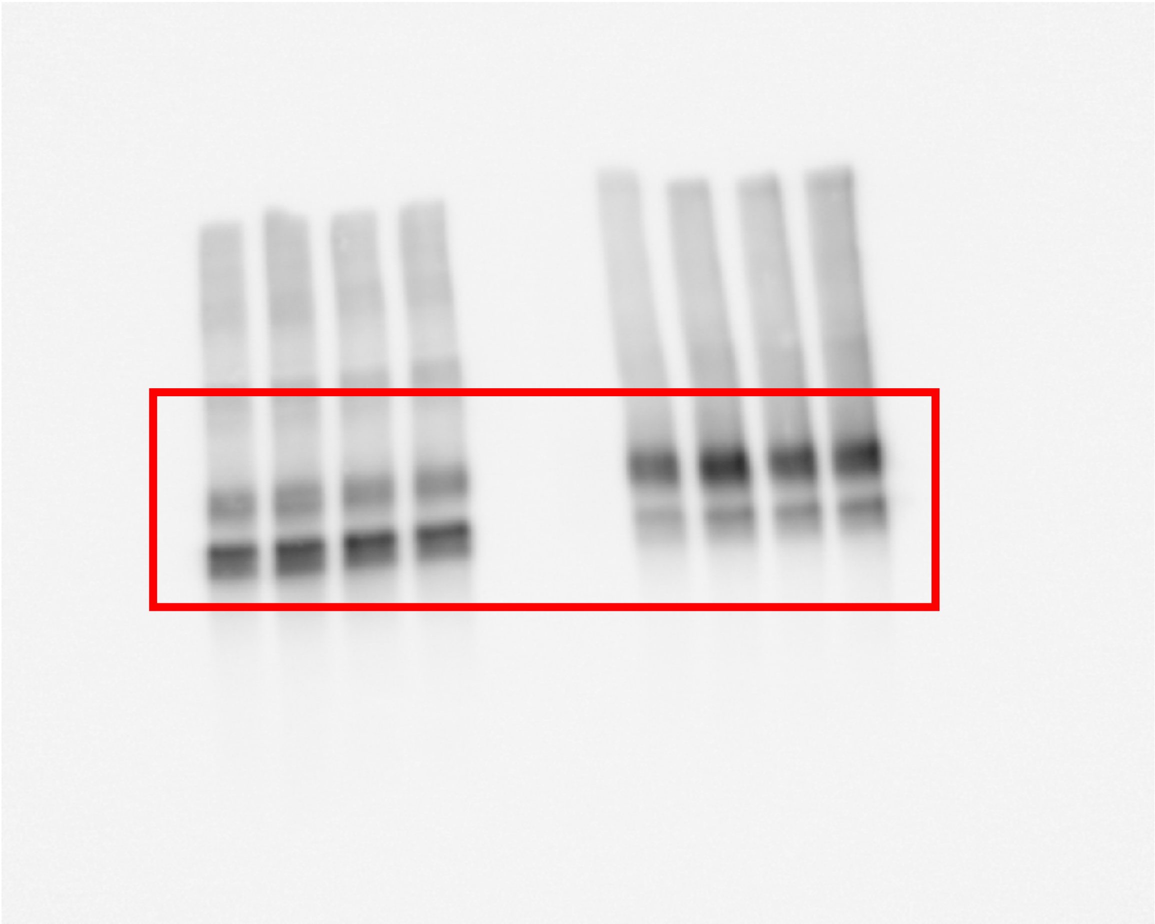

Cadherin

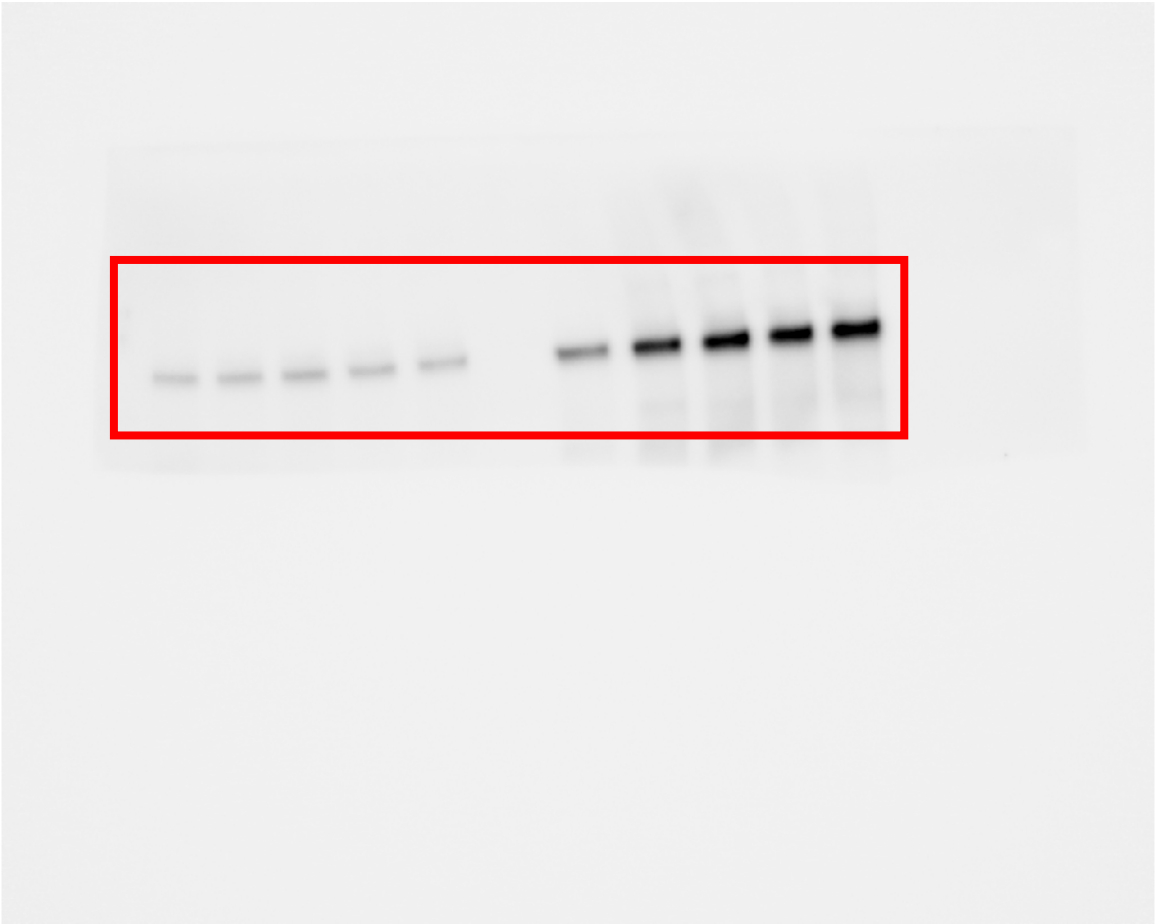

Supplemental Figure S2

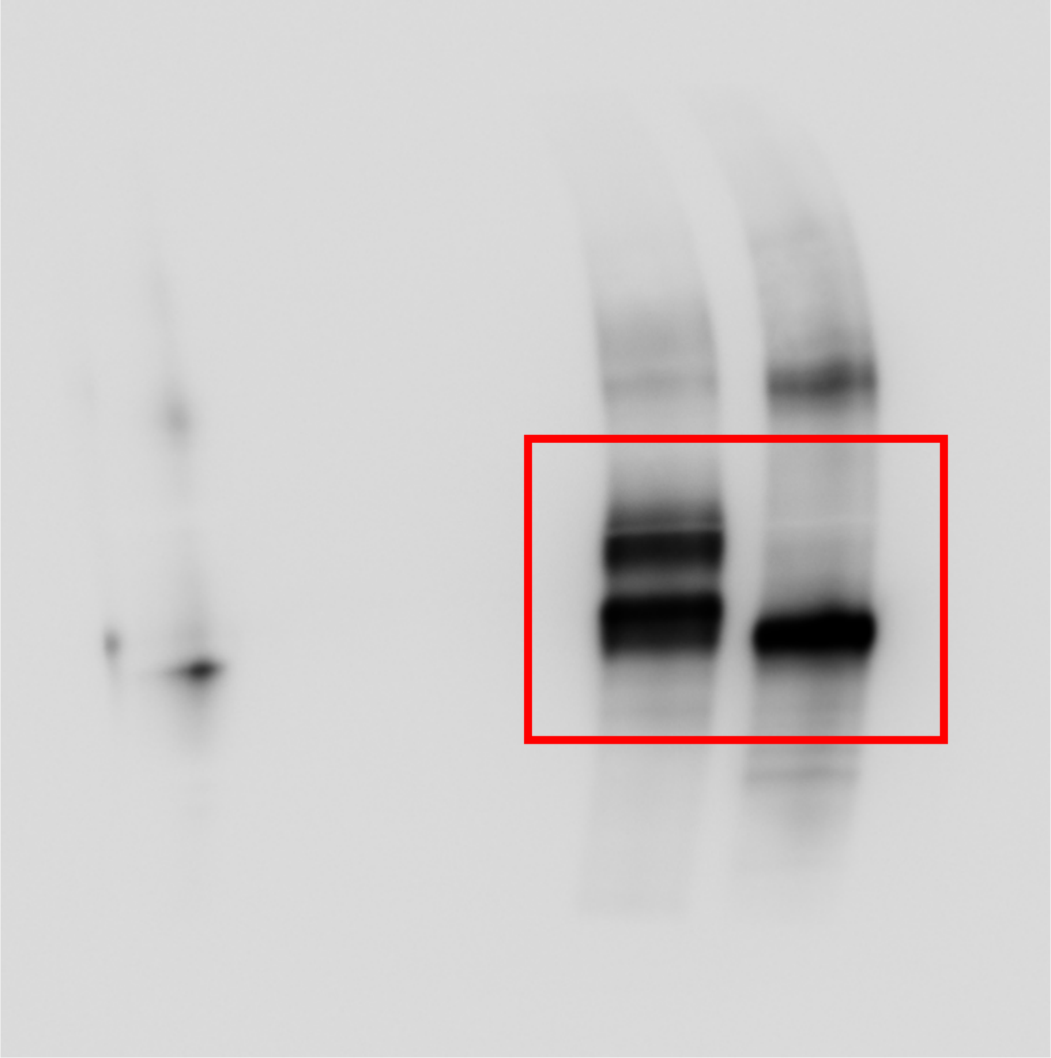

Supplemental Figure S4A

URAT1

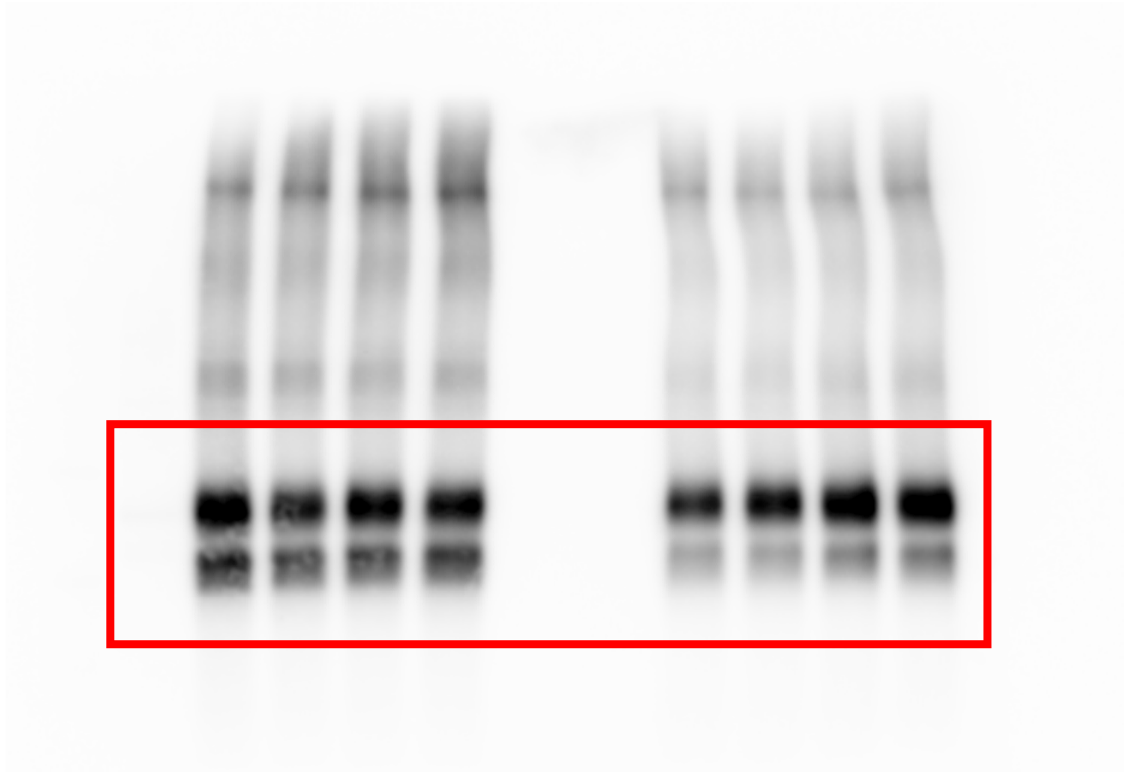

Cadherin

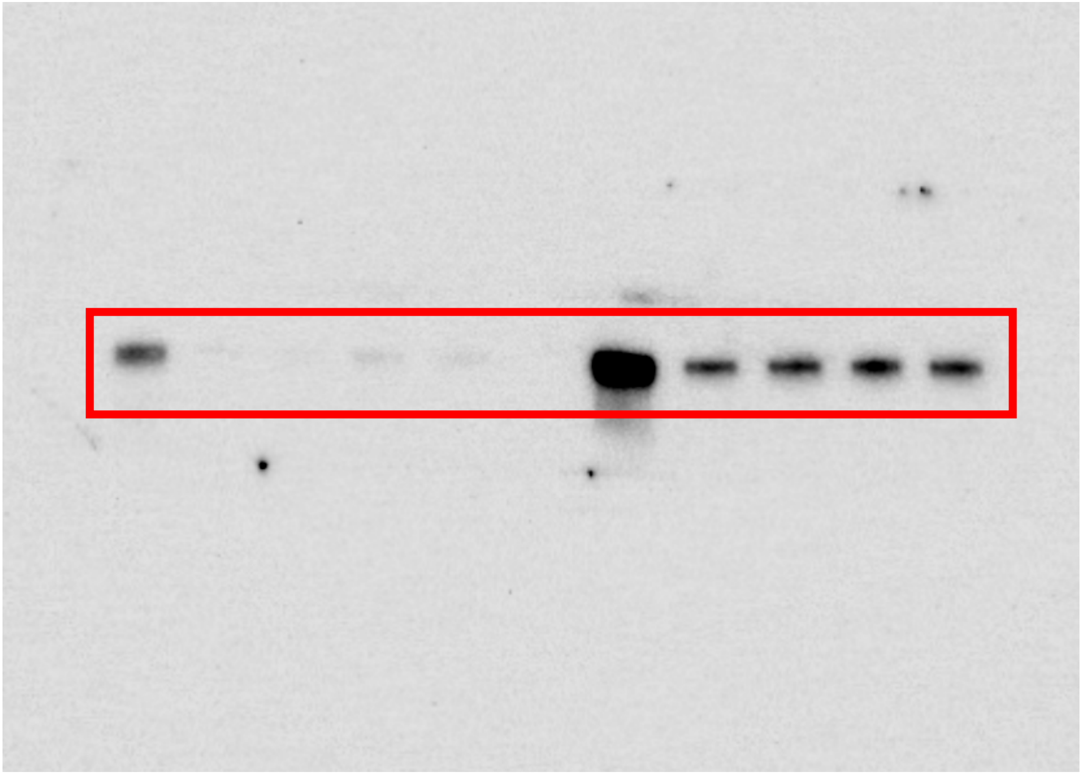

Supplemental Figure S4B

URAT1

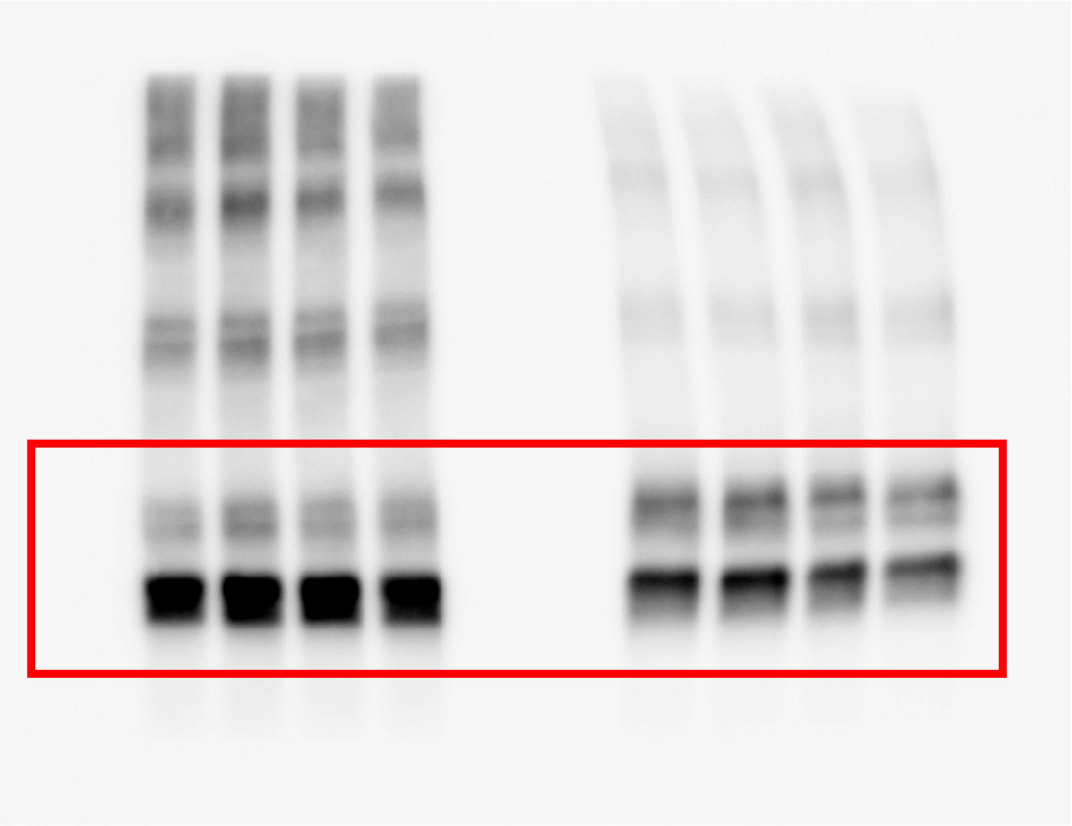

Cadherin

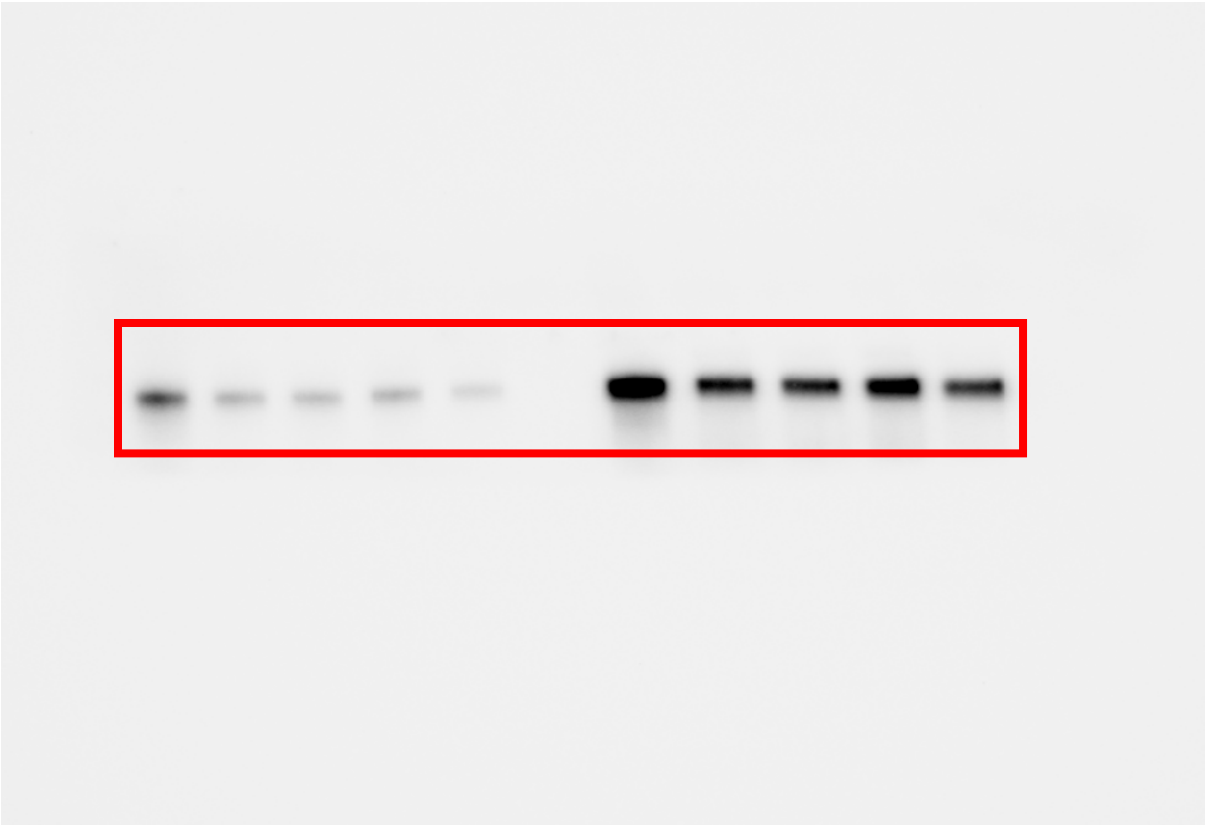

Supplemental Figure S5C

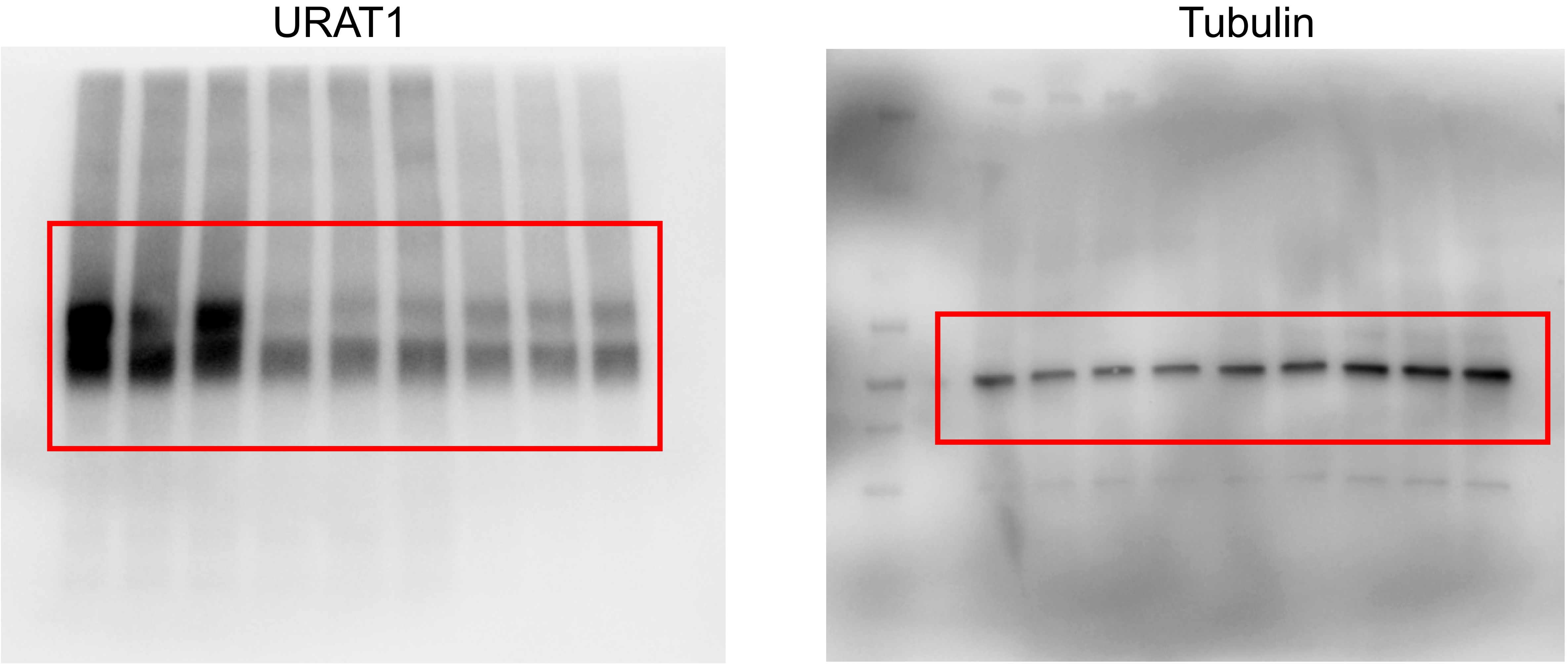

Supplement: Unedited blot and gel images [file jci-135-186633-s022.pdf]
